# RNA sequencing of blood in coronary artery disease: involvement of regulatory T cell imbalance

**DOI:** 10.1186/s12920-021-01062-2

**Published:** 2021-09-03

**Authors:** Timothy A. McCaffrey, Ian Toma, Zhaoquing Yang, Richard Katz, Jonathan Reiner, Ramesh Mazhari, Palak Shah, Michael Tackett, Dan Jones, Tisha Jepson, Zachary Falk, Richard Wargodsky, Dmitry Shtakalo, Denis Antonets, Justin Ertle, Ju H. Kim, Yinglei Lai, Zeynep Arslan, Emily Aledort, Maha Alfaraidy, Georges St. Laurent

**Affiliations:** 1 Division of Genomic Medicine, Department of Medicine, The George Washington Medical Center, The George Washington University, 2300 I Street NW, Ross Hall 443A, Washington, DC 20037 USA; 2 Division of Cardiology, Department of Medicine, The George Washington University , Washington, DC 20037 USA; 3 SeqLL, Inc., Woburn, MA USA; 4 The St. Laurent Institute, Vancouver, WA USA; 5 Department of Statistics, Biostatistics Center, The George Washington University, Washington, DC 20037 USA; 6 Department of Microbiology, Immunology, and Tropical Medicine, The George Washington University, Washington, DC 20037 USA; 7 Department of Clinical Research and Leadership, The George Washington University, Washington, DC 20037 USA; 8 A.P. Ershov Institute of Informatics Systems SB RAS, 6, Acad. Lavrentjeva Ave, Novosibirsk, Russia 630090; 9 Inova Heart and Vascular Institute, Fairfax, VA USA; 10 True Bearing Diagnostics, Washington, DC 20037 USA

**Keywords:** Atherosclerosis, Transcriptome, RNA sequencing, Regulatory T cells, Treg, FoxP1, FoxP3, Biomarker, Coronary artery disease, Stress granules, Cilia, Immune synapse

## Abstract

**Background:**

Cardiovascular disease had a global prevalence of 523 million cases and 18.6 million deaths in 2019. The current standard for diagnosing coronary artery disease (CAD) is coronary angiography. Surprisingly, despite well-established clinical indications, up to 40% of the one million invasive cardiac catheterizations return a result of ‘no blockage’. The present studies employed RNA sequencing of whole blood to identify an RNA signature in patients with angiographically confirmed CAD.

**Methods:**

Whole blood RNA was depleted of ribosomal RNA (rRNA) and analyzed by single-molecule sequencing of RNA (RNAseq) to identify *tr*anscripts *a*ssociated with *C*AD (TRACs) in a discovery group of 96 patients presenting for elective coronary catheterization. The resulting transcript counts were compared between groups to identify differentially expressed genes (DEGs).

**Results:**

Surprisingly, 98% of DEGs/TRACs were down-regulated ~ 1.7-fold in patients with mild to severe CAD (> 20% stenosis). The TRACs were independent of comorbid risk factors for CAD, such as sex, hypertension, and smoking. Bioinformatic analysis identified an enrichment in transcripts such as FoxP1, ICOSLG, IKZF4/Eos, SMYD3, TRIM28, and TCF3/E2A that are likely markers of regulatory T cells (Treg), consistent with known reductions in Tregs in CAD. A validation cohort of 80 patients confirmed the overall pattern (92% down-regulation) and supported many of the Treg-related changes. TRACs were enriched for transcripts associated with stress granules, which sequester RNAs, and ciliary and synaptic transcripts, possibly consistent with changes in the immune synapse of developing T cells.

**Conclusions:**

These studies identify a novel mRNA signature of a Treg-like defect in CAD patients and provides a blueprint for a diagnostic test for CAD. The pattern of changes is consistent with stress-related changes in the maturation of T and Treg cells, possibly due to changes in the immune synapse.

**Supplementary Information:**

The online version contains supplementary material available at 10.1186/s12920-021-01062-2.

## Background

There are more than a million heart attacks each year, and 2200 Americans die of cardiovascular disease each day, about one person every 40 s [1]. Outward symptoms of coronary artery disease (CAD) are chest pain, typically radiating down the left arm, and shortness of breath upon exertion. However, chest pain and dyspnea alone are not particularly specific warning signs. In a prospective analysis of patients presenting with chest pain, ultimately, many cases were determined to be musculoskeletal (20%) or gastroesophageal reflux disease (GERD) (13%), while CAD was diagnosed in only 11% of cases, and the remaining cases were either pulmonary [2], neurological, or idiopathic [3]. The Framingham risk factors of advanced age, male sex, elevated cholesterol, smoking, and hypertension, are good predictors of long term risk (30 yr. risk, C statistic = 0.803) [4], but they are far less accurate in acute clinical settings at determining whether a person has CAD or not (C statistic = 0.667, where 0.5 is random chance) [5]. Thus, there is a tremendous need for improvement in the diagnosis of CAD. From the more than one million cardiac catheterizations yearly, 622,000 result in interventions such as stent placement [6]. Despite the presence of CAD symptoms and other clinical tests suggestive of CAD, 20–40% of angiograms do not detect any occluded arteries [5, 7–10]. The American College of Cardiology’s Registry, covering 398,978 patients, identified 39.2% of patients undergoing invasive coronary angiography (ICA) as having less than 20% stenosis [5]. Thus, reliable blood-based biomarkers of CAD would have the potential to reduce the number of cardiac catheterizations on relatively low risk individuals.

Several prior microarray studies suggested that there is an RNA signature in blood associated with CAD [11–15]. However, the agreement between these studies on exactly which transcripts are modulated is quite low. Such discrepancies could have several explanations, but likely arise from cross-hybridization noise created by highly abundant signals, such as globins, which can overwhelm true signals in microarrays [16], and likely mask changes of low magnitude, or larger changes in a small subset of cells. Thus, the present studies employed a more advanced, single-molecule RNA sequencing (RNAseq) methodology to identify diagnostic transcripts associated with CAD (TRACs). Using RNAseq of whole blood RNA, a novel pattern of gene expression changes was identified that is associated with the presence of CAD, but essentially unrelated to other known risks for CAD. This subset of TRACs is consistent with extensive accumulating evidence for a role of regulatory T cell (Treg) dysfunction as an important component in the etiology of CAD.

## Methods

### Experimental design

The studies take advantage of the fact that up to 40% of patients that undergo invasive coronary angiography (ICA) actually do not have meaningful coronary blockage. The TRACs were identified by comparing the mRNA expression pattern of patients with CAD versus those without CAD. The strength of this model is that blood was taken prior to the catheterization, and the outcome of the angiography becomes known within hours, which provides an ideal learning environment for designing a transcriptome-based test. After the coronary angiograms were digitally interpreted by an attending physician, the patients were divided into 3 groups, ≤ 20% stenosis (LOW CAD), > 20% but < 70% stenosis of any vessel (MID CAD), and ≥ 70% stenosis of any artery (CAD). For power and simplicity, initial analyses compared LOW CAD (< 20% stenosis) to MID+ (> 20% stenosis).

### Patients

#### Discovery cohort

**Fig. 1:**
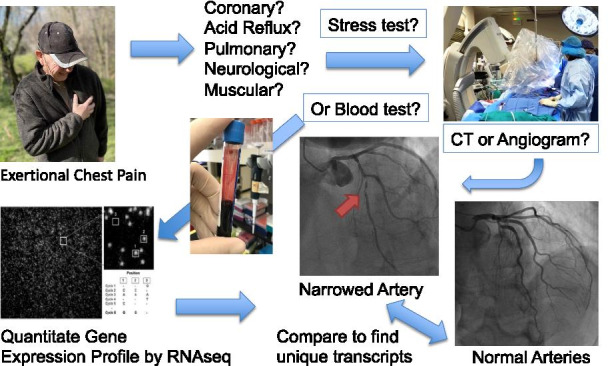
Schematic of study design. Patients presenting for elective invasive coronary angiography (ICA) due to suspicion of CAD were consented to determine whether RNA transcripts in blood could serve as biomarkers for CAD. Typically, patients reported chest pain or shortness of breath upon exertion. The results of the angiogram divided the patients into groups with little to no coronary blockage (< 20%, LOW CAD), or patients in which coronary blockage was detected (> 20%, MID+ CAD). The blood from the patients was frozen in Tempus blood RNA preservative, thawed, extracted for RNA, depleted of residual genomic DNA and ribosomal RNA, and genome-wide RNA transcript counting was performed by RNAseq. The two groups were compared to identify transcripts unique to the CAD patients. Images were created by the authors

Patients presenting for non-emergent complaints of typical or atypical chest pain, exertional dyspnea, or other symptoms suggestive of CAD provided written, informed consent for participation in this study under a protocol approved by the George Washington University IRB. Patients with heart failure, non-ST segment elevation MI, and ST segment elevation MI (STEMI) were excluded from the study. The design of the study is shown schematically in Fig. 1. Patients admitted for cardiac catheterization had three Tempus blood RNA tubes collected by peripheral venipuncture or an indwelling catheter. After blood sampling, these studies were purely observational and did not alter in any way the patient’s clinical course. All relevant clinical data, including a complete blood count (CBC), was captured for comparison to the transcriptomic studies. From an initial enrollment of 113 patients, 96 patients had complete clinical and RNA sequencing data for further analysis.

#### Validation cohort

An independent group of patients were consented at INOVA Fairfax Hospital (Fairfax, Virginia) who were likewise undergoing routine, elective ICA for evaluation of suspected CAD. A total of 80 patients had sufficiently complete clinical and RNAseq data for further analysis.

### Clinical prediction model

Prior to ICA in the Discovery cohort, cardiac medical histories were examined by their attending cardiologists to determine CAD risk factors, as defined by the 2013 ACC/AHA Guidelines on the Assessment of Cardiovascular Risk [17]. Hypertension was defined as a history of untreated blood pressure ≥ 140/90 mmHg and/or treatment with anti-hypertensive medications. Diabetes mellitus was defined by fasting glucose of ≥ 126 mg/dl and/or use of insulin or oral hypoglycemic agents. Dyslipidemia was defined according to National Cholesterol Education Program Adult Treatment Panel III guidelines or by treatment with lipid lowering medication. Current smoking status was defined by active smoking within 3 months of presentation. A family history of CAD was defined as MI or cardiac death in a first-degree relative.

Chest pain was classified according to standard criteria for angina pectoris as described [18]. Typical angina includes substernal, jaw, and/or arm pain upon exertion, and which resolves within 15 min of rest and/or use of nitroglycerin. Atypical angina involves 2 of these symptoms, and patients with non-cardiac chest pain experienced 1 or none of these symptoms. Dyspneic patients were classified as having symptoms of a typical angina.

From these clinical parameters, risk points were accumulated based on age, sex, hypertension, diabetes, symptom type, family history, and smoking status, and then compared to an ordinal risk model to predict likelihood of CAD [18].

### Transcriptome profiling

#### RNA processing

RNA was purified from Tempus stabilized frozen (− 80 °C) peripheral blood samples using Tempus Spin RNA Isolation Kit (ThermoFisher Scientific) according to the manufacturer’s protocol. After rigorous in-solution treatment with 4 Units of DNAse (Turbo DNA-free Kit, Ambion), the typical nucleic acid yield from 2.5 ml Tempus blood tubes averaged ~ 5 µg, with an RNA integrity (RIN) score > 8 (10 is maximal) on Agilent 2100 Bioanalyzer. A fixed amount (4.5 ug) of the DNAsed total RNA was depleted of ribosomal RNA (rRNA) by Ribo-Zero rRNA Removal Kit (Illumina), then concentrated with an RNeasy MinElute column (Qiagen), resulting in ~ 500 ng RNA for sequencing.

#### RNA sequencing

For RNAseq, 100 ng of rRNA-depleted RNA was fragmented and analyzed on a Heliscope true single molecule sequencer (tSMS, SeqLL, Inc.). The raw reads, typically 40 million at 38 bp average length, were then computationally aligned to the human genome using the Helisphere indexDPgenomic aligner [19]. The number of reads that align to each transcript was counted and then corrected for transcript length and differences in total reads obtained per patient. The raw read count was adjusted by the size of the transcript so that long transcripts do not appear more highly expressed than short transcripts, and by the number of total reads per sample to produce “Reads Per Kilobase of transcript, per Million mapped” (RPKM) counts. Thus, RPKM corrects the expression level between samples that have different absolute numbers of reads. RPKM levels were imported into GeneSpring GX14 suite, without additional normalization, to identify transcripts that differ between CAD groups (TRACs). Differentially expressed genes (DEGs) were identified by filtering low expression genes, and then applying a combined p-value/fold change thresholds using a Volcano plot, and Analysis of Variance (ANOV) in Genespring.

### Comparison of blood RNA preservation/isolation methods by droplet digital PCR (ddPCR)

To determine whether TRACs were affected by the type of blood RNA preservation method, three Tempus and three Paxgene tubes were drawn from the same subjects at the same time. The samples were isolated according to manufacturer’s protocols, with the exception that the Paxgene samples were not DNAsed on column, instead using the Turbo DNA-free kits (Ambion) on total RNA as a separate step, so as to be comparable with RNA isolated from Tempus tubes. After DNAse, the samples were repurified with RNeasy MinElute kit (Qiagen) and cDNA was reverse transcribed from 500 ng of RNA using the iScript cDNA synthesis kit (Bio-Rad). The synthesized cDNA was diluted 15× to 7 ng/µl and 5 µl per reaction were used in ddPCR combined with 15 µl QX200 EvaGreen ddPCR Supermix (Bio-Rad) containing 1 µl of 2.5 pmol primers diluted from the original stock of 100 uM (pmol/uL). The ddPCR droplets were generated with Automated Droplet Generator and signals were amplified using the standard ddPCR protocols on a C100 Thermal Cycler (Bio-Rad).

The Paxgene versus Tempus cDNAs were then analyzed with a set of ‘invariant’ PCR targets (beta-actin (ACTB) and alpha-tubulin (TBA1)), selected TRACs (DGKA, DLG1, ICOSLG, IKZF4, SMYD3, TCF3, TRIM28), and selected targets unrelated to TRACs (DEFA3, SELL, SOD2, IL12A). The abundance of each transcript was expressed as a ratio of the copies/20 µl per target in Tempus vs Paxgene samples (n = 4 samples from 3 subjects).

## Results

### Clinical parameters

#### Discovery cohort

From a total of 112 patients enrolled, 96 patients had sufficient RNA quantity and quality, and adequate RNA read depth for further analysis. The clinical parameters of those 96 patients were generally comparable between the LOW and MID+ CAD groups. After correction for multiple testing, there were no significant differences correlated with age, ethnicity, sex, BMI, current smoking, hypertension, dyslipidemia, diabetes, or aspirin use (Table 1). However, there was a trend for the group of LOW CAD patients to be somewhat younger (57.5 yr LOW vs 62.5 yr MID+), and with fewer males (43.8% male LOW vs 56.2% male MID+). To consider any possible confounding variables, we performed separate comparative analysis of all major clinical parameters as regulators of transcript profiles in blood against selected TRACs.

**Table 1:** Patient demographics: discovery cohort

	Coronary artery stenosis				
	Low		MID+		P value*
	Mean	S.E.M	Mean	S.E.M	uncorrected
N per group	48		48		
Age (years)	57.5	1.49	62.5	1.41	0.02*
Race (% minority)	62.5		45.8		0.10
Sex (% male)	45.8		56.2		0.31
BMI	34.7	1.32	31.4	0.97	0.05*
Current smoker (%)	8.3		14.6		0.34
Hypertension (%)	70.8		75.0		0.86
Systolic BP	135.85	3.02	137.05	3.80	0.81
Diastolic BP	72.67	1.66	72.67	1.83	1.00
Dyslipidemia (%)	58.3		70.8		0.28
Total Chol. (mg/dL)	180.50	4.80	167.09	4.91	0.62
LDL Chol. (mg/dL)	109.00	4.49	95.64	4.74	0.61
VLDL Chol. (mg/dL)	13.00	0.61	23.45	1.60	0.23
HDL Chol	51.00	0.61	47.82	1.40	0.67
Tri-glycerides	66.00	3.06	117.45	7.97	0.23
Creatine kinase-(U/L)	152.00	19.60	172.75	19.78	0.87
Diabetes (%)	35.4		33.3		0.83
Aspirin (%)	52.1		62.5		0.21
PTT (s)	29.77	0.60	30.96	0.67	0.19
PT (s)	12.97	0.24	13.13	0.41	0.74
INR	0.99	0.02	0.96	0.01	0.26
WBC (× 10^3^/uL)	6.99	0.35	6.92	0.36	0.89
RBC (× 10^6^/uL)	4.63	0.07	4.37	0.07	0.02*
Hemoglobin (g/dL)	13.19	0.17	13.16	0.24	0.91
Hematocrit (%)	39.79	0.48	39.02	0.62	0.33
MCV (fL)	85.94	0.75	89.01	0.69	0.01*
MCH (pg)	28.45	0.30	30.00	0.26	0.00*
MCHC (g/dL)	33.06	0.17	33.70	0.18	0.02*
RDW (%)	14.16	0.25	13.03	0.27	0.01*
Platelet Count (× 10^3^/uL)	249.81	8.08	228.98	8.84	0.09
MPV (fL)	10.63	0.13	10.57	0.14	0.76
Seg. neutrophils (%)	56.34	1.83	62.30	1.59	0.03*
Lymphocyte %	32.76	1.74	26.21	1.38	0.01*
Eosinophil %	2.34	0.19	2.33	0.26	0.96
Basophil %	0.40	0.07	0.24	0.07	0.17
Abs. seg. neutrophils (× 10^3^/uL)	4.08	0.24	4.53	0.32	0.31
Abs. lymphocytes (× 10^3^/uL)	2.42	0.25	1.72	0.09	0.02*
Abs. eosinophils (× 10^3^/uL)	0.16	0.01	0.16	0.02	0.95
Absolute basophils (× 10^3^/uL)	0.04	0.00	0.03	0.00	0.22
Auto monocyte %	7.53	0.34	8.35	0.39	0.15
Auto monocyte # (× 10^3^/uL)	0.53	0.03	0.56	0.03	0.51
Immature granulocytes %	0.24	0.02	0.25	0.02	0.62
RNA yield (ng/ul) 50 ul total	121.72	11.1113	96.48	7.50	0.07
Yield (ug/2.5 ml tube)	6.09		4.82		
Total RNAseq reads	6.08E+07	4,189,732	5.35E+07	3,691,059	0.19
Filtered reads	2.44E+07	1,876,258	2.02E+07	1,522,361	0.09
Aligned reads (informative)	8.75E+06	773,714	6.65E+06	474,229.5	0.02*

#### Validation cohort

Patients were recruited from an ongoing cohort examining the relationship between DNA variations and CAD. A total of 80 patients had acceptable RNAseq data for further analysis. This suburban Virginia cohort had somewhat different demographics, with mainly the minority composition dropping from more than 50% in Discovery group to less than 20% in the validation group, as shown in Table 2.

**Table 2:** Patient demographics: validation cohort

	Coronary artery stenosis				
	Low		MID+		P value
	Mean	S.E.M	Mean	S.E.M	uncorrected
N per group	37		43		
Sex (% male)	62.16%		51.16%		0.32
Age (years)	62.89	1.74	67.61	1.79	0.06
Race (% minority)	16.22%		9.30%		0.99
Hispanic (%)	5.41%		2.33%		0.47
Height (cm)	173.54	2.12	170.39	1.57	0.23
Weight (kg)	96.11	4.69	86.59	3.25	0.09
BMI	32.01	1.57	29.83	1.04	0.24
SBP	124.62	2.58	127.42	3.02	0.49
DBP	70.19	1.67	68.58	1.40	0.46
MAP	88.35	1.67	88.23	1.68	0.96
HR	69.68	1.95	71.00	2.17	0.66
Heart Rate	69.68	1.95	71.00	2.17	0.66
EF %	54.70	1.67	58.16	1.51	0.13
Dyslipidemia	43.24%		65.12%		0.05*
Hypertension	67.57%		67.44%		0.99
Diabetes mellitus	21.62%		23.26%		0.86
Smoking	5.41%		11.63%		0.33
Aspirin	48.65%		76.74%		0.01*
Creatinine	0.96	0.04	0.98	0.04	0.69
%Stenosis	1.57	0.48	66.79	3.97	6.50E − 25*
RNA yield (ng/ul)	81.36	2.34	84.54	2.15	0.32
Total RNA/tube (ng)	7322.59		7608.50		

### Analytical parameters

The yield of RNA and the number of reads per patient did not vary significantly, when multiple testing was considered (Table 1). Of the 112 samples submitted for sequencing, 16 were excluded due to low yield from RNA purification or ribosomal depletion, inefficient cDNA synthesis, or low yield of usable reads from RNAseq. There was a trend that was not statistically significant when corrected for multiple testing that the LOW CAD patients had slightly higher RNA yield (6.09 µg/tube LOW, vs. 4.82 µg/tube MID + CAD, p = 0.07 uncorrected) and higher read depth (p = 0.02 uncorrected) on RNAseq. Thus, these were considered as possible confounds in subsequent analysis.

### Sources of variation in RNA yield

Patient blood samples collected with either Paxgene or Tempus RNA preservation tubes show a surprisingly large variation in the RNA yield, with Tempus generally producing higher total RNA yield [20]. The total nucleic acid yield from Tempus-preserved samples ranged from 0.6 to 35.0 µg/tube whole blood, with a mean of 10.6 µg per tube of blood, with post-DNAse and MinElute cleanup yield of ~ 5 µg RNA per tube (Table 1). The correlations between total nucleic acid yield and any single blood cell count parameter were quite weak, with only a modest correlation to absolute lymphocyte count (r = 0.55 with N = 112, R^2^ = 0.31) (Additional file 1: Fig. S1). While one outlier with a lymphocyte count of 12 K/µl yielding 35 µg of RNA seems to drive this correlation, the correlation remains modestly positive even when that patient is omitted (r = 0.45 w/o). Thus, the lymphocyte count is the major factor in RNA yield, but accounts for only about 30% of the variability.

### Whole blood RNA biomarkers

**Fig. 2:**
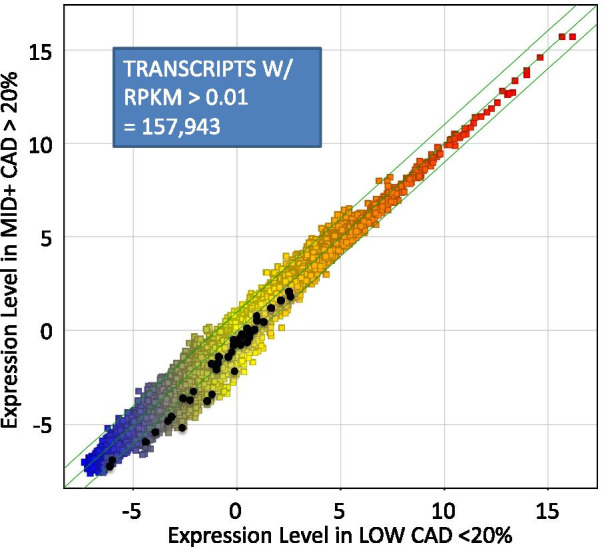
Genome-wide transcript profiling by RNAseq. A total of 96 patients with angiographic results were analyzed by RNAseq of whole blood RNA depleted of ribosomal sequences. The short reads were aligned to the human transcriptome (hg19) and counted per transcript. The raw read counts (*R*) were normalized only by (*P*er) the length of the transcript (*K*) and the total number of reads obtained per patient in millions (*M*) to yield RPKM. The RPKM is expressed on a log_2_ scale and averaged across all patients in the LOW CAD group (n = 48, X axis) versus patients in the MID+ CAD group (n = 48, Y axis). Each point represents one transcript where the RPKM was > 0.01 RPKM in 70% of samples in at least one group (157,943 transcripts). Black points represent a set of transcripts identified as differentially expressed between the 2 groups by a statistical analysis of fold-change and t-test probability (p < 0.001 uncorrected, and fold change > 1.5) resulting in 59 transcripts (49 unique, non-redundant)

The RNAseq data was subjected to minimal normalization, using only the raw RPKM data for analysis. When sequencing was completed, the GRCh37/hg19 assembly was the most fully annotated in our lab. The RNAseq reads were aligned, and then parsed and counted against the 161,038 transcripts in hg19. Transcripts with very low-level expression were filtered by requiring RPKM > 0.01 in 70% of the samples of at least one group, which had a minimal impact on the number of included transcripts (157,943). Dividing the samples by CAD level ≤ 20% (LOW, n = 48) versus > 20% (MID+, n = 48) and averaging across patients yielded the geometric mean expression per group per transcript, as shown in Fig. 2. Remarkably, without any normalization per sample beyond RPKM, the RNAseq data shows excellent linearity over 23 log_2_ orders of magnitude, with the highest level of gene expression observed for hemoglobin B, at an average of RPKM of ~ 65 K (16 in log_2_ scale) in both groups. Compared to typical microarray data, the RNAseq shows less noticeable increases in variability at low levels of gene expression, and no detectable saturation of the signal at very high gene expression.

### Identification of differentially expressed RNA biomarkers for CAD

The 96 samples in the Discovery cohort were divided into LOW CAD (< 20% stenosis, N = 48) versus MID + CAD (> 20% stenosis, N = 48). The transcripts were filtered to exclude low expression transcripts (< 0.01 RPKM) and then compared by ANOV (p < 0.001) to identify a small set of differentially expressed genes (DEGs). This initial filtering identified 198 transcripts that include the 59 transcripts highlighted in BLACK in Fig. 2 and detailed in Additional file 2: Table S1.

By filtering the 198 transcripts for those which had a > 20th percentile absolute level of expression (RPKM percentile) in both groups, the list was narrowed to 96 transcripts with higher absolute expression. Of those 96 transcripts, 51 showed a greater than 1.4-fold decrease in the MID + CAD group, which became the parent list for identifying smaller sets of CAD-related transcripts. This combined fold-change/t-test strategy has been established in large, multicenter control studies using spiked samples as a reliable approach to identify true differences [21].

### Comparison of TRACs relative to transcripts related to clinical risk factors

Because CAD has several known risk factors, such as hypertension, smoking, and dyslipidemia, the relationship of TRACs to these other parameters was determined. While not strictly statistically significant, the demographic analysis suggested that the LOW CAD group tended to be younger, heavier, and more female. For comparison purposes, classifying the 96 patients by sex (48 M, 48 F) irrespective of CAD status, and using a combined filter for > threefold change and p < 0.05 (uncorrected), the analysis identified 84 transcripts that were ‘sex-specific’ (Additional file 2: Table S2). This included transcripts from the X (XIST) and Y chromosomes, and yielded an PLSD prediction model that was 97% accurate (100% accurate for males, 95% for females), simply confirming that the RNAseq data can readily detect obvious biological differences.

**Fig. 3:**
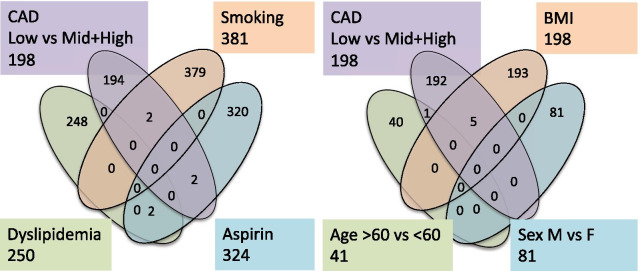
Relationship between TRACs and transcripts identified for clinical risk factors. To determine whether the TRACs (CAD, LOW vs MID+ High, 198 transcripts) were sensitive to known risk factors for CAD, the 96 patients were separated into new groups based on their current smoking (yielding 381 transcripts), aspirin use (324), dyslipidemia (250), age (41), sex (81), and BMI (198). In the case of age, sex, and BMI (right cluster), only the LOW CAD patients were analyzed (n = 48) to prevent confounding with CAD

Using a similar approach, RNA biomarkers lists were constructed for age (young < 60 YO), hypertension, dyslipidemia, BMI, smoking, diabetes, and aspirin use. While each biomarker list showed interesting changes in RNA expression levels, there was very minor overlap with the TRAC list (Fig. 3). Five transcripts (ABCF2, CHST10, FAM129C, MAST4, TEX41) were sensitive to CAD and BMI, even though, to minimize confounding, the BMI list was derived from LOW CAD patients only. SMYD3 was identified as sensitive to the age of the subject, albeit with an alternative transcript ID compared to the TRAC list. However, the direction of the change, whereby SMYD3 increased with age, is opposite to the change expected on the basis of the age of the patients with CAD, and thus, age is somewhat offsetting the CAD effect on SMYD3. Aspirin use was more common in the MID+ group, but its correlation with TRACs was statistically non-significant. Two transcripts were identified that were both TRACs and aspirin-sensitive: CHST10 (decreased only at 81 mg/day dose) and NT5C3B (decreased only at 325 mg/day dose). In general, however, there was little to no evidence that the TRACs are related to other known clinical correlates of CAD.

### Relationship of TRACs to demographic/clinical predictors of CAD

Further, a statistical covariate analysis was conducted, observing that within the LOW or MID + groups, the TRACs were not significantly affected by the clinical variables. The CAD status was highly significantly related to TRAC score (p = 7.78E−11), while among the other risk factors for CAD, only age was a significant factor for TRAC score (p = 0.012), thus confirming that the TRACs appear to be largely independent of known risk factors for CAD in this cohort (Additional file 1: Fig. S2). Exploring the bivariate relationship between age and the TRAC score, there is a slightly negative slope of − 1.22 and R^2^ of 0.056 (p = 0.021, n = 96, Additional file 1: Fig. S3). This impact of age is consistent with the use of age and sex in other gene expression models of CAD [22].

### Relationship of TRACs to analytical variables

In addition to the clinical covariates, the potential contribution of analytical/technical variables was considered. Two factors were identified that might affect the types of transcripts: 1) the MID+ patients tended toward lower RNA yield and 2) fewer informative (non-rRNA) reads (LOW = 8.7 M reads, MID+ = 6.7 M reads, p = 0.02). The likely cause of this difference is the observed difference in lymphocyte counts between groups, which is the primary source of RNA yield (Additional file 1: Fig. S1), and potentially in read depth. To determine whether read depth could contribute to the DEGs, the patients with read depth of < 5 M informative reads (n = 25) were compared to patients with > 5 M reads (n = 71) and analyzed in a similar manner for DEGs. Not surprisingly, a large number of differentially expressed transcripts were identified (1008). However, only 8 transcripts from the 198 TRAC list were sensitive to read depth (APOL4, APTX, C5ORF60, HIF3A, MYO19, NPAS2, RRP12, TMEM67), and this is somewhat confounded by an increased number of MID+ in the low depth group. Thus, it is unlikely that read depth explains the observed pattern of expression in the TRACs.

### Interpreting the TRAC signature

To understand the TRAC signature, the 198-transcript list (Additional file 2: Table S1), generated by analysis of the complete cohort, was subjected to an in-depth analysis. A surprising finding was that 195 of 198 transcripts (98.5%) were down-regulated in the MID and HIGH (MID+) CAD patients, a pattern that rarely occurs in RNA expression analysis, where there is typically a balance between increased and decreased transcripts. Furthermore, the changes are essentially all of the same magnitude (mean =  ~1.7 fold). A similar, but slightly less stringent analysis, using a T-test/fold change filter between the LOW vs MID+ groups identified 461 transcripts, largely overlapping with the 198-gene list, but containing some additional markers of interest, including FLYWCH1, as discussed below (Additional file 2: Table S3).

### Discriminant ability of TRACs for clinical CAD

A partial least squares discriminant (PLSD) model build on these 198 transcripts was very accurate at discrimination between groups, showing an overall accuracy of 98.9% (100% for LOW, 97.9% for MID+). This remained fairly robust even with N-fold internal validation, yielding overall accuracy of 80% (77% for LOW, 83% for MID+). Using a smaller 96 transcript set, with higher fold change, did not improve the predictive ability of the PLSD model built on it, with overall accuracy of 93% (92% for LOW, 94% for MID+), but still produced a quite powerful test, with fewer transcripts. However, these complex polynomial models are able to fit almost any classification, and thus, to minimize ‘over-fitting’, a much simpler linear model was built using predetermined transcripts connected to T cell function.

**Fig. 4:**
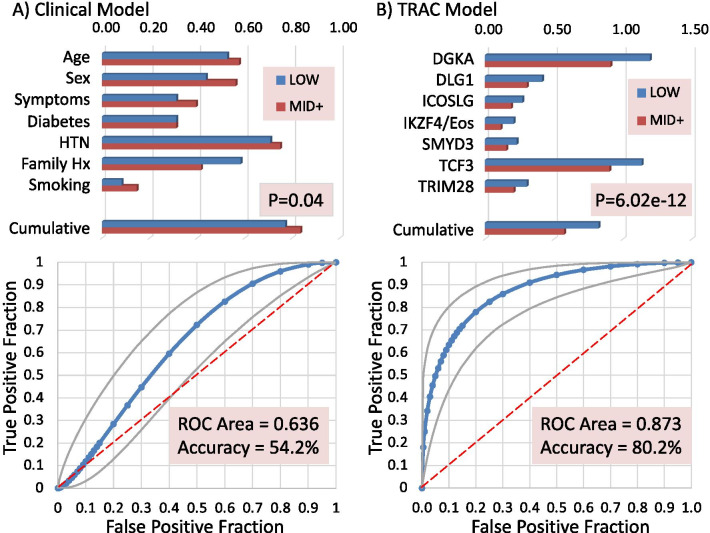
Clinical versus RNA predictors of CAD. **a** Conventional clinical predictors of CAD plotted for each group in the upper panel, showing Age (decades/10), Sex (%Male), Symptom type (typical/atypical), Diabetes (%), Hypertension (HTN, %), Family History of CAD (%), and current Smoking (%). A cumulative CAD risk score is calculated for each patient based on the method of Min et al. and divided by 10 for graphic purposes. The relationship between the cumulative risk score and CAD was calculated by the Receiver Operator Characteristic (ROC) and a confusion matrix to identify the accuracy of the method (lower left). **b** The performance of 7 RNA transcripts as their gene symbols (i.e. DGKA, DLG1) expressed as the RPKM by CAD group. A cumulative score was computed expressing each transcript as a ratio to the mean of its expression in the entire group, to prevent highly expressed transcripts from being over-represented. For graphic purposes, the TRIM28 and Cumulative scores are /10. In the lower panel, the relationship between the cumulative TRAC score (constant-TRAC, to create positive ROC) and angiographically-confirmed CAD is analyzed by ROC similar to the clinical model for the 48 patients in each group

This smaller linear model employed 7 transcripts based on known relevance to T cell function (DGKA, DLG1, ICOSLG, IKZF4/Eos, SMYD3, TCF3, TRIM28) that were normalized to their average expression level, and then an average composite score was calculated (Fig. 4, Upper Right Panel). The composite score of 7 transcripts was highly significant between groups (p = 6.02 × 10^−12^), and a simple linear prediction model yielded a receiver-operator curve (ROC, via JROCFIT [23]) with a C-statistic of 0.873, sensitivity of 77.4%, specificity of 83.7% and overall accuracy of 80.2%, with a positive predictive value (PPV) of 85.4% and negative predictive value (NPV) of 75.0% (Fig. 4, Lower Right Panel). By comparison, a purely clinical model using 7 predictors had a C-statistic of only 0.636, with 55.6% sensitivity, 53.3% specificity, 54.2% overall accuracy, 41.7% PPV, and 66.7% NPV (Fig. 4, Lower Left Panel). A combined clinical (age) and TRAC model yielded a much stronger C-stat of 0.917.

### Expression changes in relation to GWAS findings

A variety of GWAS studies have been conducted using various types of atherosclerotic disease or strongly related risk factors, and approximately 150 loci have some reported association with CAD. Several of the TRACs were essentially identical to prior GWAS loci containing variants associated with cardiovascular or immune variables (Table 3). For instance, alpha-1-B glycoprotein (A1BG) associates with hepatocyte growth factor levels in the MESA cohort [24], and with adverse cardiovascular outcomes during antihypertensive therapy [25]. C6ORF10 associates with susceptibility to CAD in Chinese Han [26], Cadherin 13 (CDH13) has SNPs which associate with multiple CAD risks [27, 28], COMM domain-containing 5 (COMMD5) has been identified in rodent models as associated with hypertension [29], the fibrillin 3 locus (FBN3) associates with metabolic syndrome in the Framingham cohort [30], and the FCH and double SH3 domains 2 (FCHSD2) locus has been associated with systemic lupus erythematosus (SLE) [31], an autoimmune disease frequently complicated by aggressive atherosclerosis. The methylenetetrahydrofolate dehydrogenase 1-like (MTHFD1L) Rs6922269 SNP predicts mortality after acute coronary syndrome [32], and is a known risk loci for CAD [33]. Phospholipase A2 group 10 (PLA2G10) is also a known CAD risk loci in humans [34] and mice [35]. Psoriasis is a well-established risk for CAD, and the psoriasis susceptibility 1 candidate 1 (PSORS1C1) gene expression is reduced in the present CAD cohort, and its locus is associated with psoriasis [36], rheumatoid arthritis [37], and capillary leak [38], and was recently associated with cardiometabolic parameters [39]. Serpin peptidase inhibitor D (SERPIND1, heparin cofactor II) levels have been associated with in-stent restenosis of peripheral arteries [40], and the staining for SERPIND1 in human coronary lesions was correlated with the degree of atherosclerosis in the PDAY study [41]. Notably, Flywich-type zinc finger 1 (FLYWCH1), was identified by the CARDIOGRAM consortium as a driver eQTL risk loci for CAD in vascular and adipose tissues [42]. Thyroid adenoma associated (THADA) was identified in a functional expression analysis of a human beta cell line as potentially relevant to type II diabetes [43]. Thus, GWAS and expression studies suggest that several of the whole blood mRNA expression changes correspond with previously published SNPs for CAD, or CAD risk factors, such as hypertension, SLE, type 2 diabetes, and psoriasis.

**Table 3:** TRACs with known GWAS or expression associations

Gene name	p value	Fold	Fold	Expression (RPKM)			Symbol	Description	RefSeq
		HvsL	MvsL	HIGH	MID	LOW			
uc061drv.1	2.93E−04	1.01↑	− 4.97↓	− 1.24	− 3.56	− 1.25	A1BG	alpha-1-B glycoprotein	NM_130786
uc059ulu.1	5.07E−04	− 1.57↓	− 2.22↓	− 0.71	− 1.21	− 0.06	BBS2	Bardet–Biedl syndrome 2	NM_031885
uc061wwa.1	6.36E−04	− 1.44↓	− 1.39↓	− 0.29	− 0.23	0.24	BLCAP	Bladder cancer associated protein	NM_001167820
uc063nqr.1	3.79E−04	1.29↑	− 3.35↓	− 5.67	− 7.78	− 6.04	C6orf10	Chromosome 6 open reading frame 10	NM_001286474
uc059xrj.1	1.45E−04	− 2.97↓	− 8.68↓	− 2.86	− 4.41	− 1.29	CDH13	Cadherin 13 transcript variant 5	NM_001220491
uc064rqz.1	8.12E−04	− 1.46↓	− 1.57↓	0.09	− 0.01	0.64	COMMD5	COMM domain containing 5	NM_001081004
uc060std.1	7.00E−04	1.21↑	− 6.37↓	− 2.71	− 5.66	− 2.99	FBN3	fibrillin 3	NM_032447
uc058fep.1	6.63E−04	− 1.37↓	− 1.52↓	− 0.02	− 0.18	0.43	FCHSD2	FCH and double SH3 domains 2	NM_014824
uc058vgw.1	2.07E−04	− 1.69↓	− 1.83↓	− 2.04	− 2.15	− 1.28	MMP17	Matrix metallopeptidase 17	NM_016155
uc063sgp.1	7.99E−04	− 2.35↓	− 6.59↓	− 2.57	− 4.06	− 1.34	MTHFD1L	Methylenetetrahydrofolate dehydrogenase 1L	NM_001242768
uc059rbn.1	8.11E−04	− 1.42↓	− 1.91↓	1.87	1.45	2.38	PLA2G10	Phospholipase A2, group X	NM_003561
uc063mxz.1	3.53E−04	− 4.06↓	− 5.22↓	− 3.46	− 3.82	− 1.44	PSORS1C1	Psoriasis susceptibility 1 candidate 1	NM_014068
uc059xcn.1	9.57E−04	− 1.43↓	− 1.59↓	− 0.58	− 0.73	− 0.06	RFWD3	Ring finger and WD repeat domain 3	NM_018124
uc062bvk.1	8.81E−04	− 1.52↓	− 1.42↓	− 0.80	− 0.69	− 0.19	SERPIND1	Serpin peptidase inhibitor D, heparin cofactor	NM_000185
uc001zwr.5	9.10E−04	1.02↑	1.67↑	0.59	1.30	0.56	SLC12A1	Solute carrier family 12A1	NM_000338
uc064wao.1	3.99E−04	− 1.80↓	− 1.66↓	− 1.08	− 0.96	− 0.23	SLC25A25	Solute carrier family 25 A25	NM_052901
uc063fxo.1	5.55E−04	1.02↑	− 1.81↓	0.47	− 0.41	0.44	SLC25A46	Solute carrier family 25 member 46	NM_001303250
uc062iym.1	2.16E−04	− 2.20↓	− 1.14↓	− 0.19	0.76	0.95	SLC6A20	Solute carrier family 6 (proline transport)	NM_022405
uc061irb.1	5.78E−05	− 2.01↓	− 1.74↓	− 1.11	− 0.91	− 0.10	THADA	Thyroid adenoma associated	NR_073394
uc060vxm.1	4.76E−06	− 3.08↓	− 10.76↓	− 2.48	− 4.28	− 0.86	TMEM161A	TRANSMEMBRANE protein 161A	NM_001256766
uc061you.1	1.46E−04	− 1.45↓	− 1.61↓	− 0.91	− 1.07	− 0.38	ZGPAT	Zinc finger, CCCH-type with G patch domain	NM_181485

### Ontology/pathway analysis of TRACs

The 198-gene list was more fully annotated by both automated and manual literature mining and genome analysis. Several levels of analysis were employed. Initially, because the DEGs tended to all be decreased by a similar magnitude, transcripts were examined to determine whether they were indicative of a particular cell type present in blood that might be associated with CAD. At least 17 of the TRACs were readily associated with T-cell function (Table 4, upper). Notably, CYTIP and PLCG1 have known interactions with the T cell receptor (TCR) signaling [44, 45]. Likewise, DLG1 and PPARA are well established regulators of T-cell function, and TIA1 is an intracellular antigen which marks cytotoxic T-cells [46–48].

**Table 4:** TRACs related to T cell and Treg function

Gene name	p value	Fold HvsL	Symbol	Description	RefSeq
*Related to T cell function*					
uc064mjf.1	8.03E−04	− 1.24↓	AP3M2	adaptor-related Protein 3, mu 2	NM_006803
uc061mig.1	6.28E−04	− 1.21↓	CHST10	Carbohydrate sulfotransferase 10	NM_004854
uc061otq.1	1.03E−04	− 1.47↓	CYTIP	Cytohesin 1 interacting protein	NM_004288
uc058pdy.1	7.75E−04	− 1.36↓	DGKA	Diacylglycerol kinase, alpha	NM_201554
uc062seb.1	9.10E−04	− 1.18↓	DLG1	DISCS, large homolog 1	NM_001204386
uc057gll.1	5.73E−04	− 1.73↓	EPS15	EGF receptor pathway substrate 15	NM_001981
uc063okh.1	1.65E−04	1.12↑	FOXP4-AS1	FOXP4 antisense RNA 1	NR_126417
uc062dbe.1	3.38E−04	− 1.56↓	GATSL3	GATS protein-like 3	NM_001037666
uc059uvj.1	5.04E−04	1.44↑	GPR56	Adhesion G prot-coupled recep G1	NM_001145774
uc062xlo.1	2.87E−04	− 1.15↓	NUP54	Nucleoporin 54 kDa	NR_103781
uc057jvt.1	6.40E−04	− 1.51↓	PHGDH	Phosphoglycerate dehydrogenase	NM_006623
uc061xai.1	5.74E−04	− 1.75↓	PLCG1	Phospholipase C, gamma 1	NM_182811
uc062fel.1	1.70E−04	− 1.09↓	PPARA	perox. prolif. activated rec. a	NM_001001928
uc058jqr.1	9.54E−04	− 1.26↓	RAD52	RAD52 homolog	NM_001297421
uc064bpk.1	5.85E−04	− 1.30↓	SCIN	Scinderin	NM_033128
uc061kij.1	8.52E−04	− 1.45↓	TIA1	Cytotoxic granule-assoc. RNA BP	NM_022173
*Relevant to Treg and/or FoxP3*					
uc063bvz.1	9.62E−05	− 1.25↓	AHRR	Aryl-hydrocarbon rec. repressor	NM_001242412
uc058uor.1	8.95E−04	− 1.38↓	HIP1R	Huntingtin interact. prot.1 related	NM_003959
uc061yty.1	1.48E−04	− 1.62↓	ICOSLG	Inducible T-cell costimulator ligand	NM_001283052
uc058pgk.1	8.59E−04	− 3.36↓	IKZF4	IKAROS family zinc finger 4 (Eos)	NM_022465
uc063ljh.1	9.84E−04	− 1.59↓	IRF4	Interferon regulatory factor 4	NM_001195286
uc063ady.1	5.93E−04	− 1.34↓	LRBA	LPS-responsive, beach anchor	NM_006726
uc057qye.1	2.67E−04	− 1.35↓	SMYD3	SET and MYND domain containing 3	NM_022743
uc057jcy.1	5.62E−04	− 1.55↓	STRIP1	Striatin interacting protein 1	NM_001270768
uc061duf.1	4.36E−04	− 1.33↓	TRIM28	Tripartite motif containing 28	NM_005762
uc060rek.1	4.39E−04	− 1.39↓	TCF3	Transcription factor 3	NM_003200

As shown in the lower half of Table 4, another 10 transcripts suggest that TRACs might be most closely associated with regulatory T cells (Treg). Several strong indications are provided by transcripts such as IKAROS family zinc finger 4 (IKZF4, aka Eos), which is considered a signature transcript for the Treg cell subset [49], and which is important in controlling Treg transition into T-helper (Th) cells [50]. IKZF4/Eos is thought to be a required corepressor for the FoxP3-dependent gene silencing that is necessary for maintaining the stable Treg phenotype [51]. Likewise, Set and Mind domain containing 3 (SMYD3) is also involved in epigenetic control of FoxP3 expression [52]. Further, TCF3, aka E2A, is a major transcription factor controlling FoxP3 expression [53], and TRIM28 has been identified as a member of the FoxP3 transcriptional complex [54]. Given that FoxP3 is considered the hallmark of Treg cells [55], alterations in the expression of these transcripts suggest that changes in the abundance of the Treg population may contribute to the TRAC signature.

### Cell type-specific RNA markers in relation to CAD level

**Fig. 5:**
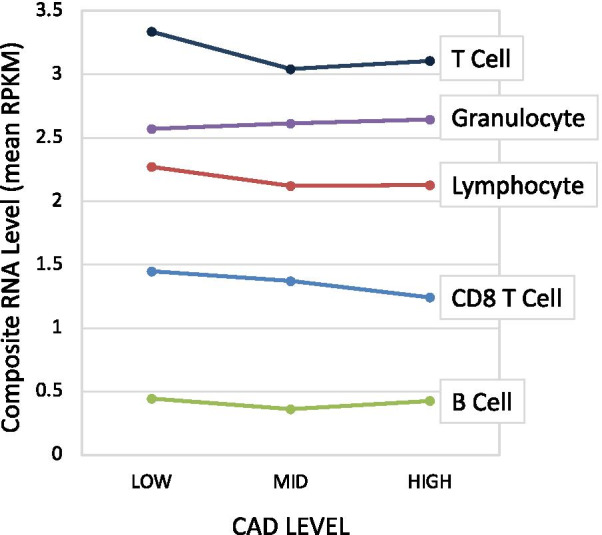
Expression of cell-type specific transcripts as a function of CAD status. Transcripts with relative specificity toward particular blood cell subsets was curated from published studies. The expression level (RPKM) of those transcripts (10–15 per cell type) in the RNAseq data was calculated and averaged for each cell type. The average expression was calculated for patients in 3 groups of CAD severity, LOW (n = 48), MID (n = 28), or HIGH (n = 20)

To explore a potential cell type hypothesis more directly, published microarray analysis of purified human blood subsets have identified cell type-specific mRNAs [56], which were cross-referenced to the current RNAseq transcriptome, and used to build a composite index of ~ 15 to 20 mRNAs relatively unique to each subtype. As shown in Fig. 5, a composite index of RNA expression levels shows a trend toward lower expression of lymphocyte markers in patients with MID to HIGH CAD. This trend is maintained in T-cells, and specifically in CD8 + T-cells, but is not observed in B-cell or granulocyte-related transcripts.

### TRACs do not appear to be markers of circulating progenitor cells

There is a substantial literature [57], summarized in Additional file 2: Table S4, that consistently reports reductions in circulating progenitor cell (CPC) populations in patients with stable CAD [58, 59], or preclinical atherosclerosis [60]. The major cardiovascular risk factors are associated with reduced numbers and activity of CPC [61]. Conversely, circulating endothelial progenitor cells (EPC) are increased in acute MI cases [62]. However, it is unlikely that a decrease in EPC numbers, which are rare (< 1%), could cause the substantial shift in RNA levels, detected in whole blood. Nonetheless, the RNAseq data was analyzed for changes in recognized markers of EPC and CPC, such as CD34, cKit, PROM1/AC133, and KDR, and the RNA levels are shown in Additional file 1: Fig. S4. There was no systematic change detectable: CD34 and cKit were slightly elevated in MID + CAD, while KDR and AC133 were decreased by comparable amounts.

### The expression of consensus Treg markers by CAD level

**Fig. 6:**
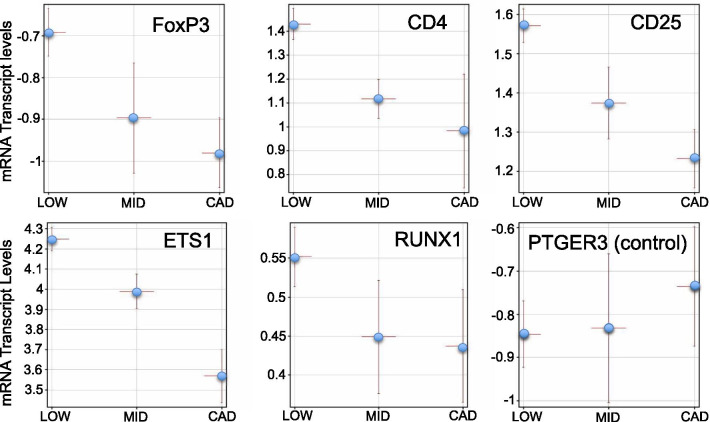
RNA levels of markers for Treg cells as a function of CAD level. The expression levels (log_2_ RPKM) of 5 known Treg markers (FoxP3, CD4, CD25, ETS1, Runx1) and 1 control (PTGER3) is plotted for 3 groups of patients with LOW (≤ 20% stenosis, n = 48), MID (21–69% stenosis, n = 28), or high CAD (≥ 70% stenosis, n = 20). Points are mean per group with bars ± s.e.m

A second potential explanation for TRACs as markers of a specific cell type is that there are known reductions in the Treg subset of lymphocytes in atherosclerosis [63]. An extensive literature documents reduced Treg abundance, and a relative imbalance in Treg vs T effector (Teff) cells in patients with CAD (summarized in Additional file 2: Table S5) [63, 64]. To test for the potential changes in Treg, the mRNA levels of known Treg markers was analyzed in the CAD groups. As shown in Fig. 6, five established markers of Treg cells, FoxP3, CD4, CD25, ETS1, and RUNX1, showed a stepwise decrease in mRNA expression from LOW, MID, to HIGH CAD. By comparison, the expression of an irrelevant marker, such as the prostaglandin E receptor 3 (PTGER3), does not show this CAD-related trend.

### TRACs correlate with FoxP3 and other Treg markers

To further understand whether the TRACs are related to Treg cell changes, the expression levels of FoxP3 were correlated with 24 other known Treg markers or TRACs across all 96 patients (Additional file 2: Table S6). The strongest correlations of FoxP3 occurred with AHRR (0.72, TRAC), CD8A (0.53), PDCD1 (0.48), ICOSLG (0.41, TRAC), RUNX1 (0.36), and PSORS1C1 (0.35, TRAC) (all p < 0.001), while other known Treg markers, such as IKZF4/Eos (0.13, p > 0.2, TRAC), showed weaker correlations. For reference, 2 splice variants of ICOS are correlated at 0.87, and 2 variants of B3GAT1 are correlated at 0.64. Furthermore, the levels of ICOS and ICOS-LG mRNA in whole blood are reduced comparably to FoxP3 in both the MID and HIGH CAD groups (Additional file 1: Fig. S5). Thus, the expression of several of the TRACs (AHRR, ICOSLG, PSORS1C1), correlate with FoxP3 RNA levels in these patients to a degree similar to or better than other known Treg markers (RUNX1, IKZF4/Eos).

### Treg/Teff cell ratio relative to TRAC RNA expression

To determine whether a reduction in Treg cell counts in blood would be sufficient in magnitude to produce the observed changes in RNA levels, 8 publications that reported Treg percentages in normal and CAD patients, such as unstable angina or acute coronary syndrome (ACS), were reviewed, and the change in Treg percentage was computed (Additional file 2: Table S5). The average Treg abundance, typically defined as CD4^+^CD25^+^CD127^low^ by flow cytometry, was 4.7% in normal, but decreased to 3.2% in CAD or unstable angina (30.3% reduction). This reduction in Treg abundance would translate to a 1.43-fold difference in Treg RNA levels, assuming that these markers are relatively unique to Tregs. Thus, the 1.47-fold reduction in mRNA for the consensus FoxP3 marker, and the ~ 1.7-fold reduction in the TRACs, is quite consistent with the reported reduction in Treg cell numbers in CAD.

### TRACs and Treg markers are sensitive to RNA stabilization procedures

Given that the Treg imbalance data has been reported from multiple labs worldwide, it is curious that changes in established Treg markers have not been reported in any prior publications using expression profiling of blood from patients with stable CAD. One possible explanation is that RNAseq is potentially much more sensitive than microarray methods, allowing these low abundance messages to be detected more accurately. A second consideration is that, to our knowledge, all prior CAD microarray studies were conducted using RNA stabilized and isolated from Paxgene preservative tubes, while the present studies employed Tempus preservative tubes. In the current studies, Tempus tubes were selected due to studies in our lab, and others, showing a ~ 10 to 20% better yield of RNA at 20% lower cost and 40% less time [65, 66]. Based on prior studies demonstrating quite marked changes in gene expression profiles based on the RNA stabilizer [20, 65, 66], the effect of the RNA stabilizer was examined for its impact on TRACs versus neutrophil transcripts (DEFA3) and other selected markers (IL12A, SELL, SOD2, TBA1, ACTB). As shown in Additional file 1: Fig. S6, blood from the same normal subjects at the same time, but collected into two different collection/stabilizer tubes, showed marked differences in the levels of mRNAs measured by droplet digital PCR (ddPCR). Several of the TRACs, such DGKA, DLG1, ICOSLG, and TCF3, were detected ~ 4 to sixfold more efficiently in blood RNA isolated from Tempus versus Paxgene tubes. Chemically, the Paxgene tubes are based on a cationic detergent that creates micellar-like structures that protect RNA, while Tempus uses the strong chaotrophic effects of guanidine-based salts to denature RNAses and dissolve RNA/protein complexes. Thus, the Tempus/chaotrophic approach appears to isolate Treg-related mRNAs better than the Paxgene/detergent approach.

### Analysis of TRACs in an independent validation cohort

Simple linear classification models built in the Discovery cohort (Fig. 4) and then applied to the RNAseq values obtained from 80 patients in the Validation Cohort did not perform much better than random in the validation set. However, it was quickly noted that the Discovery RNAseq had been aligned to the GRCh37/hg19 human genome, while the Validation set, aligned at a later date, used the GRCh38/hg38 reference genome. Thus, the entire Discovery RNAseq database was realigned to the hg38 genome and then reanalyzed for DEGs to build a classification model. As a quick test of the stability of a predictive model, in the hg38-aligned Discovery dataset, strict filtering for > twofold change at p < 0.001 identified 27 transcripts of which 23 (85%) were expressed at a lower level in the CAD group. A PLSD model built on those 27 transcripts was 95.5% accurate in LOW, 91.9% accurate in MID+, for 93.3% overall accuracy. However, those same transcripts were less predictive in the Validation dataset, but still informative, showing 78.4% accuracy for low, 62.8% for MID+, with 70% overall accuracy. Thus, the hg19 vs hg38 alignments play a significant role in the stability of the TRAC signal, but the discriminant ability of PLSD models remains imperfect between cohorts. To understand this discrepancy, the DEGs identified by each cohort were analyzed.

### Correlation between Discovery and Validation expression levels for TRACs

Using the list of 599 DEG transcripts identified in the Validation set, it was determined that their expression levels in the Discovery set were highly correlated for both the low (r = 0.96), and Mid + (r = 0.98) CAD groups. Thus, quantitation of the transcript levels in the 2 cohorts was very similar, at least at the group level (LOW vs MID +). Thus, the variation in the DEGs between the 2 cohorts is more likely attributable to variation at the patient to patient level, which could reflect the different demographics of the 2 groups.

### Identification of TRACs shared by the discovery and validation cohorts

Using the strictest filtering, exactly as applied to the Discovery set to obtain the 27 g predictors used above, the Validation dataset yielded 22 transcripts, but none were identical matches at the gene symbol level between cohorts. By relaxing the filtering criteria to create DEGs of about 350 unique and annotated transcripts in each cohort (p < 0.01, fc1.2), 16 exact matches were observed, which is 4.5 times greater than expected by chance (p < 8.7 × 10^−7^) (Additional file 2: Table S7). An additional 17 close matches were observed (ie. ELP3 vs ELP2), and 37 more matches that were close or identical to HG19 alignments of the Discovery cohort, for a total of 70 close or exact matches. Both the Discovery and Validation DEGs (92% decreased in CAD) shared a strong trend toward decreasing expression in the CAD group.

### Cell type analysis in reproducible TRACs

These transcripts common to both datasets were used to determine if any enrichment of a particular cell type was evident by comparing them to the precurated Blood Atlas RNAseq database. The results indicated the greatest similarity to T cells, with 12 exact or close matches (4.3-fold over-representation, p = 9.8 × 10^−6^, Fisher Exact test). Rather striking in this group of T cell-related transcripts, identified as significantly decreased in both cohorts, is FoxP1. While FoxP3 is considered a pivotal transcript in Treg development, FoxP1 is likewise a well-known and critical determinant of Treg maturation [67].

By comparison, the overlap of the shared DEG list with other cell types is less striking: B cell (1 exact, 4 close matches, 3.5 fold enrichment, p < 0.014), granulocytes (1 exact, 5 close matches), monocyte/macrophage (1 exact, 4 close matches), natural killer (NK) cells (1 close match), dendritic cells (3 exact, 4 close matches). Some transcripts, especially OSBPL10, were found as an exact match on multiple cell types, and thus do not truly inform the cell type analysis.

### Prevalence of transcripts associated with stress granules (SG)

In addition to the apparent similarity of TRACs with Treg markers, it was also noted that a disproportionate number of transcripts had a known association with stress granules (Additional file 2: Table S8). Stress granules are membrane-less granules that result from liquid to gel transitions under cellular stress, and contain RNAs that are being sequestered from translation during various stressors, such as nutrient stress. Fortunately, other groups have used relatively unbiased approaches, such as microarrays and RNAseq, to identify RNA transcripts retained in SG during stress [68]. Thus, this hypothesis was tested more formally by comparing the TRACs to known SG transcripts and determining whether the overlap was greater than expected by chance.

**Fig. 7:**
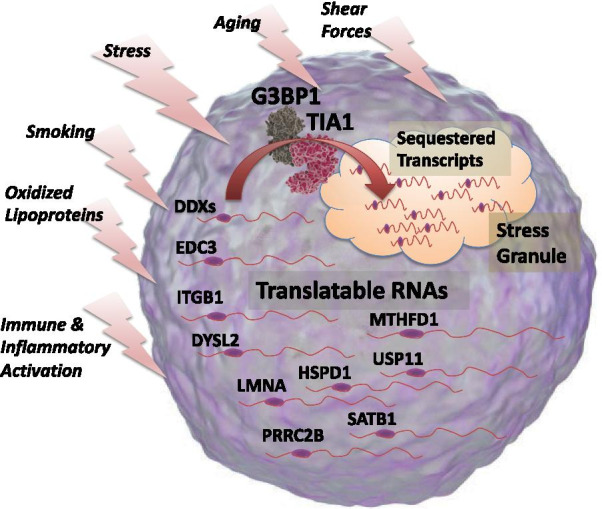
Schematic representation of stress granule-regulated transcripts. Analysis of the transcripts associated with CAD (TRACs) indicated an apparent enrichment for transcripts previously known to be associated with stress granules, which are membrane-less aggregates of proteins and RNA formed when cells are exposed to a variety of stressors, listed on the left. Under stress, these TRACs, of which 10 are shown here (DDX, EDC3, etc.), translocate from active, translatable forms in the cytosolic machinery, to sequestered, inactive forms in the stress granule. Molecular images courtesy of www.somersault1824.com under a Creative Commons license

In the initial hg19 TRAC list (198 transcripts, Additional file 2: Table S1) there was noticeable similarity to previously published lists of SG transcripts [68]. For instance, of the 198 TRACs, 34 were near or exact matches to known SG transcripts, reflecting a fivefold overrepresentation (p = 9.5 × 10^−15^). This association held strong when the TRACs common to both studies were analyzed for their similarity to a previously curated list of 723 known SG transcripts, whereby there was a 25-fold enrichment for SG transcripts (p = 4.04 × 10^−39^). A summary of these transcripts is shown in Additional file 2: Table S8, and 10 transcripts are depicted graphically in Fig. 7.

The stress granule-related RNAs include dead box proteins (DDXs, including DDX46, DDX51, DDX54), which are a family of RNA helicases that regulate RNA biogenesis, editing, folding, translation, and decay, as well as having critical antiviral activities [69]. Likewise, EDC3 is considered an important regulator of mRNA translation and decay [70], and interestingly, DDX proteins (i.e. DDX6) are known partners to EDC3 and mRNA decapping enzymes in the regulation of P-body assembly and function [71]. Of note is the Lamin A (LMNA) transcript, which is the target of germline mosaic mutations in Hutchinson-Guilford Progeria, a premature aging syndrome characterized by aggressive atherosclerosis and myocardial infarction in adolescents [72]. Also of interest, special AT-rich binding protein (SATB1) is a key chromatin protein that is a well-established modulator of T cell progenitor maturation [73]. Notably, SATB1, along with IKZF4/Eos, IRF4, and GATA1, are considered a Treg ‘locking’ genes [74].

### Potential involvement of cilia/immune synapse transcripts

During manual curation of the DEG transcripts from both cohorts, there was an apparent overrepresentation of transcripts related to cilia, synapses, and adhesion: functions not normally associated with circulating cells. A representative list of 11 such transcripts derived from the DEGs common to both cohorts is shown in Additional file 2: Table S9. An excellent example is Bardet-Biedl Syndrome 2 (BBS2) which is a heritable cause of an autosomal recessive syndrome characterized by central obesity, rod-cone dystrophy, renal and vascular abnormalities that emanate from a central defect in cilia assembly and synaptic function [75]. Related transcripts that appeared in only one of the cohorts includes dystonin (DST), which likewise affects the ciliary connections in the ear, causing congenital deafness, but has also been associated by GWAS with CAD [76]. Other DEGs common to both cohorts include copine 3 or 6 (CPNE3/6) which are components of the ciliary body, and affects neural plasticity, but coincidentally, reduced CPNE3 expression is associated with the risk of acute MI and stable CAD [77]. A potential connection between these cilia/synaptic transcripts and the Treg changes in atherosclerosis is that the maturation of Tregs likely depends on proper immune synapse formation in maturing T cells [78].

### Comparison of TRACs to prior microarray-based biomarker panels

Other published works have identified transcripts with predictive value for CAD based on Affymetrix array technology and PaxGene blood RNA preservation tubes [12]. For comparison purposes, these published transcripts were matched by gene symbol to RNAseq transcripts, identifying 17 transcripts in the current RNAseq dataset. The expression levels of these array-based markers were overall much higher than TRACs, but surprisingly, the RNA levels did not differ between LOW and MID + (average log_2_ RPKM = 3.26 vs 3.31, p = 0.94) (Additional file 2: Table S10). These 17 transcripts were used to build a classification model that yielded only 36.7% accuracy (LOW 45.1%, MID 10%, CAD 37.5%, w/ 33% = random). However, in fairness to the prior CAD biomarkers, it is difficult to extrapolate their weighting algorithm to the RNAseq data, and that might improve the prediction model.

## Discussion

The analysis of the RNA transcriptome in relation to angiographically confirmed CAD offers several major advantages in both our basic science understanding of CAD and in clinical medicine. First, if blood biomarkers can be identified, it might be possible to reduce invasive testing, such as cardiac catheterization, as well as more judiciously use imaging resources, such as CT and MR angiography. Secondly, it would be possible to improve diagnosis of CAD in rural areas worldwide, where invasive or advanced imaging methods are unavailable. Finally, the proposed biomarkers potentially can serve both as therapeutic targets and markers to monitor the appropriateness and efficacy of new or existing therapies, such as statins or PCSK9 inhibitors. For instance, Treg numbers have been shown to be responsive to statin therapy, and so it might be possible to use TRACs to monitor statin therapies.

The connection between the immune system and atherosclerosis is extensively documented. Blood components, especially monocytes/macrophages [79], neutrophils, lymphocytes [80], and platelets mechanistically contribute to the development of CAD [81]. Recently, the microanatomy of the human carotid atherosclerotic lesion has been analyzed by single-cell transcriptomics, revealing at least 14 subtypes of cells, including several T cell subsets [82]. The present results are consistent with the extensive evidence that CAD is associated with changes in the Treg/Teff ratio, lipid imbalances, inflammation, microbiome changes, and autoimmunity in atherosclerosis [83]. There is a large and fairly consistent literature demonstrating changes in the Treg/Teff ratio in patients with CAD [84–88], and the observed cellular changes would be consistent in both direction and magnitude with the detected changes in mRNA expression in the present studies (Additional file 2: Table S5). One interpretation of the beneficial effects of statins is that in addition to lowering LDL cholesterol, statins can induce FoxP3 + Treg cells, via modulation of TGF-ß signaling [89, 90]. Beyond the reproducible clinical correlations, experimental manipulation of Treg levels in mouse models of atherosclerosis suggests a potentially causal relationship [91]. Furthermore, it has been suggested that a Treg-oriented immunomodulatory approach may offer therapeutic potential for atherosclerosis [92, 93].

The relationship between Treg dysfunction and atherosclerosis is further observed through the well-known incidence of atherosclerosis in various autoimmune diseases, most notably in the case of systemic lupus erythematosus (SLE) [94]. While the relationship between Tregs and SLE is complex, there is a general consensus that deficient Treg activity is one element of SLE [95], and thus, might also be a component of SLE-associated atherosclerosis [96]. Likewise, psoriasis and psoriatic arthritis, which are associated with Treg imbalances, have well-established associations with atherosclerosis [97–99]. The immune-CAD connection is seen quite clearly by an apparently causal relationship in immune-mediated transplant arteriosclerosis [100]. Further evidence for the immune-CAD connection derives from the proven efficacy of rapamycin and related compounds, which are antibiotics/immunosuppressants, to block coronary artery restenosis. Rapamycin is known to increase Treg numbers and function at clinically relevant levels [101]. Recent findings provide fairly direct evidence that the cytokine responsiveness of T cell subsets is a better predictor of CAD than CRP [102]. Important recent studies indicate that Tregs license the pro-resolving abilities of plaque-resident macrophages in order to facilitate plaque regression [103].

**Fig. 8:**
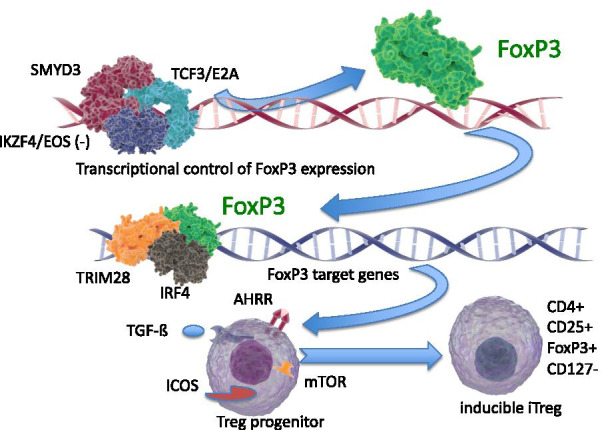
Schematic representation of Treg-related TRACs identified by RNAseq. The control of FoxP3 mRNA and protein expression is known to be controlled by many factors, including promoter methylation, as well as transcriptional regulation by SMYD3, TCF3/E2A, and IKZF4/Eos. FoxP3, in turn, is itself a transcriptional regulator, in association with cofactors such as TRIM28, IRF4, and others. The FoxP3-sensitive target genes, and other regulators such as AHRR, ICOS, TGF-ß, and mTOR, are then intrinsic components of the transition of Treg progenitor cells to functional Tregs. Molecular images courtesy of www.somersault1824.com under a Creative Commons license

Many of the TRACs identified herein have known relationships with Treg function, as shown schematically in Fig. 8. Foremost, the FoxP3 transcription factor is considered the definitive marker for the Treg subset and is thought to transcriptionally regulate a set of transcripts involved in Treg function. FoxP3 is itself epigenetically controlled by promoter demethylation and transcription factors, such as SMYD3, IKZF4/Eos, and TCF3/E2A, to allow stable expression in Tregs. Once transcribed and translated, FoxP3 regulates Treg-specific transcription via known promoter binding sites [55] and by interaction with a number of co-regulators, including RUNX1 [104], TRIM28, and IRF4 [105], which, along with SMYD3, IKZF4/Eos and TCF3, were identified as TRACs in the present studies. Other studies indicate that two isoforms of diacylglyceral kinase (DGKA, DGKZ) have been implicated in T-cell anergy [106], and DGKZ has been implicated in the generation of natural Tregs via modulation of the NFkB signaling through c-rel [107].

The ICOS/ICOSLG system is potentially important because athero-prone LDLR(-) mice transplanted with ICOS-deficient marrow develop more severe atherosclerosis [108]. Treg can be either ICOS+ or ICOS− [109], and typically ICOSLG is considered as a marker of a dendritic cell or innate lymphoid cell type 2 (ILC2), whereby ICOS on the Treg would engage ICOSLG on the ILC2s [110]. Flow cytometry analysis of Tregs in MI patients showed a subset of ICOS+ type to be preferentially affected [111]. Thus, the decrease in ICOSLG suggests that there may be some type of coordinate decrease in both Treg and ILC2 numbers or function.

The current studies differ from prior microarray-based analysis of CAD in several important ways. The current studies used a broader definition of CAD that would include earlier and more diffuse, but less occlusive disease. This definition also provided a better balance between the sizes of affected and unaffected groups. Further, prior studies used Paxgene blood RNA preservative, which is known to produce a very different RNA profile, while the present Tempus system was more sensitive for Treg markers. Likewise, RNAseq appears to be important for detecting changes in Treg activity and provides numerous quantitative and qualitative advantages over microarrays.

There are certain limitations in the present studies. First, the TRAC signature could be related to unidentified risk factors or drug treatments that differ between groups. While it is difficult to completely rule it out, based on the collected variables, we cannot identify a clinical covariate that would differ sufficiently to create this effect. Second, it is possible that the TRACs would detect disease in arteries other than the coronaries, but this would still have tremendous diagnostic value. A third limitation is that the clinical endpoint of an invasive coronary angiography (ICA) is excellent, but still imperfect at detecting coronary disease. Up to 75% of symptomatic patients that appear to have normal arteries by ICA can be shown by CT angiography to have significant atherosclerosis that does not occlude the artery [112]. The TRAC test would likely report these patients as positive, while they would be scored as angiographically negative for CAD by ICA. Future validation studies will need to incorporate CT angiography as an additional endpoint to avoid these ambiguous ‘false positives’ on blood-based tests. Likewise, TRAC-positive patients that are angiographically negative, could be in the early stages of the disease process, but that could be addressed only by a long-term follow-up study.

The present studies suggest several important directions for future investigation. Bioinformatically, it would be valuable to analyze the co-expression network of the transcripts, and analyze any RNA editing, differential splicing, and allele usage that might be occurring in CAD. The identification of RNA biomarkers that are associated with CAD has the potential to help dissect the mechanisms of atherosclerotic initiation and progression. It is likely worthwhile to further investigate the regulation of these transcripts by stress granules, as one component of immune dysfunction in coronary disease. Further, a potential connection between stress and the function of the immune synapse could elucidate specific mechanisms of disease, and targets for therapy, or prevention. Through high-throughput screening, dozens of FDA-approved compounds that stimulate Treg generation have already been identified [113]. Further refinement in the quantitation of these RNA biomarkers could lead to blood-based diagnostics for CAD, that would be a valuable addition to the diagnostic toolkit.

A long-term goal is to identify TRACs that may be predictive of CAD in asymptomatic, but ‘at risk’ individuals, especially middle age patients with one or more known risk factors [114]. Of the more than a million heart attacks per year in the US, approximately 50% of cases had no overt warning signs, and 50% of first heart attacks are fatal. Thus, an ‘early warning sign’ from blood-based RNA profiling could allow the patient to be referred for minimally-invasive diagnostics, such as stress tests, CT calcium scores, or MR/CT angiography, and thus hopefully reducing the incidence of heart attacks and strokes.

## Conclusions

Transcriptome-wide profiling of whole blood RNA from patients with CAD identifies a pattern of changes that parallels known defects in the number and function of the regulatory T cell subset. The RNA pattern defines a risk that is independent of other known clinical risks, and thus could add value to future risk stratification models. Simple linear classification models using only seven transcripts provides surprisingly strong prediction of CAD status as determined by invasive coronary angiography. The RNA changes are consistent with stress-related changes in the immune synapse, which may help to define the precise cellular mechanisms of atherosclerotic lesion formation.

## Supplementary Information

**Additional file 1.** Six supplementary figures related to the RNAseq analysis of CAD.

**Additional file 2.** Ten supplementary tables providing transcript details for the RNAseq analysis of CAD.

## Data Availability

The expression-level data (raw RPKM) is deposited in the Gene Expression Omnibus (GEO) at the accession # GSE180083. The raw sequence files from this study will be provided to qualified investigators that can insure compliance with appropriate IRB and HIPPAA regulations for any future data usage, by contacting the corresponding author at mcc@gwu.edu. The human genome files for alignment were obtained from UCSC at this link for HG19 (https://hgdownload.soe.ucsc.edu/goldenPath/hg19/bigZips/) and HG38 (https://hgdownload.soe.ucsc.edu/goldenPath/hg38/bigZips/).

## Abbreviations

CAD: Coronary artery disease
CBC: Complete blood count
CPC: Circulating progenitor cells
ddPCR: Droplet digital PCR
DEGs: Differentially expressed genes
GERD: Gastroesophageal reflux disease
ICA: Invasive coronary angiography
PLSD: Partial least squares discriminant model
RIN: RNA integrity number
RNAseq: RNA sequencing
rRNA: Ribosomal RNA
RPKM: Reads per kilobase of exon per million mapped total reads
STEMI: ST segment elevation myocardial infarction
SG: Stress granules
SLE: Systemic lupus erythematosus
TRACs: Transcripts associated with CAD
Treg: Regulatory T cell
