# Supplementary material for: RNA sequencing of blood in coronary artery disease: involvement of regulatory T cell imbalance
Source: BMC Med Genomics. 2021 Sep 3;14:216. doi: 10.1186/s12920-021-01062-2 (PMC8414682; doi:10.1186/s12920-021-01062-2)
Supplement: Supplementary file 1 — Additional file 1. Six supplementary figures related to the RNAseq analysis of CAD. [file 12920_2021_1062_MOESM1_ESM.pdf]

RNA Yield vs Lymphocyte Count

CathDx dataset

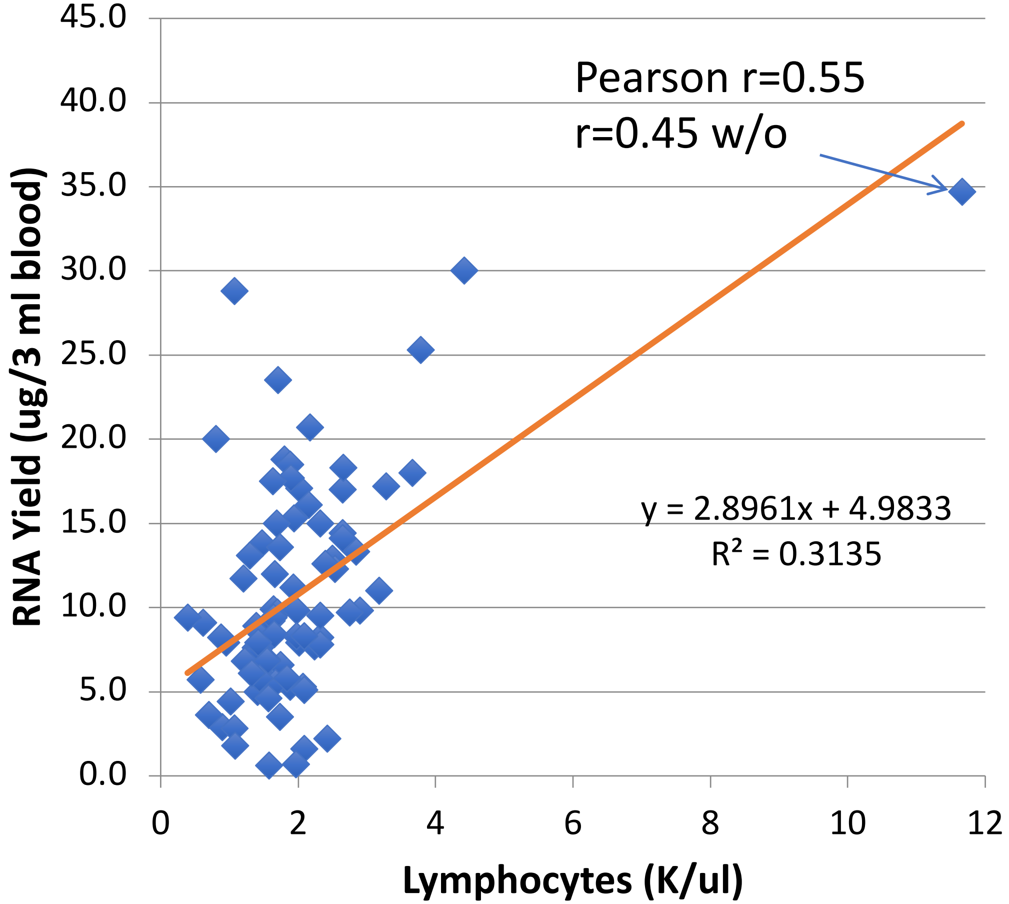

Supplementary Figure 1. RNA yield versus Lymphocyte count. The relationship between RNA yield (µg/tube blood sample), determined by OD 260/280, and lymphocyte count (K/ul), determined by automated complete blood count (CBC), is plotted for the 96 patients included in the RNAseq analysis. A trendline (red) is plotted with the Pearson r correlation, the linear regression, and the R^2. The correlation is calculated with (0.55) and without (0.45) without the outlier patient in the upper right.

Covariate analysis

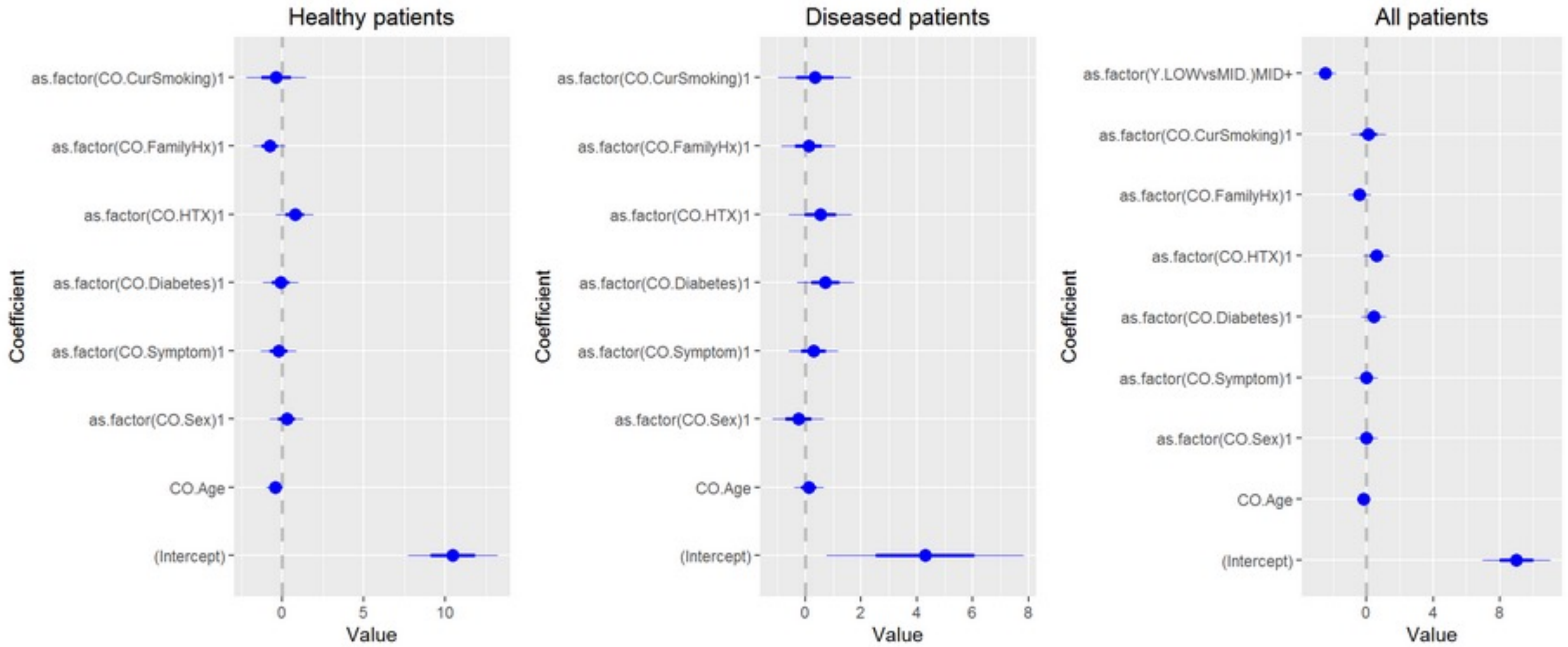

Supplementary Figure 2. Covariate analysis of clinical parameters with the TRAC score of RNA expression in blood. Clinical parameters, as summarized in Table 1, were used to determine whether the TRAC score was sensitive to pre-existing clinical values that have been associated with CAD risk. The relative contributions of current smoking, family history, hypertension (HTX), diabetes, symptoms (typical/atypical), gender (sex), and age were evaluated in the healthy patients, CAD patients, or combined.

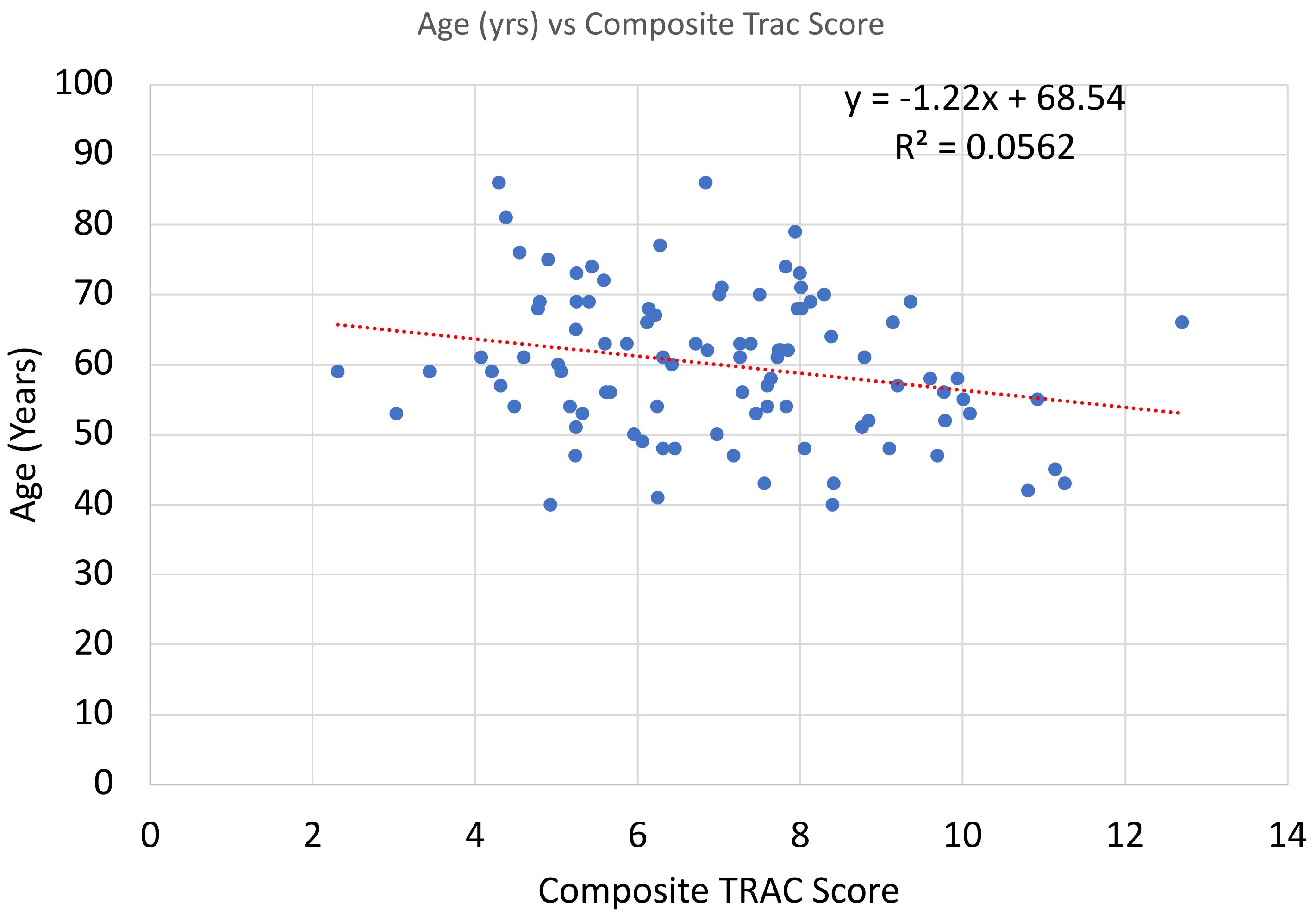

Supplementary Figure 3. Monovariate analysis of the relationship between age and TRAC score. Age was a weak covariate for the TRAC score and so the monovariate relationship between Age (Y axis) and TRAC score (X axis) is plotted with a linear trendline and R^2 in the 96 patients analysed by RNAseq.

EPC/CPC Markers vs CAD level

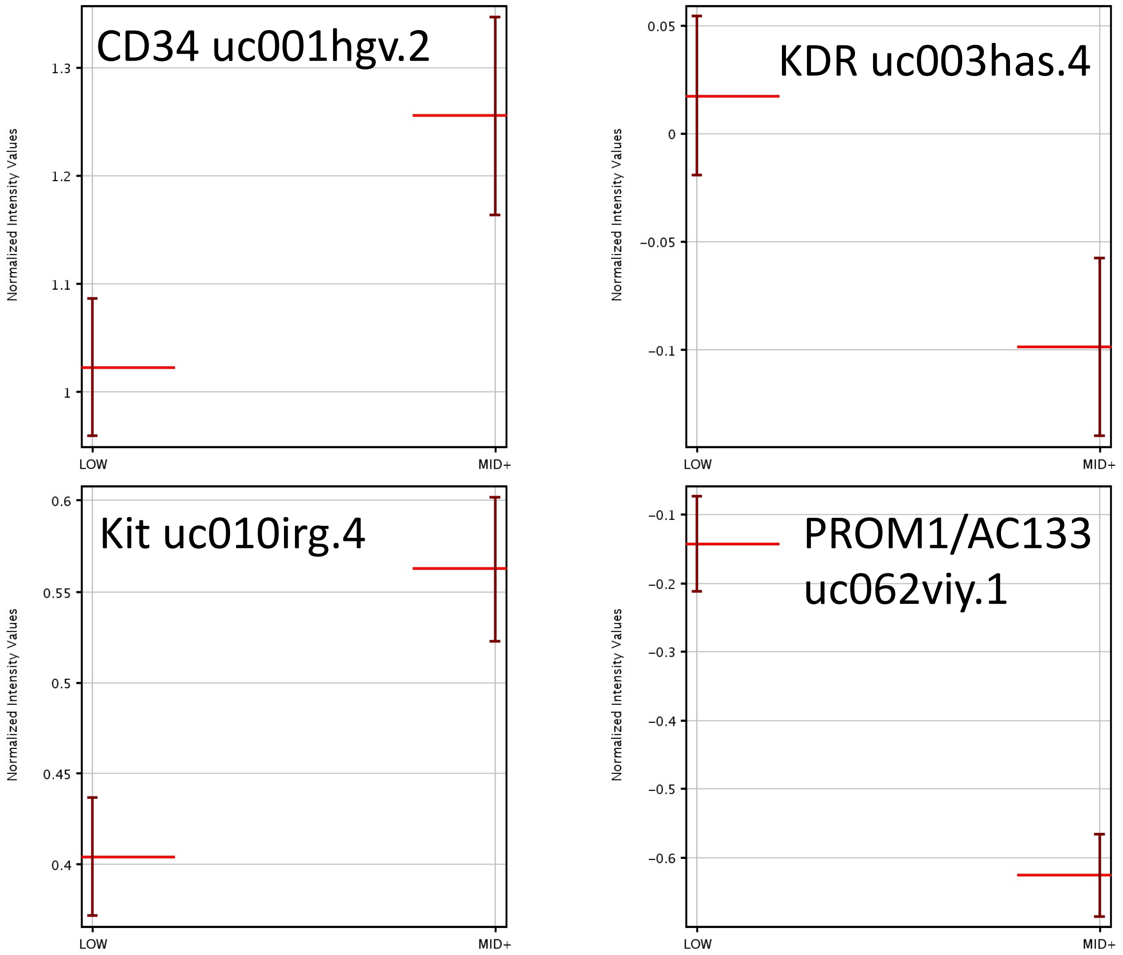

Supplementary Figure 4. RNA levels of markers related to endothelial progenitor cells and/or circulating progenitor cells (EPC/CPC). The RNA expression levels (log_2 RPKM) of 4 markers of EPC/CPC, CD34, KDR, Kit, and AC133 are shown with their UCSD IDs. Expression levels are plotted (Y axis, mean with SEM bars) for patients in the LOW (n=48) versus MID+ (n=48) CAD groups.

ICOS/ICOS-LG by CAD Status

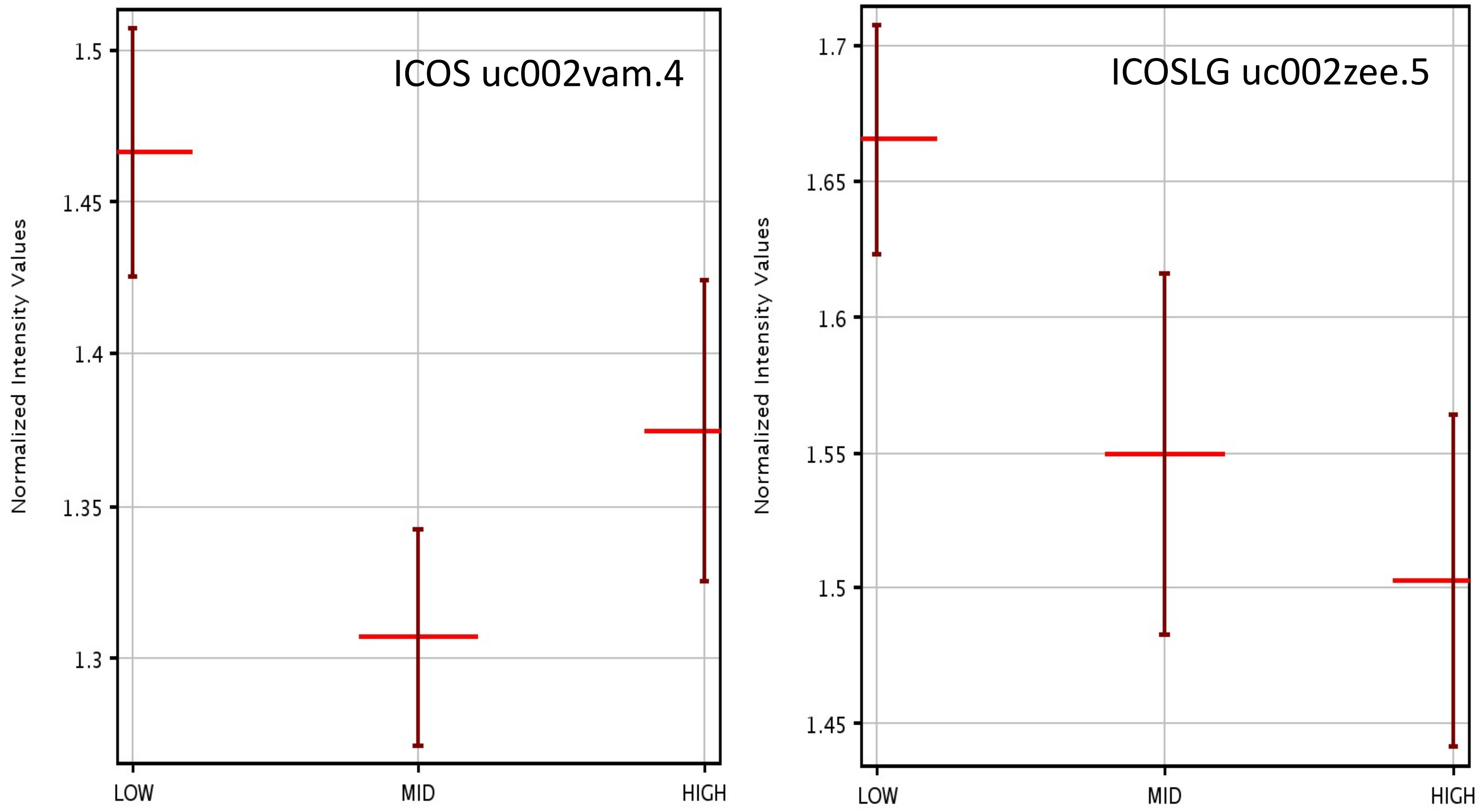

Supplementary Figure 5. Transcripts levels for ICOS and ICOS-LG by level of CAD. The expression levels (log_2 RPKM) of ICOS (UCSC Id uc002vam.4) and ICOS-ligand (ICOS-LG, uc002zee.5) are plotted as a function of LOW, MID, and HIGH CAD levels as shown in Figure 6. The points are mean + s.e.m. for each group.

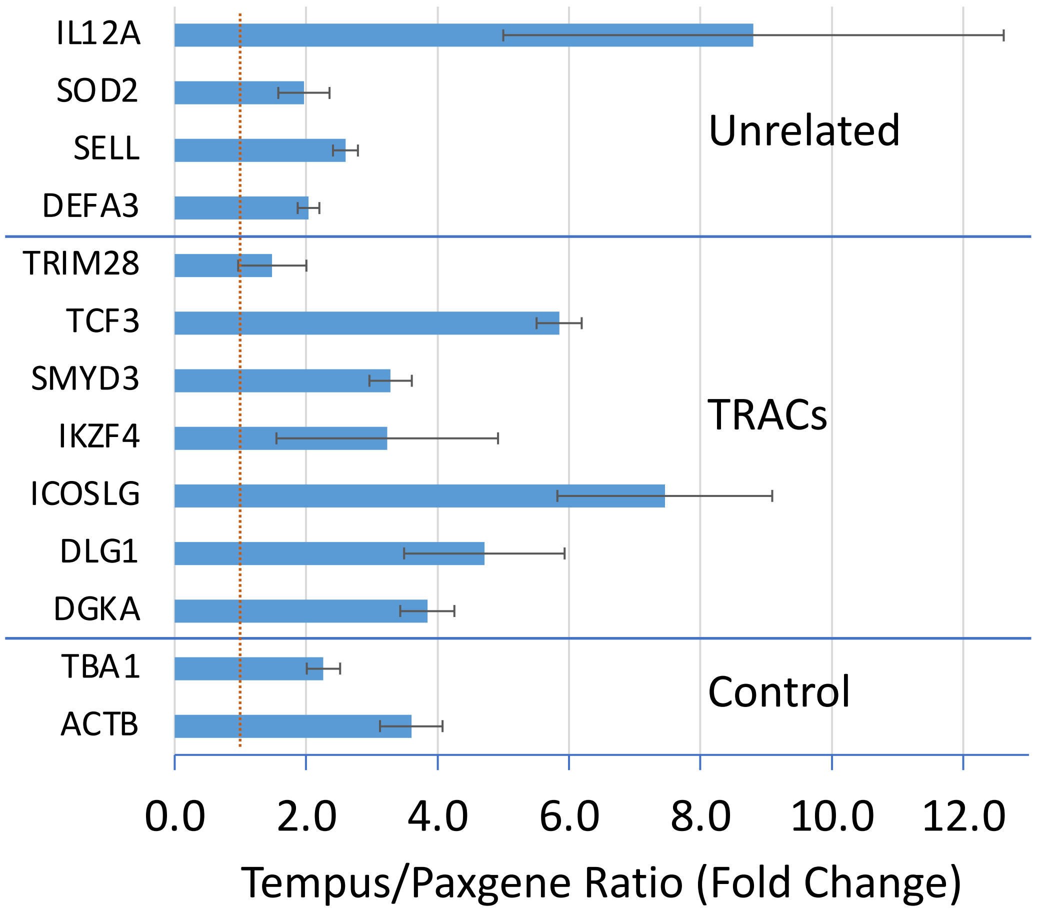

Supplementary Figure 6. The effect of RNA collection/stabilizer solution on expression of TRAC and unrelated transcripts. Whole blood RNA was prepared by two different methods of collection and stabilization from the same donors (n=3). RNA was prepared by PaxGene, which is principally based on a cationic detergent, cetylpyridinium chloride (CPC), or Tempus, which is based on the strong chaotrophic effects of guanidine salts. Identical quantities of DNAse-treated RNA were reverse transcribed using SuperScript III and then quantitatively amplified using droplet-digital PCR (ddPCR, BioRad). Levels shown are the ratio of transcript abundance in Tempus vs Paxgene, based on absolute quantities calculated from a Poisson distribution of ~15K droplets (±sem).
