# Supplementary material for: RNA sequencing of blood in coronary artery disease: involvement of regulatory T cell imbalance
Source: BMC Med Genomics. 2021 Sep 3;14:216. doi: 10.1186/s12920-021-01062-2 (PMC8414682; doi:10.1186/s12920-021-01062-2)
Supplement: Supplementary file 2 — Additional file 2. Ten supplementary tables providing transcript details for the RNAseq analysis of CAD. [file 12920_2021_1062_MOESM2_ESM.pdf]

**Supplementary Table 1.**

#Entitylist : RPKM>0.01 70%

#Interpretation : LMH 96 NOTE: this list has all 96 curated samples

#Experiment: CDX96v1 198 total transcripts

#corrected p-value cut-off:0.001 169 unique non-redundant transcripts

#Selected Test : Oneway ANOVA

#p-value computation: Asymptotic

#Multiple Testing Correction: No Correction

KEY:
- association with CAD or cardiovascular parameters
- potential involvement in Treg function or differentiation
- centrosome related

| | | | | | | Log2 Normalized | | | | | |
|---|---|---|---|---|---|---|---|---|---|---|---|
| **GeneName** | **p** | **FC ([HIGH] '** | **Regulat** | **FC ([MID] vs** | **Regula** | **[HIGH]** | **[LOW]** | **[MID]** | **Symbol** | **Description** | **RefSeq** |
| uc061drv.1 | 2.93E-04 | 1.01 | up | -4.97 | down | -1.24 | -1.25 | -3.56 | A1BG | alpha-1-B glycoprotein (A1BG), mRNA. (from RefSec | NM_130786 |
| uc057ilc.1 | 5.27E-04 | 1.86 | up | -5.43 | down | -1.85 | -2.75 | -5.19 | ABCA4 | ATP-binding cassette, sub-family A (ABC1), member | NM_000350 |
| uc003wjo.3 | 4.56E-04 | -1.25 | down | -1.29 | down | 0.77 | 1.09 | 0.72 | ABCF2 | ATP-binding cassette, sub-family F (GCN20), membe | NM_005692 |
| uc064bvu.1 | 9.54E-04 | -1.16 | down | -3.83 | down | 0.03 | 0.24 | -1.70 | AC099342.1 | uncharacterized LOC101927769, long non-coding RI | NR_110117 |
| uc059xhj.1 | 3.89E-04 | -1.24 | down | -1.75 | down | 1.44 | 1.74 | 0.94 | ADAT1 | adenosine deaminase, tRNA-specific 1 (ADAT1), trar | NM_012091 |
| uc021xqj.2 | 3.37E-04 | -1.74 | down | -1.70 | down | -2.79 | -2.00 | -2.76 | ADH7 | alcohol dehydrogenase 7 (class IV), mu or sigma pol | NM_0011665 |
| uc063bvy.1 | 4.06E-07 | 1.50 | up | -11.42 | down | -2.18 | -2.77 | -6.29 | AHRR | aryl-hydrocarbon receptor repressor (AHRR), transc | NM_0012424 |
| uc063bvz.1 | 9.62E-05 | -1.25 | down | -7.08 | down | -0.12 | 0.20 | -2.62 | AHRR | aryl-hydrocarbon receptor repressor (AHRR), transc | NM_0012424 |
| uc062jar.1 | 3.44E-04 | -1.28 | down | -1.40 | down | -1.82 | -1.46 | -1.95 | ALS2CL | ALS2 C-terminal like | NR_033815 |
| uc064mjf.1 | 8.03E-04 | -1.24 | down | -1.42 | down | -1.50 | -1.18 | -1.69 | AP3M2 | adaptor-related protein complex 3, mu 2 subunit (AP3 | NM_006803 |
| uc001mdb.4 | 7.45E-04 | -1.29 | down | -1.35 | down | -1.09 | -0.72 | -1.15 | APBB1 | amyloid beta (A4) precursor protein-binding, family B, | NM_001164 |
| uc001mdc.3 | 4.90E-04 | -1.29 | down | -1.39 | down | -0.93 | -0.56 | -1.04 | APBB1 | amyloid beta (A4) precursor protein-binding, family B, | NM_145689 |
| uc057yjv.1 | 3.32E-04 | -1.32 | down | -1.39 | down | -1.00 | -0.60 | -1.07 | APBB1 | amyloid beta (A4) precursor protein-binding, family B, | NM_001164 |
| uc057yjz.1 | 9.37E-04 | -1.29 | down | -1.34 | down | -1.11 | -0.74 | -1.16 | APBB1 | amyloid beta (A4) precursor protein-binding, family B, | NM_001164 |
| uc057ykd.1 | 1.32E-04 | -1.57 | down | -1.35 | down | -1.81 | -1.16 | -1.59 | APBB1 | amyloid beta (A4) precursor protein-binding, family B, | NM_0012573 |
| uc062dsw.1 | 6.45E-04 | 1.01 | up | -3.95 | down | -2.77 | -2.78 | -4.76 | APOL4 | apolipoprotein L, 4 (APOL4), transcript variant a, mRI | NM_030643 |
| uc064snu.1 | 5.81E-04 | -1.64 | down | -5.31 | down | -3.71 | -3.00 | -5.41 | APTX | aprataxin | NR_036576 |
| uc064soc.1 | 7.40E-04 | 1.10 | up | -4.11 | down | -0.82 | -0.96 | -3.00 | APTX | aprataxin | NR_036577 |
| uc065ahz.1 | 7.23E-04 | -1.34 | down | -1.45 | down | -0.51 | -0.09 | -0.62 | ARMCX6 | armadillo repeat containing, X-linked 6 (ARMCX6), tra | NM_0011847 |
| uc064pgy.1 | 3.53E-04 | -3.10 | down | -1.70 | down | -2.19 | -0.56 | -1.33 | AZIN1-AS1 | AZIN1 antisense RNA 1 | NR_126339 |
| uc059ulu.1 | 5.07E-04 | -1.57 | down | -2.22 | down | -0.71 | -0.06 | -1.21 | BBS2 | Bardet-Biedl syndrome 2 (BBS2), mRNA. (from RefS | NM_031885 |
| uc061wwa.1 | 6.36E-04 | -1.44 | down | -1.39 | down | -0.29 | 0.24 | -0.23 | BLCAP | bladder cancer associated protein (BLCAP), transcri | NM_0011678 |
| uc058bon.1 | 8.72E-04 | -1.47 | down | -1.32 | down | 1.12 | 1.68 | 1.28 | C11orf31 | chromosome 11 open reading frame 31 (C11orf31), r | NM_170746 |
| uc059tos.1 | 3.30E-04 | -1.19 | down | -1.47 | down | -1.01 | -0.77 | -1.33 | C16orf58 | chromosome 16 open reading frame 58 (C16orf58), r | NM_022744 |
| uc063kor.1 | 2.91E-04 | -1.42 | down | -6.18 | down | -2.22 | -1.71 | -4.34 | C5ORF60 | chromosome 5 open reading frame 60 | NR_131066 |
| uc063nqr.1 | 3.79E-04 | 1.29 | up | -3.35 | down | -5.67 | -6.04 | -7.78 | C6orf10, | chromosome 6 open reading frame 10 (C6orf10), trar | NM_0012864 |
| uc064rtg.1 | 4.78E-04 | -1.48 | down | -2.36 | down | -3.80 | -3.23 | -4.47 | CBWD1 | COBW domain containing 1 (CBWD1), transcript vari | NM_018491 |
| uc064bwy.1 | 4.66E-04 | -1.67 | down | -1.50 | down | -1.09 | -0.36 | -0.95 | CDCA7L | cell division cycle associated 7-like (CDCA7L), trans | NM_0011273 |
| uc059xrj.1 | 1.45E-04 | -2.97 | down | -8.68 | down | -2.86 | -1.29 | -4.41 | CDH13 | cadherin 13 (CDH13), transcript variant 5, mRNA. (fr | NM_0012204 |
| uc061mig.1 | 6.28E-04 | -1.21 | down | -3.99 | down | -2.78 | -2.50 | -4.49 | CHST10 | carbohydrate sulfotransferase 10 (CHST10), mRNA. | NM_004854 |
| uc062hvi.1 | 4.91E-04 | -1.33 | down | -1.24 | down | -0.09 | 0.33 | 0.01 | CNOT10 | CCR4-NOT transcription complex subunit 10 | NR_046352 |
| uc064rqz.1 | 8.12E-04 | -1.46 | down | -1.57 | down | 0.09 | 0.64 | -0.01 | COMMD5 | COMM domain containing 5 (COMMD5), transcript v | NM_0010810 |

| | | | | | | | | | | | |
|---|---|---|---|---|---|---|---|---|---|---|---|
| uc057ugl.1 | 8.07E-04 | -1.22 | down | -1.81 | down | -0.50 | -0.20 | -1.06 | COMTD1 | catechol-O-methyltransferase domain containing 1 (( | NM_144589 |
| uc001tyn.4 | 6.14E-04 | -1.43 | down | -1.01 | down | 0.12 | 0.64 | 0.63 | COQ5 | coenzyme Q5 homolog, methyltransferase (S. cerevi | NM_032314 |
| uc062lwg.1 | 6.59E-04 | -1.37 | down | -1.47 | down | 1.17 | 1.63 | 1.07 | CPOX | coproporphyrinogen oxidase (CPOX), mRNA. (from I | NM_000097 |
| uc010rgn.2 | 7.67E-04 | -1.20 | down | -1.19 | down | 0.61 | 0.88 | 0.62 | CRY2 | cryptochrome circadian clock 2 (CRY2), transcript va | NM_021117 |
| uc058ato.1 | 7.22E-04 | -1.20 | down | -1.19 | down | 0.61 | 0.88 | 0.62 | CRY2 | cryptochrome circadian clock 2 (CRY2), transcript va | NM_021117 |
| uc057oir.1 | 8.16E-04 | -1.23 | down | -1.54 | down | -0.56 | -0.26 | -0.89 | CSRP1 | cysteine-rich protein | NM_0011935 |
| uc062ukk.1 | 9.07E-04 | -1.21 | down | -1.51 | down | 0.16 | 0.43 | -0.16 | CTBP1 | C-terminal binding protein 1 (CTBP1), transcript varia | NM_0010126 |
| uc059zqi.1 | 7.57E-04 | -1.56 | down | -1.90 | down | -0.89 | -0.25 | -1.17 | CTNS | cystinosin, lysosomal cystine transporter (CTNS), tra | NM_004937 |
| uc057jkc.1 | 6.37E-04 | -1.26 | down | -1.51 | down | -0.35 | -0.02 | -0.61 | CTTNBP2NL | CTTNBP2 N-terminal like (CTTNBP2NL), mRNA. (frc | NM_018704 |
| uc061otq.1 | 1.03E-04 | -1.47 | down | -1.41 | down | -0.17 | 0.38 | -0.11 | **CYTIP** | cytohesin 1 interacting protein (CYTIP), mRNA. (from | NM_004288 |
| uc058mlq.1 | 8.52E-04 | -1.44 | down | -2.37 | down | -1.71 | -1.19 | -2.43 | DDX11 | DEAD/H (Asp-Glu-Ala-Asp/His) box helicase 11 (DD. | NM_0012571 |
| uc060zfv.1 | 6.81E-04 | -1.12 | down | -1.47 | down | 0.64 | 0.81 | 0.26 | DEDD2 | death effector domain containing 2 | NR_073051 |
| uc021wlp.2 | 9.01E-05 | -1.52 | down | -1.48 | down | -0.18 | 0.43 | -0.14 | DGCR8 | DGCR8 microprocessor complex subunit (DGCR8), | NM_0011903 |
| uc058pdy.1 | 7.75E-04 | -1.36 | down | -1.44 | down | -0.26 | 0.18 | -0.34 | **DGKA** | diacylglycerol kinase, alpha 80kDa (DGKA), transcrip | NM_201554 |
| uc062jfe.1 | 3.85E-04 | -1.73 | down | -1.44 | down | -0.46 | 0.34 | -0.19 | DHX30 | DEAH (Asp-Glu-Ala-His) box helicase 30 (DHX30), tı | NM_138615 |
| uc059kmk.1 | 4.82E-04 | -2.99 | down | -1.26 | down | -2.79 | -1.21 | -1.55 | DIS3L | DIS3 like exosome 3'-5' exoribonuclease (DIS3L), tra | NM_133375 |
| uc062seb.1 | 9.10E-04 | -1.18 | down | -1.57 | down | -1.66 | -1.41 | -2.06 | **DLG1** | discs, large homolog 1 (Drosophila) (DLG1), transcri | NM_0012043 |
| uc061cxr.1 | 2.49E-05 | -1.50 | down | -6.34 | down | -3.46 | -2.87 | -5.54 | DNAAF3 | dynein, axonemal, assembly factor 3 (DNAAF3), tran | NM_0012567 |
| uc010wnn.2 | 3.37E-04 | -1.32 | down | -1.17 | down | 3.12 | 3.52 | 3.30 | DYNLL2 | dynein, light chain, LC8-type 2 (DYNLL2), mRNA. (frc | NM_080677 |
| uc059soq.1 | 5.83E-04 | 1.01 | up | -1.45 | down | 1.38 | 1.36 | 0.82 | EIF3CL | eukaryotic translation initiation factor 3, subunit C-like | NM_0010996 |
| uc002gnz.5 | 8.23E-04 | -1.36 | down | -1.26 | down | -0.62 | -0.18 | -0.52 | ELAC2 | elaC ribonuclease Z 2 | NM_018127 |
| uc057gll.1 | 5.73E-04 | -1.73 | down | -1.27 | down | -0.12 | 0.68 | 0.34 | **EPS15** | epidermal growth factor receptor pathway substrate ' | NM_001981 |
| uc059fov.1 | 8.33E-04 | -1.40 | down | -4.19 | down | -3.11 | -2.62 | -4.69 | EXOC3L4 | exocyst complex component 3-like 4 (EXOC3L4), mf | NM_0010775 |
| uc060vkb.1 | 8.37E-04 | -1.47 | down | -1.39 | down | -0.54 | 0.02 | -0.45 | FAM129C | family with sequence similarity 129, member C (FAM' | NM_0010985 |
| uc057bod.1 | 6.86E-05 | -2.70 | down | -5.95 | down | -5.93 | -4.49 | -7.07 | FAM213B, | family with sequence similarity 213, member B (FAM2 | NM_152371 |
| uc061fvb.1 | 6.86E-05 | -2.70 | down | -5.95 | down | -5.93 | -4.49 | -7.07 | FAM213B, | family with sequence similarity 213, member B (FAM2 | NM_152371 |
| uc064tfh.1 | 7.56E-04 | -2.00 | down | -2.97 | down | -3.90 | -2.90 | -4.47 | FAM95B1, | family with sequence similarity 95, member B1 (FAM9 | NR_026759 |
| uc060std.1 | 7.00E-04 | 1.21 | up | -6.37 | down | -2.71 | -2.99 | -5.66 | FBN3 | fibrillin 3 (FBN3), mRNA. (from RefSeq NM_032447) | NM_032447 |
| uc058fep.1 | 6.63E-04 | -1.37 | down | -1.52 | down | -0.02 | 0.43 | -0.18 | FCHSD2 | FCH and double SH3 domains 2 (FCHSD2), mRNA. ( | NM_014824 |
| uc064glm.1 | 5.34E-04 | -1.41 | down | -1.39 | down | 0.90 | 1.40 | 0.92 | FIS1 | fission 1 (mitochondrial outer membrane) homolog (S | NM_016068 |
| uc003djj.3 | 8.46E-04 | -1.23 | down | -1.33 | down | -0.49 | -0.18 | -0.59 | FLNB | filamin B, beta (FLNB), transcript variant 2, mRNA. (fı | NM_001457 |
| uc010hnf.3 | 7.92E-04 | -1.24 | down | -1.33 | down | -0.48 | -0.17 | -0.59 | FLNB | filamin B, beta (FLNB), transcript variant 4, mRNA. (fı | NM_0011643 |
| uc064mnv.1 | 3.41E-04 | -1.14 | down | -1.46 | down | 0.25 | 0.44 | -0.11 | FNTA | farnesyltransferase, CAAX box, alpha (FNTA), transc | NM_002027 |
| uc063okh.1 | 1.65E-04 | 1.12 | up | -4.26 | down | -1.53 | -1.69 | -3.78 | FOXP4-AS1 | FOXP4 antisense RNA 1 | NR_126417 |
| uc060atq.1 | 7.87E-04 | -1.33 | down | -1.41 | down | 1.69 | 2.10 | 1.60 | FXR2 | fragile X mental retardation, autosomal homolog 2 (F) | NM_004860 |
| uc004doi.6 | 9.23E-04 | -1.76 | down | -5.32 | down | -3.91 | -3.10 | -5.51 | GAGE13 | G antigen 2C (GAGE2C), mRNA. (from RefSeq NM_ | NM_001472 |
| uc059yvw.1 | 3.93E-05 | 1.16 | up | -4.34 | down | -2.36 | -2.57 | -4.68 | GAS8 | growth arrest-specific 8 (GAS8), transcript variant 4, | NM_0012862 |
| uc062dbe.1 | 3.38E-04 | -1.56 | down | -3.77 | down | -1.37 | -0.73 | -2.64 | **GATSL3** | GATS protein-like 3 (GATSL3), mRNA. (from RefSeq | NM_0010376 |
| uc002whp.2 | 8.97E-04 | -1.89 | down | -4.43 | down | -1.93 | -1.01 | -3.16 | GNRH2 | gonadotropin-releasing hormone 2 (GNRH2), transcr | NM_178331 |
| uc057dtw.1 | 6.14E-04 | -1.51 | down | -1.64 | down | -0.77 | -0.18 | -0.89 | GPATCH3 | G patch domain containing 3 (GPATCH3), mRNA. (fr | NM_022078 |
| uc059uvj.1 | 5.04E-04 | 1.44 | up | -4.69 | down | -4.07 | -4.60 | -6.83 | GPR56 | adhesion G protein-coupled receptor G1 (ADGRG1), | NM_0011457 |
| uc064roh.1 | 8.92E-04 | -1.17 | down | -2.68 | down | -2.36 | -2.13 | -3.56 | GPT | glutamic-pyruvate transaminase (alanine aminotransf | NM_005309 |
| uc063ixf.1 | 3.23E-04 | -1.84 | down | -3.71 | down | -4.06 | -3.19 | -5.08 | GRIA1 | glutamate receptor, ionotropic, AMPA 1 (GRIA1), trar | NM_0012580 |
| uc002iii.2 | 3.77E-04 | -1.30 | down | -1.54 | down | -1.01 | -0.64 | -1.26 | HEXIM2 | hexamethylene bis-acetamide inducible 2 | NM_0013034 |
| uc061afd.1 | 8.69E-04 | -1.88 | down | -5.50 | down | -2.56 | -1.65 | -4.11 | HIF3A | hypoxia inducible factor 3, alpha subunit (HIF3A), trai | NM_152796 |

| | | | | | | | | | | | |
|---|---|---|---|---|---|---|---|---|---|---|---|
| uc058uor.1 | 8.95E-04 | -1.38 | down | -1.34 | down | -0.14 | 0.33 | -0.09 | HIP1R | huntingtin interacting protein 1 related (HIP1R), trans | NM_003959 |
| uc061yty.1 | 1.48E-04 | -1.62 | down | -1.49 | down | -2.68 | -1.99 | -2.56 | **ICOSLG** | inducible T-cell costimulator ligand | NM_0012830 |
| uc058pgk.1 | 8.59E-04 | -3.36 | down | -3.40 | down | -4.45 | -2.69 | -4.46 | **IKZF4** | IKAROS family zinc finger 4 (Eos) (IKZF4), mRNA. (f | NM_022465 |
| uc063ljh.1 | 9.84E-04 | -1.59 | down | -1.63 | down | -0.30 | 0.37 | -0.34 | **IRF4** | interferon regulatory factor 4 | NM_0011952 |
| uc061nqc.1 | 8.13E-04 | -1.31 | down | -1.45 | down | -0.04 | 0.35 | -0.19 | IWS1 | IWS1 homolog (S. cerevisiae) (IWS1), mRNA. (from I | NM_017969 |
| uc063hbv.1 | 5.15E-04 | -1.64 | down | -1.58 | down | -0.30 | 0.41 | -0.24 | JADE2 | jade family PHD finger 2 (JADE2), transcript variant 3 | NM_015288 |
| uc062lig.1 | 9.27E-04 | -1.18 | down | -3.34 | down | -0.79 | -0.55 | -2.29 | KBTBD8 | kelch repeat and BTB (POZ) domain containing 8 (KE | NM_032505 |
| uc064zso.1 | 6.27E-04 | -1.32 | down | -3.69 | down | -1.29 | -0.89 | -2.78 | KIF4A | kinesin family member 4A (KIF4A), mRNA. (from Ref | NM_012310 |
| uc057ohz.1 | 9.46E-04 | -1.89 | down | -4.43 | down | -2.06 | -1.14 | -3.29 | LAD1 | ladinin 1 (LAD1), mRNA. (from RefSeq NM_005558) | NM_005558 |
| uc063epk.1 | 3.51E-04 | -1.29 | down | -4.69 | down | -0.14 | 0.22 | -2.01 | LINC01336 | long intergenic non-protein coding RNA 1336 (LINC0 | NR_126375 |
| uc063ady.1 | 5.93E-04 | -1.34 | down | -1.61 | down | 0.19 | 0.61 | -0.07 | **LRBA** | LPS-responsive vesicle trafficking, beach and ancho | NM_006726 |
| uc058kne.1 | 6.65E-04 | -1.52 | down | -2.57 | down | -2.24 | -1.64 | -3.00 | LRRC23 | leucine rich repeat containing 23 (LRRC23), transcrip | NM_006992 |
| uc060ltc.1 | 7.33E-04 | -1.18 | down | -1.72 | down | 0.64 | 0.88 | 0.09 | LRRC45 | leucine rich repeat containing 45 (LRRC45), mRNA. ( | NM_144999 |
| uc063ecv.1 | 7.05E-04 | -1.02 | down | -3.11 | down | -1.58 | -1.55 | -3.19 | MAST4 | microtubule associated serine/threonine kinase family | NM_0012902 |
| uc062pue.1 | 5.60E-06 | -1.59 | down | -8.01 | down | -5.26 | -4.59 | -7.60 | MECOM | MDS1 and EVI1 complex locus (MECOM), transcript | NM_0011050 |
| uc032ylk.1 | 4.00E-04 | -1.28 | down | -10.06 | down | -0.31 | 0.05 | -3.28 | MIR3135B | microRNA 3135b (MIR3135B), microRNA. (from Ref | NR_039668 |
| uc032ytr.1 | 4.00E-04 | -1.28 | down | -10.06 | down | -0.31 | 0.05 | -3.28 | MIR3135B | microRNA 3135b (MIR3135B), microRNA. (from Ref | NR_039668 |
| uc021uqm.1 | 4.43E-04 | -1.30 | down | -24.62 | down | -0.88 | -0.50 | -5.12 | MIR3188 | microRNA 3188 (MIR3188), microRNA. (from RefSec | NR_036155 |
| uc058vgw.1 | 2.07E-04 | -1.69 | down | -1.83 | down | -2.04 | -1.28 | -2.15 | MMP17 | matrix metallopeptidase 17 (membrane-inserted) (MN | NM_016155 |
| uc063sgp.1 | 7.99E-04 | -2.35 | down | -6.59 | down | -2.57 | -1.34 | -4.06 | **MTHFD1L** | methylenetetrahydrofolate dehydrogenase (NADP+ d | NM_0012427 |
| uc060ehm.1 | 3.05E-04 | -1.15 | down | -4.41 | down | -2.51 | -2.30 | -4.44 | MYO19 | myosin XIX (MYO19), transcript variant 3, mRNA. (fr | NM_0010335 |
| uc064kqr.1 | 8.65E-04 | -2.37 | down | -5.37 | down | -2.71 | -1.46 | -3.89 | NAT1 | N-acetyltransferase 1 (arylamine N-acetyltransferase | NM_0011601 |
| uc057jyz.1 | 8.12E-04 | -1.42 | down | -1.53 | down | -0.50 | 0.01 | -0.60 | NBPF15 | neuroblastoma breakpoint family, member 15 (NBPF | NM_173638 |
| uc057jza.1 | 3.65E-04 | -1.52 | down | -1.63 | down | -0.15 | 0.46 | -0.25 | NBPF15 | neuroblastoma breakpoint family, member 15 (NBPF | NM_173638 |
| uc061end.1 | 3.17E-04 | -3.02 | down | -2.71 | down | -6.16 | -4.57 | -6.01 | NLRP2 | NLR family, pyrin domain containing 2 (NLRP2), trans | NM_0011740 |
| uc061ctz.1 | 5.74E-04 | -1.21 | down | -3.43 | down | -5.62 | -5.34 | -7.12 | NLRP7 | NLR family, pyrin domain containing 7 (NLRP7), trans | NM_0011272 |
| uc061foc.1 | 5.74E-04 | -1.21 | down | -3.43 | down | -5.62 | -5.34 | -7.12 | NLRP7 | NLR family, pyrin domain containing 7 (NLRP7), trans | NM_0011272 |
| uc061var.1 | 2.61E-04 | -1.38 | down | -1.50 | down | 0.02 | 0.49 | -0.10 | NOP56 | NOP56 ribonucleoprotein (NOP56), transcript variant | NM_006392 |
| uc061mjc.1 | 1.47E-04 | -1.26 | down | -4.39 | down | -2.81 | -2.48 | -4.61 | NPAS2 | neuronal PAS domain protein 2 (NPAS2), mRNA. (fro | NM_002518 |
| uc058cye.1 | 1.93E-04 | 1.08 | up | -1.87 | down | -1.45 | -1.56 | -2.46 | NRXN2 | neurexin 2 (NRXN2), transcript variant beta, mRNA. | NM_138734 |
| uc059six.1 | 1.07E-05 | -2.04 | down | -1.12 | down | 0.00 | 1.03 | 0.87 | NSMCE1 | non-SMC element 1 homolog (S. cerevisiae) (NSMCE | NM_145080 |
| uc021txo.2 | 4.75E-06 | -1.86 | down | -1.54 | down | -2.28 | -1.39 | -2.00 | NT5C3B | 5'-nucleotidase, cytosolic IIIB (NT5C3B), transcript v | NM_052935 |
| uc060fdr.1 | 8.67E-04 | -1.51 | down | -1.46 | down | -1.86 | -1.27 | -1.82 | NT5C3B | 5'-nucleotidase, cytosolic IIIB (NT5C3B), transcript v | NM_052935 |
| uc061aza.1 | 3.59E-04 | -1.43 | down | -2.00 | down | 0.14 | 0.66 | -0.34 | NTF4 | neurotrophin 4 (NTF4), mRNA. (from RefSeq NM_00 | NM_006179 |
| uc062xlo.1 | 2.87E-04 | -1.15 | down | -1.54 | down | 1.17 | 1.37 | 0.74 | **NUP54** | nucleoporin 54kDa | NR_103781 |
| uc062xlr.1 | 6.18E-04 | -1.26 | down | -1.47 | down | -0.24 | 0.09 | -0.46 | NUP54 | nucleoporin 54kDa | NR_103782 |
| uc061bmx.1 | 7.54E-04 | -1.45 | down | -1.38 | down | -0.62 | -0.09 | -0.55 | NUP62 | nucleoporin 62kDa (NUP62), transcript variant 1, mR | NM_153719 |
| uc057jvt.1 | 6.40E-04 | -1.51 | down | -1.72 | down | -1.12 | -0.53 | -1.31 | PHGDH | phosphoglycerate dehydrogenase (PHGDH), mRNA | NM_006623 |
| uc064dvi.1 | 1.39E-04 | -1.47 | down | -4.86 | down | -2.58 | -2.02 | -4.30 | PHKG1 | phosphorylase kinase, gamma 1 | NR_047689 |
| uc057xni.1 | 1.96E-04 | -6.37 | down | -2.10 | down | -4.38 | -1.71 | -2.78 | PIDD1 | p53-induced death domain protein 1 (PIDD1), transcr | NM_145887 |
| uc059phr.1 | 1.70E-04 | -1.82 | down | -1.62 | down | -1.22 | -0.36 | -1.06 | PKD1 | polycystic kidney disease 1 (autosomal dominant) (P | NM_000296 |
| uc059rbn.1 | 8.11E-04 | -1.42 | down | -1.91 | down | 1.87 | 2.38 | 1.45 | **PLA2G10,** | phospholipase A2, group X (PLA2G10), mRNA. (from | NM_003561 |
| uc062iet.1 | 4.03E-04 | -1.75 | down | -1.38 | down | -0.98 | -0.17 | -0.63 | PLCD1 | phospholipase C, delta 1 (PLCD1), transcript variant | NM_006225 |
| uc061xai.1 | 5.74E-04 | -1.75 | down | -1.74 | down | -0.55 | 0.25 | -0.54 | **PLCG1** | phospholipase C, gamma 1 (PLCG1), transcript varia | NM_182811 |
| uc062fel.1 | 1.70E-04 | -1.09 | down | -1.65 | down | -1.30 | -1.18 | -1.90 | **PPARA** | peroxisome proliferator activated receptor alpha | NM_0010019 |

| | | | | | | | | | | |
|---|---|---|---|---|---|---|---|---|---|---|
| uc063msm.1 | 2.76E-04 | 1.41 | up | 1.21 | up | -0.05 | -0.54 | -0.26 | PPP1R10 | protein phosphatase 1 regulatory subunit 10 | NR_072994 |
| uc063twf.1 | 2.76E-04 | 1.41 | up | 1.21 | up | -0.05 | -0.54 | -0.26 | PPP1R10 | protein phosphatase 1 regulatory subunit 10 | NR_072994 |
| uc063vcd.1 | 2.76E-04 | 1.41 | up | 1.21 | up | -0.05 | -0.54 | -0.26 | PPP1R10 | protein phosphatase 1 regulatory subunit 10 | NR_072994 |
| uc063whs.1 | 2.76E-04 | 1.41 | up | 1.21 | up | -0.05 | -0.54 | -0.26 | PPP1R10 | protein phosphatase 1 regulatory subunit 10 | NR_072994 |
| uc063xhb.1 | 2.76E-04 | 1.41 | up | 1.21 | up | -0.05 | -0.54 | -0.26 | PPP1R10 | protein phosphatase 1 regulatory subunit 10 | NR_072994 |
| uc063ymx.1 | 8.50E-04 | 1.35 | up | 1.26 | up | -0.18 | -0.61 | -0.28 | PPP1R10 | protein phosphatase 1 regulatory subunit 10 | NR_072994 |
| uc063zss.1 | 2.76E-04 | 1.41 | up | 1.21 | up | -0.05 | -0.54 | -0.26 | PPP1R10 | protein phosphatase 1 regulatory subunit 10 | NR_072994 |
| uc063phe.1 | 5.06E-04 | -1.18 | down | -3.78 | down | -0.54 | -0.30 | -2.22 | PRIM2 | primase, DNA, polypeptide 2 (58kDa) (PRIM2), trans | NM_000947 |
| uc010vlg.3 | 5.44E-04 | -1.29 | down | -1.32 | down | -1.65 | -1.29 | -1.69 | PRMT7 | protein arginine methyltransferase 7 (PRMT7), transc | NM_0011848 |
| uc064wqo.1 | 7.50E-04 | -1.36 | down | -1.63 | down | 1.44 | 1.89 | 1.18 | PRRC2B | proline-rich coiled-coil 2B (PRRC2B), mRNA. (from R | NM_013318 |
| uc063mxz.1 | 3.53E-04 | -4.06 | down | -5.22 | down | -3.46 | -1.44 | -3.82 | **PSORS1C1,** | psoriasis susceptibility 1 candidate 1 (PSORS1C1), r | NM_014068 |
| uc058mvt.1 | 3.69E-04 | -1.43 | down | -1.54 | down | -1.05 | -0.53 | -1.16 | PUS7L | pseudouridylate synthase 7 homolog (S. cerevisiae)- | NM_0010986 |
| uc058jqr.1 | 9.54E-04 | -1.26 | down | -2.85 | down | -2.29 | -1.96 | -3.46 | RAD52 | RAD52 homolog, DNA repair protein | NM_0012974 |
| uc060sfo.1 | 7.58E-04 | -1.14 | down | -2.37 | down | -1.38 | -1.19 | -2.44 | RANBP3 | RAN binding protein 3 (RANBP3), transcript variant 4 | NM_0013008 |
| uc058tuf.1 | 8.53E-04 | -1.64 | down | -1.43 | down | -1.54 | -0.83 | -1.34 | RFC5 | replication factor C subunit 5 | NM_0012068 |
| uc059xcn.1 | 9.57E-04 | -1.43 | down | -1.59 | down | -0.58 | -0.06 | -0.73 | RFWD3 | ring finger and WD repeat domain 3 (RFWD3), mRNA | NM_018124 |
| uc064qmg.1 | 6.26E-04 | -2.98 | down | 1.08 | up | -1.20 | 0.38 | 0.48 | RP11-157E2 | uncharacterized LOC101927822 (LOC101927822), l | NR_125424 |
| uc057vee.1 | 4.89E-04 | -1.20 | down | -4.26 | down | 0.24 | 0.50 | -1.59 | RRP12 | ribosomal RNA processing 12 homolog (S. cerevisiae | NM_0012843 |
| uc060hiq.1 | 7.49E-04 | -1.21 | down | -1.53 | down | -0.26 | 0.01 | -0.60 | RSAD1 | radical S-adenosyl methionine domain containing 1 (F | NM_018346 |
| uc062oaq.1 | 6.40E-04 | -1.39 | down | -1.51 | down | -0.36 | 0.11 | -0.48 | RYK | receptor-like tyrosine kinase (RYK), transcript varian | NM_002958 |
| uc064bpk.1 | 5.85E-04 | -1.30 | down | -4.92 | down | -1.40 | -1.02 | -3.32 | SCIN | scinderin (SCIN), transcript variant 2, mRNA. (from F | NM_033128 |
| uc063exq.1 | 4.46E-04 | -1.38 | down | -1.54 | down | 1.30 | 1.76 | 1.14 | SERINC5 | serine incorporator 5 | NM_0011740 |
| uc062evq.1 | 7.61E-05 | -1.37 | down | -1.25 | down | -0.76 | -0.31 | -0.63 | SERLH2 | serine hydrolase-like 2 | NR_104301 |
| uc062evt.1 | 4.01E-04 | -1.42 | down | -1.28 | down | -0.95 | -0.44 | -0.80 | SERLH2 | serine hydrolase-like 2 | NR_104300 |
| uc062bvk.1 | 8.81E-04 | -1.52 | down | -1.42 | down | -0.80 | -0.19 | -0.69 | **SERPIND1** | serpin peptidase inhibitor, clade D (heparin cofactor), | NM_000185 |
| uc062djs.1 | 4.74E-04 | -1.35 | down | -1.33 | down | -0.73 | -0.29 | -0.71 | SFI1 | Sfi1 homolog, spindle assembly associated (yeast) (S | NM_014775 |
| uc001zwr.5 | 9.10E-04 | 1.02 | up | 1.67 | up | 0.59 | 0.56 | 1.30 | SLC12A1 | solute carrier family 12 (sodium/potassium/chloride tr | NM_000338 |
| uc059wbp.1 | 5.97E-04 | -1.62 | down | -1.24 | down | -1.68 | -0.98 | -1.29 | SLC12A4 | solute carrier family 12 (potassium/chloride transporte | NM_0011459 |
| uc064wao.1 | 3.99E-04 | -1.80 | down | -1.66 | down | -1.08 | -0.23 | -0.96 | SLC25A25 | solute carrier family 25 (mitochondrial carrier; phosph | NM_052901 |
| uc063fxo.1 | 5.55E-04 | 1.02 | up | -1.81 | down | 0.47 | 0.44 | -0.41 | SLC25A46 | solute carrier family 25 member 46 | NM_0013032 |
| uc062iym.1 | 2.16E-04 | -2.20 | down | -1.14 | down | -0.19 | 0.95 | 0.76 | SLC6A20 | solute carrier family 6 (proline IMINO transporter), me | NM_022405 |
| uc002cxt.4 | 7.09E-04 | -1.07 | down | -3.14 | down | -1.91 | -1.81 | -3.47 | SMIM22 | small integral membrane protein 22 (SMIM22), transc | NM_0012537 |
| uc059qjk.1 | 6.54E-04 | -1.24 | down | -3.31 | down | -1.76 | -1.45 | -3.18 | SMIM22 | small integral membrane protein 22 (SMIM22), transc | NM_0012537 |
| uc061ver.1 | 8.60E-04 | -1.43 | down | -1.45 | down | 1.02 | 1.53 | 0.99 | SMOX | spermine oxidase (SMOX), transcript variant 2, mRN | NM_175840 |
| uc057qye.1 | 2.67E-04 | -1.35 | down | -3.32 | down | -2.80 | -2.37 | -4.10 | **SMYD3** | SET and MYND domain containing 3 (SMYD3), trans | NM_022743 |
| uc064hsz.1 | 1.85E-04 | -1.16 | down | -1.53 | down | 1.46 | 1.67 | 1.06 | SND1 | staphylococcal nuclease and tudor domain containing | NM_014390 |
| uc001tfu.2 | 1.20E-04 | 1.01 | up | -6.60 | down | 0.68 | 0.67 | -2.05 | SNORA53 | small nucleolar RNA, H/ACA box 53 (SNORA53), sm | NR_003015 |
| uc064tit.1 | 1.93E-04 | -2.64 | down | -7.67 | down | -4.13 | -2.73 | -5.67 | SPATA31A7 | SPATA31 subfamily A, member 7 (SPATA31A7), mRI | NM_015667 |
| uc063eby.1 | 8.86E-04 | -1.33 | down | -1.55 | down | 0.36 | 0.77 | 0.14 | SREK1 | splicing regulatory glutamine/lysine-rich protein 1 (SR | NM_0012704 |
| uc058mcg.1 | 4.95E-04 | -1.60 | down | -2.98 | down | -1.23 | -0.55 | -2.13 | SSPN | sarcospan (SSPN), transcript variant 2, mRNA. (from | NM_0011358 |
| uc010rnb.2 | 9.82E-04 | -1.41 | down | -1.35 | down | 1.35 | 1.84 | 1.41 | STIP1 | stress-induced phosphoprotein 1 (STIP1), transcript | NM_0012826 |
| uc057jcy.1 | 5.62E-04 | -1.55 | down | -1.35 | down | 0.07 | 0.71 | 0.27 | STRIP1 | striatin interacting protein 1 (STRIP1), transcript varia | NM_0012707 |
| uc064egv.1 | 8.06E-04 | -1.10 | down | -2.56 | down | -1.75 | -1.61 | -2.96 | STX1A | syntaxin 1A (brain) (STX1A), transcript variant 2, mR | NM_0011659 |
| uc059pdz.1 | 4.54E-04 | -1.52 | down | -1.21 | down | -1.20 | -0.60 | -0.87 | TBL3 | transducin (beta)-like 3 (TBL3), mRNA. (from RefSec | NM_006453 |
| uc060rek.1 | 4.39E-04 | -1.39 | down | -1.29 | down | -0.33 | 0.14 | -0.23 | **TCF3** | transcription factor 3 (TCF3), transcript variant 1, mF | NM_003200 |

| | | | | | | | | | | | |
|---|---|---|---|---|---|---|---|---|---|---|---|
| uc061znt.1 | 5.57E-04 | -1.77 | down | -1.31 | down | -1.83 | -1.01 | -1.40 | TCP10L | t-complex 10-like (TCP10L), mRNA. (from RefSeq NI | NM_144659 |
| uc057kyy.1 | 9.60E-04 | -1.30 | down | -1.29 | down | -2.19 | -1.81 | -2.17 | TDRKH | tudor and KH domain containing (TDRKH), transcript | NM_0010839 |
| uc060upn.1 | 4.93E-04 | -1.92 | down | -1.60 | down | -1.19 | -0.25 | -0.92 | TECR | trans-2,3-enoyl-CoA reductase (TECR), transcript va | NM_138501 |
| uc061ojm.1 | 9.66E-04 | -2.23 | down | -6.50 | down | -2.10 | -0.95 | -3.65 | TEX41 | testis expressed 41 (non-protein coding) (TEX41), lo | NR_033870 |
| uc061irb.1 | 5.78E-05 | -2.01 | down | -1.74 | down | -1.11 | -0.10 | -0.91 | THADA | thyroid adenoma associated | NR_073394 |
| uc061kij.1 | 8.52E-04 | -1.45 | down | -1.55 | down | 0.39 | 0.92 | 0.29 | **TIA1** | TIA1 cytotoxic granule-associated RNA binding prote | NM_022173 |
| uc060vxm.1 | 4.76E-06 | -3.08 | down | -10.76 | down | -2.48 | -0.86 | -4.28 | TMEM161A | transmembrane protein 161A (TMEM161A), transcrip | NM_0012567 |
| uc062bjc.1 | 4.17E-05 | -1.39 | down | -6.22 | down | -2.72 | -2.25 | -4.89 | TMEM191B | transmembrane protein 191B (TMEM191B), mRNA. ( | NM_0012423 |
| uc058ssh.1 | 1.51E-05 | -1.43 | down | -1.62 | down | -0.05 | 0.46 | -0.24 | TMEM263 | transmembrane protein 263 (TMEM263), mRNA. (fro | NM_152261 |
| uc064ooz.1 | 6.35E-04 | 1.04 | up | -3.40 | down | -3.32 | -3.38 | -5.14 | TMEM67 | transmembrane protein 67 | NR_024522 |
| uc064vgq.1 | 0 | -1.00 | down | -1.00 | down | -1.87 | -1.87 | -1.87 | TNC | tenascin C (TNC), mRNA. (from RefSeq NM_002160 | NM_002160 |
| uc061duf.1 | 4.36E-04 | -1.33 | down | -1.51 | down | 1.05 | 1.46 | 0.87 | **TRIM28** | tripartite motif containing 28 (TRIM28), mRNA. (from | NM_005762 |
| uc058qdh.1 | 9.20E-04 | -1.61 | down | -1.36 | down | -1.66 | -0.97 | -1.41 | TSFM | Ts translation elongation factor, mitochondrial (TSFM | NM_005726 |
| uc062atf.1 | 8.31E-04 | -1.41 | down | -1.34 | down | 0.24 | 0.73 | 0.32 | UBE2G2 | ubiquitin-conjugating enzyme E2G 2 (UBE2G2), trans | NM_003343 |
| uc058dep.1 | 4.94E-04 | -1.37 | down | -1.52 | down | 1.35 | 1.80 | 1.19 | VPS51 | vacuolar protein sorting 51 homolog (S. cerevisiae) (\ | NM_013265 |
| uc058fld.1 | 8.78E-05 | -1.23 | down | -4.33 | down | -3.01 | -2.72 | -4.83 | XRRA1 | X-ray radiation resistance associated 1 (XRRA1), tra | NM_0012703 |
| uc060pua.1 | 1.92E-04 | -1.24 | down | -1.59 | down | 1.15 | 1.46 | 0.79 | ZCCHC2 | zinc finger, CCHC domain containing 2 (ZCCHC2), tr | NM_017742 |
| uc063ezp.1 | 8.63E-04 | -1.16 | down | -3.86 | down | -1.01 | -0.80 | -2.74 | ZCCHC9 | zinc finger, CCHC domain containing 9 (ZCCHC9), tr | NM_0011310 |
| uc001knl.5 | 9.98E-04 | -1.25 | down | -1.23 | down | -1.75 | -1.43 | -1.73 | ZDHHC16 | zinc finger, DHHC-type containing 16 (ZDHHC16), tr | NM_198043 |
| uc057vfd.1 | 3.53E-04 | -1.65 | down | -1.61 | down | -1.07 | -0.35 | -1.04 | ZDHHC16 | zinc finger, DHHC-type containing 16 (ZDHHC16), tr | NM_0012878 |
| uc061you.1 | 1.46E-04 | -1.45 | down | -1.61 | down | -0.91 | -0.38 | -1.07 | ZGPAT | zinc finger, CCCH-type with G patch domain (ZGPAT | NM_181485 |
| uc010tbw.3 | 9.73E-04 | -1.56 | down | -1.40 | down | -2.72 | -2.09 | -2.57 | ZNF268 | zinc finger protein 268 (ZNF268), transcript variant 5 | NM_0011658 |
| uc001ico.4 | 3.87E-04 | -1.34 | down | -1.20 | down | -0.64 | -0.21 | -0.47 | ZNF496 | zinc finger protein 496 (ZNF496), mRNA. (from RefS | NM_032752 |
| uc002mtq.3 | 7.41E-04 | -1.46 | down | -1.46 | down | -1.27 | -0.72 | -1.26 | ZNF563 | zinc finger protein 563 (ZNF563), mRNA. (from RefS | NM_145276 |
| uc060zmo.1 | 3.04E-04 | -1.91 | down | -1.59 | down | -0.53 | 0.41 | -0.26 | ZNF576 | zinc finger protein 576 (ZNF576), transcript variant 2 | NM_0011453 |
| uc061dni.1 | 6.00E-04 | -1.27 | down | -1.63 | down | -0.91 | -0.56 | -1.26 | ZNF587 | zinc finger protein 587 (ZNF587), transcript variant 2 | NM_0012048 |
| uc062iuz.1 | 1.24E-04 | -1.38 | down | -1.39 | down | -0.68 | -0.22 | -0.69 | ZNF852 | zinc finger protein 852 (ZNF852), mRNA. (from RefS | NM_0012873 |
| uc002bkr.4 | 1.09E-04 | -1.43 | down | -1.29 | down | -2.09 | -1.58 | -1.94 | ZSCAN2 | zinc finger and SCAN domain containing 2 (ZSCAN2) | NM_181877 |
| uc059mrx.1 | 4.71E-05 | -1.45 | down | -1.25 | down | -2.04 | -1.50 | -1.82 | ZSCAN2 | zinc finger and SCAN domain containing 2 (ZSCAN2) | NM_181877 |
| uc059mry.1 | 9.45E-04 | -1.38 | down | -1.52 | down | -1.07 | -0.60 | -1.20 | ZSCAN2 | zinc finger and SCAN domain containing 2 (ZSCAN2) | NM_181877 |
| uc059msa.1 | 1.12E-04 | -1.45 | down | -1.20 | down | -1.64 | -1.11 | -1.36 | ZSCAN2 | zinc finger and SCAN domain containing 2 (ZSCAN2) | NM_181877 |

**Supplementary Table 2. Gender sensitive transcripts** **Values are log2 RPKM**

| GeneName | p | FC (abs) | Regulation | [F](normaliz | [M](normaliz | Symbol | Description | RefSeq |
|---|---|---|---|---|---|---|---|---|
| uc064gjl.1 | 0.01938091 | 3.9134712 | up | -2.2245476 | -4.1929965 | ACHE | Homo sapiens acetylcholinesterase (Yt blood group) (ACHE), transcript variant 3, m | NM_001282449 |
| uc010itk.2 | 0.03062528 | 3.39541 | down | -4.2712855 | -2.5076997 | ADAMTS16 | Homo sapiens ADAM metallopeptidase with thrombospondin type 1 motif, 16 (ADA | NM_139056 |
| uc060emi.1 | 0.00389895 | 3.0257018 | up | -0.4121704 | -2.0094402 | ARHGAP23, | Homo sapiens Rho GTPase activating protein 23 (ARHGAP23), mRNA. (from RefSe | NM_001199417 |
| uc064phw.1 | 0.01207369 | 3.2448657 | down | -2.4748294 | -0.7766708 | BAALC | Homo sapiens brain and acute leukemia, cytoplasmic (BAALC), transcript variant 2, | NM_001024372 |
| uc062ahg.1 | 0.02366242 | 3.1006773 | down | -4.0203047 | -2.3877213 | BACE2 | Homo sapiens beta-site APP-cleaving enzyme 2 (BACE2), transcript variant c, mRN | NM_138991 |
| uc060llv.1 | 0.01410843 | 3.1679745 | down | -2.760526 | -1.0969652 | CCDC137 | Homo sapiens coiled-coil domain containing 137 (CCDC137), mRNA. (from RefSeq I | NM_199287 |
| uc057tex.1 | 1.43E-04 | 4.8776813 | down | -5.13456 | -2.8483646 | CHAT | Homo sapiens choline O-acetyltransferase (CHAT), transcript variant N2, mRNA. (fr | NM_020986 |
| uc062nwr.1 | 0.00653113 | 3.3447895 | down | -5.167503 | -3.4255874 | CPNE4 | Homo sapiens copine IV (CPNE4), transcript variant 2, mRNA. (from RefSeq NM_15 | NM_153429 |
| uc004fsu.2 | 5.25E-29 | 5.0172653 | down | -0.817907 | 1.5089942 | DDX3Y | Homo sapiens DEAD (Asp-Glu-Ala-Asp) box helicase 3, Y-linked (DDX3Y), transcript | NM_001122665 |
| uc004fsv.3 | 1.89E-19 | 4.272347 | down | 0.9116861 | 3.006715 | DDX3Y | Homo sapiens DEAD (Asp-Glu-Ala-Asp) box helicase 3, Y-linked (DDX3Y), transcript | NM_004660 |
| uc065crs.1 | 2.24E-31 | 7.2224665 | down | -2.2035515 | 0.64894 | DDX3Y | Homo sapiens DEAD (Asp-Glu-Ala-Asp) box helicase 3, Y-linked (DDX3Y), transcript | NM_001122665 |
| uc002wvp.1 | 0.00206359 | 3.7402442 | down | -0.8678935 | 1.035239 | DEFB115 | Homo sapiens defensin, beta 115 (DEFB115), mRNA. (from RefSeq NM_00103773C | NM_001037730 |
| uc064bwk.1 | 0.00406922 | 3.6024404 | down | -3.4191754 | -1.5702008 | DNAH11 | Homo sapiens dynein, axonemal, heavy chain 11 (DNAH11), mRNA. (from RefSeq N | NM_001277115 |
| uc004fuk.5 | 6.28E-38 | 21.4298 | down | 0.2558369 | 4.6773834 | EIF1AY | Homo sapiens eukaryotic translation initiation factor 1A, Y-linked (EIF1AY), transcri | NM_004681 |
| uc004ful.5 | 4.38E-26 | 18.869068 | down | -0.9159175 | 3.3220336 | EIF1AY | Homo sapiens eukaryotic translation initiation factor 1A, Y-linked (EIF1AY), transcri | NM_001278612 |
| uc065cuc.1 | 3.29E-19 | 7.0512075 | down | -0.5066402 | 2.3112302 | EIF1AY | Homo sapiens eukaryotic translation initiation factor 1A, Y-linked (EIF1AY), transcri | NM_004681 |
| uc065cue.1 | 3.04E-25 | 4.044944 | down | -0.9418847 | 1.0742351 | EIF1AY | Homo sapiens eukaryotic translation initiation factor 1A, Y-linked (EIF1AY), transcri | NM_001278612 |
| uc064owu.1 | 0.01960526 | 3.3898225 | up | -1.1121063 | -2.873316 | HRSP12 | Homo sapiens heat-responsive protein 12 (HRSP12), mRNA. (from RefSeq NM_005 | NM_005836 |
| uc059wsc.1 | 0.0136463 | 3.0066426 | up | -0.7712486 | -2.359402 | HYDIN | Homo sapiens HYDIN, axonemal central pair apparatus protein (HYDIN), transcript | NM_001270974 |
| uc004fug.4 | 1.02E-27 | 4.0219693 | down | -1.6747676 | 0.33313462 | KDM5D | Homo sapiens lysine (K)-specific demethylase 5D (KDM5D), transcript variant 2, mF | NM_004653 |
| uc010nwy.4 | 4.32E-27 | 3.7597651 | down | -1.6782808 | 0.23236163 | KDM5D | Homo sapiens lysine (K)-specific demethylase 5D (KDM5D), transcript variant 3, mF | NM_001146706 |
| uc011naz.3 | 7.70E-28 | 3.5404263 | down | -1.4008155 | 0.42310765 | KDM5D | Homo sapiens lysine (K)-specific demethylase 5D (KDM5D), transcript variant 1, mF | NM_001146705 |
| uc065ctw.1 | 1.47E-27 | 4.54361 | down | -1.9088793 | 0.27495986 | KDM5D | Homo sapiens lysine (K)-specific demethylase 5D (KDM5D), transcript variant 2, mF | NM_004653 |
| uc065cua.1 | 3.20E-32 | 9.69576 | down | -2.2312317 | 1.0461223 | KDM5D | Homo sapiens lysine (K)-specific demethylase 5D (KDM5D), transcript variant 2, mF | NM_004653 |
| uc059sio.1 | 0.02388188 | 3.6873474 | down | -4.13435 | -2.2517664 | KDM8 | Homo sapiens lysine (K)-specific demethylase 8 (KDM8), transcript variant 1, mRN | NM_001145348 |
| uc031tkv.2 | 1.08E-19 | 4.9962826 | down | 0.37769195 | 2.6985471 | LINC00278 | Homo sapiens long intergenic non-protein coding RNA 278 (LINC00278), long non-c | NR_046502 |
| uc065cpm.1 | 1.70E-10 | 5.5638766 | down | -0.3208716 | 2.1552188 | LINC00278 | Homo sapiens long intergenic non-protein coding RNA 278 (LINC00278), long non-c | NR_046502 |
| uc058vvi.1 | 0.00283394 | 3.451988 | down | -1.6120982 | 0.17532915 | LINC01046 | Homo sapiens long intergenic non-protein coding RNA 1046 (LINC01046), long non- | NR_125745 |
| uc058xnv.1 | 0.00866287 | 3.8844743 | up | -0.5234225 | -2.4811418 | LMO7DN-IT1 | Homo sapiens LMO7DN intronic transcript 1 (LMO7DN-IT1), long non-coding RNA. ( | NR_126373 |
| uc064smd.1 | 0.00512486 | 3.2028503 | down | -4.4714017 | -2.7920454 | LRRC19 | Homo sapiens leucine rich repeat containing 19 (LRRC19), mRNA. (from RefSeq NN | NM_022901 |
| uc032blj.1 | 0.04585941 | 4.146925 | down | -3.0917873 | -1.0397455 | MIR151B | Homo sapiens microRNA 151b (MIR151B), microRNA. (from RefSeq NR_039601) | NR_039601 |
| uc011jvv.2 | 0.03729176 | 4.2927227 | up | -0.6004785 | -2.7023714 | MIR339 | Homo sapiens microRNA 339 (MIR339), microRNA. (from RefSeq NR_029898) | NR_029898 |
| uc021qaz.1 | 0.02102952 | 6.3192425 | down | -2.5657182 | 0.09403343 | MIR378C | Homo sapiens microRNA 378c (MIR378C), microRNA. (from RefSeq NR_036180) | NR_036180 |
| uc033ctu.1 | 0.03079745 | 3.9283636 | up | -0.2509968 | -2.2249253 | MIR4477B | Homo sapiens microRNA 4477b (MIR4477B), microRNA. (from RefSeq NR_039689 | NR_039689 |
| uc032rvy.1 | 0.01692812 | 4.686864 | up | 0.85472345 | -1.3738996 | MIR548AB | Homo sapiens microRNA 548ab (MIR548AB), microRNA. (from RefSeq NR_039611 | NR_039611 |
| uc021uev.1 | 0.03646325 | 4.269607 | up | 0.18030404 | -1.9137993 | MIR657 | Homo sapiens microRNA 657 (MIR657), microRNA. (from RefSeq NR_030394) | NR_030394 |
| uc033ccb.1 | 0.00573946 | 6.324716 | up | -1.1741376 | -3.8351383 | MIR6845 | Homo sapiens microRNA 6845 (MIR6845), microRNA. (from RefSeq NR_106904) | NR_106904 |
| uc032dpx.1 | 0.01469008 | 5.113097 | up | -0.7466453 | -3.1008427 | MIR8065 | Homo sapiens microRNA 8065 (MIR8065), microRNA. (from RefSeq NR_107032) | NR_107032 |
| uc059plp.1 | 0.02914493 | 4.9069304 | down | -3.316861 | -1.02204 | MIR940 | Homo sapiens microRNA 940 (MIR940), microRNA. (from RefSeq NR_030636) | NR_030636 |
| uc004aul.3 | 0.00267105 | 6.402918 | up | 0.7379887 | -1.940741 | MIRLET7D | Homo sapiens microRNA let-7d (MIRLET7D), microRNA. (from RefSeq NR_029481) | NR_029481 |
| uc001fie.4 | 6.87E-04 | 4.0007987 | down | -6.4688354 | -4.4685473 | MUC1 | Homo sapiens mucin 1, cell surface associated (MUC1), transcript variant 21, mRN | NM_001204297 |
| uc011bee.3 | 0.02034955 | 4.12627 | up | 0.17895718 | -1.865881 | MIRLET7G | microRNA let-7g (from HGNC MIRLET7G) | NR_029660 |
| uc022ckf.2 | 1.17E-29 | 4.245518 | down | -2.178438 | -0.0924974 | n/a | n/a | NM_001258249 |
| uc022ckg.2 | 2.45E-29 | 4.032983 | down | -2.2834322 | -0.271585 | n/a | n/a | NM_001258252 |
| uc022ckj.2 | 9.36E-30 | 4.3056283 | down | -2.2440703 | -0.1378465 | n/a | n/a | NM_001258258 |
| uc022ckn.2 | 1.38E-28 | 4.086316 | down | -2.2574725 | -0.2266717 | n/a | n/a | NM_001258251 |
| uc022ckq.2 | 5.92E-29 | 4.0484033 | down | -2.2933328 | -0.2759796 | n/a | n/a | NM_001258256 |
| uc033csg.1 | 0.03073894 | 3.9783487 | up | -0.381549 | -2.3737187 | n/a | n/a | NR_039688 |
| uc057ukp.1 | 0.01872875 | 3.0890422 | down | -1.0552721 | 0.57188743 | n/a | n/a | NR_073447 |
| uc059mxr.1 | 0.01835862 | 3.3524635 | up | 0.5813638 | -1.1638578 | n/a | n/a | NM_001303234 |
| uc059xwj.1 | 0.03450751 | 3.166916 | down | -2.1777365 | -0.5146579 | n/a | n/a | NM_001291454 |
| uc062qko.1 | 0.00206118 | 4.854856 | up | -1.1580049 | -3.4374332 | n/a | n/a | NR_075089 |
| uc064mda.1 | 0.00944297 | 3.690986 | up | -3.447562 | -5.3315682 | n/a | n/a | NM_001174066 |
| uc065cru.1 | 8.97E-26 | 8.081257 | down | -1.7726135 | 1.2419664 | n/a | n/a | NM_001302552 |
| uc065crv.1 | 6.61E-10 | 4.6437545 | down | -0.9508694 | 1.2644224 | n/a | n/a | NM_001302552 |
| uc065crx.1 | 2.65E-16 | 3.205997 | down | -0.123269 | 1.557504 | n/a | n/a | NM_001302552 |
| uc065crz.1 | 2.67E-20 | 9.097182 | down | -3.7320411 | -0.5466213 | n/a | n/a | NR_047634 |
| uc060pnx.1 | 0.01947117 | 3.4469218 | down | -4.8177285 | -3.03242 | NEDD4L | Homo sapiens neural precursor cell expressed, developmentally down-regulated 4-I | NM_001243960 |
| uc062qgl.1 | 0.0038996 | 3.2986445 | up | -1.9106007 | -3.632474 | PEX5L | Homo sapiens peroxisomal biogenesis factor 5-like (PEX5L), transcript variant 7, mI | NM_001256755 |
| uc059xow.1 | 0.00123614 | 3.5459275 | up | -1.0632253 | -2.8893883 | PKD1L2 | Homo sapiens polycystic kidney disease 1-like 2 (gene/pseudogene) (PKD1L2), trans | NM_001278425 |
| uc062zne.1 | 0.00707994 | 3.4278123 | down | -1.7082833 | 0.06900481 | RP11-420A2: | Homo sapiens uncharacterized LOC100507487 (LOC100507487), long non-coding RI | NR_125882 |
| uc004fqi.4 | 5.80E-08 | 6.858584 | down | 1.8678665 | 4.645777 | RPS4Y1 | Homo sapiens ribosomal protein S4, Y-linked 1 (RPS4Y1), mRNA. (from RefSeq NM | NM_001008 |
| uc065cpc.1 | 5.29E-26 | 56.036827 | down | -2.8105843 | 2.997719 | RPS4Y1 | Homo sapiens ribosomal protein S4, Y-linked 1 (RPS4Y1), mRNA. (from RefSeq NM | NM_001008 |
| uc065cpd.1 | 1.03E-16 | 3.3501081 | down | 0.06841782 | 1.8126254 | RPS4Y1 | Homo sapiens ribosomal protein S4, Y-linked 1 (RPS4Y1), mRNA. (from RefSeq NM | NM_001008 |
| uc061xll.1 | 0.01804181 | 3.123604 | down | -2.845503 | -1.2022915 | SLC12A5 | Homo sapiens solute carrier family 12 (potassium/chloride transporter), member 5 | NM_001134771 |
| uc031tdn.2 | 0.02524371 | 5.191672 | up | -0.6484059 | -3.024605 | SNORD121B | Homo sapiens small nucleolar RNA, C/D box 121B (SNORD121B), small nucleolar R | NR_102370 |
| uc057zln.1 | 0.02050424 | 4.863273 | down | -2.7753856 | -0.493458 | SNORD14B | Homo sapiens small nucleolar RNA, C/D box 14B (SNORD14B), small nucleolar RN | NR_001452 |
| uc060hqf.1 | 0.01399945 | 3.0946639 | down | -2.309882 | -0.6800992 | TOM1L1 | Homo sapiens target of myb1 (chicken)-like 1 (TOM1L1), mRNA. (from RefSeq NM_ | NM_005486 |
| uc065crn.1 | 2.29E-16 | 4.51908 | down | -0.8411754 | 1.3348538 | TTTY15 | Homo sapiens testis-specific transcript, Y-linked 15 (non-protein coding) (TTTY15), | NR_001545 |
| uc061awx.1 | 0.02167169 | 3.129873 | down | -3.7801132 | -2.1340091 | TULP2 | Homo sapiens tubby like protein 2 (TULP2), mRNA. (from RefSeq NM_003323) | NM_003323 |
| uc065crq.1 | 2.53E-11 | 3.331889 | down | -1.6803708 | 0.05596946 | USP9Y | Homo sapiens ubiquitin specific peptidase 9, Y-linked (USP9Y), mRNA. (from RefSe | NM_004654 |
| uc004fsx.3 | 2.21E-29 | 4.0840335 | down | -2.3133934 | -0.2833986 | UTY | Homo sapiens ubiquitously transcribed tetratricopeptide repeat containing, Y-linkec | NM_007125 |
| uc004fsy.4 | 4.22E-27 | 4.5797243 | down | -2.1563802 | 0.03888058 | UTY | Homo sapiens ubiquitously transcribed tetratricopeptide repeat containing, Y-linkec | NM_182659 |
| uc004fsz.3 | 1.70E-21 | 3.3569968 | down | -1.3580545 | 0.38911662 | UTY | Homo sapiens ubiquitously transcribed tetratricopeptide repeat containing, Y-linkec | NM_182660 |
| uc022cjm.2 | 4.60E-29 | 4.0109487 | down | -2.262553 | -0.2586097 | UTY | Homo sapiens ubiquitously transcribed tetratricopeptide repeat containing, Y-linkec | NM_001258266 |
| uc022cjv.2 | 5.10E-28 | 3.8581698 | down | -2.2205687 | -0.2726521 | UTY | Homo sapiens ubiquitously transcribed tetratricopeptide repeat containing, Y-linkec | NM_001258265 |
| uc022cjw.2 | 8.41E-29 | 3.992532 | down | -2.1967497 | -0.1994457 | UTY | Homo sapiens ubiquitously transcribed tetratricopeptide repeat containing, Y-linkec | NM_001258261 |
| uc022ckp.3 | 3.88E-33 | 5.599723 | down | -2.6395357 | -0.1541803 | UTY | Homo sapiens ubiquitously transcribed tetratricopeptide repeat containing, Y-linkec | NM_001258254 |
| uc065csc.1 | 1.17E-07 | 3.8994606 | down | -1.1526278 | 0.8106468 | UTY | Homo sapiens ubiquitously transcribed tetratricopeptide repeat containing, Y-linkec | NM_182660 |
| uc004ebm.3 | 1.55E-29 | 4.762518 | up | 3.2276194 | 0.975895 | XIST | Homo sapiens X inactive specific transcript (non-protein coding) (XIST), long non-co | NR_001564 |
| uc064zym.1 | 5.07E-16 | 3.0539112 | up | 0.8455181 | -0.7651401 | XIST | Homo sapiens X inactive specific transcript (non-protein coding) (XIST), long non-co | NR_001564 |

| | | | | | | | | |
|---|---|---|---|---|---|---|---|---|
| uc064zyn.1 | 4.77E-17 | 3.903899 | up | 0.44827378 | -1.516642 | XIST | Homo sapiens X inactive specific transcript (non-protein coding) (XIST), long non-co | NR_001564 |
| uc064zyo.1 | 1.12E-20 | 4.9645014 | up | 0.9907029 | -1.3209459 | XIST | Homo sapiens X inactive specific transcript (non-protein coding) (XIST), long non-co | NR_001564 |
| uc065cpi.1 | 1.93E-12 | 4.6426497 | down | -1.6081386 | 0.6068098 | ZFY | Homo sapiens zinc finger protein, Y-linked (ZFY), transcript variant 1, mRNA. (from | NM_003411 |

| Supplementary Table 3. | | ANOV analysis of TRACs | | | | Log2 RPKM Expression | | | | |
|---|---|---|---|---|---|---|---|---|---|---|
| **GeneName** | **p** | **Regulation** | **FC (abs)** | **FC** | **Log FC** | **[LOW](norm** | **[MID+](norm** | **Symbol** | **Description** | **RefSeq** |
| uc060kqb.1 | 9.52E-04 | DOWN | 1.4337534 | 1.4337534 | 0.51979685 | -0.185121 | -0.7049179 | SEPTIN9 | Homo sapiens septin 9 (SEPT9), transcript variant 9, mRNA. (from RefSeq NM_001293696) | NM_001293696 |
| uc003wjo.3 | 9.21E-05 | DOWN | 1.2744105 | 1.2744105 | 0.34983003 | 1.0920848 | 0.74225473 | ABCF2 | Homo sapiens ATP-binding cassette, sub-family F (GCN20), member 2 (ABCF2), transcript variant 2, | NM_005692 |
| uc064jjf.1 | 7.85E-04 | DOWN | 1.2906467 | 1.2906467 | 0.3680941 | 0.6617042 | 0.2936101 | ABCF2 | Homo sapiens ATP-binding cassette, sub-family F (GCN20), member 2 (ABCF2), transcript variant 2, | NM_005692 |
| uc057wcy.1 | 8.08E-04 | DOWN | 1.3010219 | 1.3010219 | 0.3796453 | -0.053218 | -0.4328632 | ABLIM1 | Homo sapiens actin binding LIM protein 1 (ABLIM1), transcript variant 1, mRNA. (from RefSeq NM_( | NM_002313 |
| uc057bbq.1 | 4.81E-04 | DOWN | 1.3252088 | 1.3252088 | 0.40621966 | 1.2488762 | 0.84265655 | ACAP3 | Homo sapiens ArfGAP with coiled-coil, ankyrin repeat and PH domains 3 (ACAP3), mRNA. (from Ref! | NM_030649 |
| uc064mff.1 | 9.69E-04 | DOWN | 2.3338773 | 2.3338773 | 1.2227287 | -2.3129778 | -3.5357065 | ADAM32, | Homo sapiens ADAM metallopeptidase domain 32 (ADAM32), mRNA. (from RefSeq NM_145004), | NM_145004 |
| uc002zgr.3 | 6.60E-04 | DOWN | 1.3100791 | 1.3100791 | 0.38965392 | -0.7031987 | -1.0928526 | ADARB1 | Homo sapiens adenosine deaminase, RNA-specific, B1 (ADARB1), transcript variant 3, mRNA. (from | NM_015834 |
| uc059xhj.1 | 6.69E-04 | DOWN | 1.5142472 | 1.5142472 | 0.59860075 | 1.7449799 | 1.1463791 | ADAT1 | Homo sapiens adenosine deaminase, tRNA-specific 1 (ADAT1), transcript variant 1, mRNA. (from Re | NM_012091 |
| uc021xqj.2 | 6.12E-05 | DOWN | 1.7142575 | 1.7142575 | 0.7775838 | -1.9975505 | -2.7751343 | ADH7 | Homo sapiens alcohol dehydrogenase 7 (class IV), mu or sigma polypeptide (ADH7), transcript variar | NM_001166504 |
| uc002bmt.3 | 9.57E-04 | DOWN | 1.2088468 | 1.2088468 | 0.27363142 | 1.1450907 | 0.8714593 | AEN | Homo sapiens apoptosis enhancing nuclease (AEN), mRNA. (from RefSeq NM_022767) | NM_022767 |
| uc062apr.1 | 5.21E-04 | DOWN | 1.3921204 | 1.3921204 | 0.47728395 | 0.42026684 | -0.0570171 | AGPAT3 | Homo sapiens 1-acylglycerol-3-phosphate O-acyltransferase 3 (AGPAT3), transcript variant 2, mRNA | NM_001037553 |
| uc011kox.3 | 7.76E-04 | DOWN | 1.2946298 | 1.2946298 | 0.37253964 | -0.6687162 | -1.0412558 | AHCYL2 | Homo sapiens adenosylhomocysteinase-like 2 (AHCYL2), transcript variant 3, mRNA. (from RefSeq N | NM_001130722 |
| uc064uzz.1 | 8.86E-04 | DOWN | 1.3878013 | 1.3878013 | 0.472801 | -0.4984947 | -0.9712957 | AKAP2 | Homo sapiens A kinase (PRKA) anchor protein 2 (AKAP2), transcript variant 2, mRNA. (from RefSeq I | NM_001136562 |
| uc064ieo.1 | 4.98E-04 | DOWN | 1.260167 | 1.260167 | 0.33361495 | 0.13701719 | -0.1965978 | AKR1B1 | Homo sapiens aldo-keto reductase family 1, member B1 (aldose reductase) (AKR1B1), mRNA. (fron | NM_001628 |
| uc001ukx.3 | 4.25E-04 | DOWN | 1.2933732 | 1.2933732 | 0.37113863 | 1.04623 | 0.6750913 | ANKLE2 | Homo sapiens ankyrin repeat and LEM domain containing 2 (ANKLE2), mRNA. (from RefSeq NM_01! | NM_015114 |
| uc061lxq.1 | 4.91E-04 | DOWN | 1.416466 | 1.416466 | 0.502296 | -0.9948019 | -1.497098 | ANKRD23 | Homo sapiens ankyrin repeat domain 23 (ANKRD23), mRNA. (from RefSeq NM_144994) | NM_144994 |
| uc064mjf.1 | 3.22E-04 | DOWN | 1.3409983 | 1.3409983 | 0.4233074 | -1.183571 | -1.6068784 | AP3M2 | Homo sapiens adaptor-related protein complex 3, mu 2 subunit (AP3M2), transcript variant 2, mRN/ | NM_006803 |
| uc001mdb.4 | 1.54E-04 | DOWN | 1.3240794 | 1.3240794 | 0.40498963 | -0.7239344 | -1.128924 | APBB1 | Homo sapiens amyloid beta (A4) precursor protein-binding, family B, member 1 (Fe65) (APBB1), tra | NM_001164 |
| uc001mdc.3 | 1.14E-04 | DOWN | 1.3491114 | 1.3491114 | 0.43200952 | -0.5606896 | -0.9926991 | APBB1 | Homo sapiens amyloid beta (A4) precursor protein-binding, family B, member 1 (Fe65) (APBB1), tra | NM_145689 |
| uc001mdd.6 | 4.39E-04 | DOWN | 1.3442086 | 1.3442086 | 0.42675704 | -1.2639574 | -1.6907145 | APBB1 | Homo sapiens amyloid beta (A4) precursor protein-binding, family B, member 1 (Fe65) (APBB1), tra | NM_001257323 |
| uc057yjt.1 | 5.44E-04 | DOWN | 1.3164849 | 1.3164849 | 0.396691 | -1.6357287 | -2.0324197 | APBB1 | Homo sapiens amyloid beta (A4) precursor protein-binding, family B, member 1 (Fe65) (APBB1), tra | NM_001257321 |
| uc057yjv.1 | 6.77E-05 | DOWN | 1.3608962 | 1.3608962 | 0.44455707 | -0.5966111 | -1.0411681 | APBB1 | Homo sapiens amyloid beta (A4) precursor protein-binding, family B, member 1 (Fe65) (APBB1), tra | NM_001164 |
| uc057yjz.1 | 1.95E-04 | DOWN | 1.3163289 | 1.3163289 | 0.39652 | -0.7434836 | -1.1400036 | APBB1 | Homo sapiens amyloid beta (A4) precursor protein-binding, family B, member 1 (Fe65) (APBB1), tra | NM_001164 |
| uc057yka.1 | 3.43E-04 | DOWN | 1.3295468 | 1.3295468 | 0.41093457 | -0.5385923 | -0.9495268 | APBB1 | Homo sapiens amyloid beta (A4) precursor protein-binding, family B, member 1 (Fe65) (APBB1), tra | NR_047512 |
| uc057ykb.1 | 4.13E-04 | DOWN | 1.3299803 | 1.3299803 | 0.41140482 | -1.632174 | -2.0435789 | APBB1 | Homo sapiens amyloid beta (A4) precursor protein-binding, family B, member 1 (Fe65) (APBB1), tra | NM_001257321 |
| uc057ykd.1 | 5.06E-05 | DOWN | 1.4381772 | 1.4381772 | 0.52424145 | -1.1580667 | -1.6823082 | APBB1 | Homo sapiens amyloid beta (A4) precursor protein-binding, family B, member 1 (Fe65) (APBB1), tra | NM_001257320 |
| uc061yli.1 | 8.04E-04 | DOWN | 1.5089302 | 1.5089302 | 0.59352607 | -0.3083625 | -0.9018886 | ARFGAP1 | Homo sapiens ADP-ribosylation factor GTPase activating protein 1 (ARFGAP1), transcript variant 1, r | NM_018209 |
| uc065ahz.1 | 1.71E-04 | DOWN | 1.4031242 | 1.4031242 | 0.48864272 | -0.0871896 | -0.5758323 | ARMCX6 | Homo sapiens armadillo repeat containing, X-linked 6 (ARMCX6), transcript variant 3, mRNA. (from | NM_001184768 |
| uc003cgd.4 | 5.36E-04 | DOWN | 4.2413015 | 4.2413015 | 2.084507 | -4.205631 | -6.290138 | ARPP21 | Homo sapiens cAMP-regulated phosphoprotein, 21kDa (ARPP21), transcript variant 7, mRNA. (from | NM_001267618 |
| uc059zrw.1 | 2.96E-04 | DOWN | 1.3435875 | 1.3435875 | 0.4260903 | 1.8983804 | 1.47229 | ATP2A3 | Homo sapiens ATPase, Ca++ transporting, ubiquitous (ATP2A3), transcript variant 7, mRNA. (from R | NM_174958 |
| uc002jfh.4 | 3.81E-04 | DOWN | 1.4512465 | 1.4512465 | 0.5372926 | -0.6264079 | -1.1637005 | AXIN2 | Homo sapiens axin 2 (AXIN2), mRNA. (from RefSeq NM_004655) | NM_004655 |
| uc001ynh.3 | 8.47E-04 | DOWN | 1.3201435 | 1.3201435 | 0.4006947 | 1.134688 | 0.73399335 | BAG5 | Homo sapiens BCL2-associated athanogene 5 (BAG5), transcript variant 1, mRNA. (from RefSeq NM | NM_001015049 |
| uc059ulu.1 | 2.64E-04 | DOWN | 1.9204495 | 1.9204495 | 0.94144404 | -0.0587493 | -1.0001934 | BBS2 | Homo sapiens Bardet-Biedl syndrome 2 (BBS2), mRNA. (from RefSeq NM_031885) | NM_031885 |
| uc061wwa.1 | 1.26E-04 | DOWN | 1.4126246 | 1.4126246 | 0.49837813 | 0.24412708 | -0.2542511 | BLCAP | Homo sapiens bladder cancer associated protein (BLCAP), transcript variant 2, mRNA. (from RefSeq | NM_001167820 |
| uc061wwb.1 | 8.95E-04 | DOWN | 1.3646924 | 1.3646924 | 0.44857585 | 0.1648186 | -0.2837572 | BLCAP | Homo sapiens bladder cancer associated protein (BLCAP), transcript variant 2, mRNA. (from RefSeq | NM_001167820 |
| uc064kgd.1 | 7.42E-04 | DOWN | 1.3937876 | 1.3937876 | 0.47901073 | 0.83950424 | 0.36049345 | BLK | Homo sapiens BLK proto-oncogene, Src family tyrosine kinase (BLK), mRNA. (from RefSeq NM_0017 | NM_001715 |
| uc063hjk.1 | 9.02E-04 | DOWN | 1.332296 | 1.332296 | 0.41391465 | -0.6734464 | -1.087361 | BRD8 | Homo sapiens bromodomain containing 8 (BRD8), transcript variant 6, mRNA. (from RefSeq NM_00 | NM_001300962 |
| uc003dzb.5 | 4.12E-04 | DOWN | 1.7705259 | 1.7705259 | 0.824178 | 0.9693076 | 0.14512964 | BTLA | Homo sapiens B and T lymphocyte associated (BTLA), transcript variant 2, mRNA. (from RefSeq NM | NM_001085357 |
| uc058bon.1 | 2.60E-04 | DOWN | 1.3815451 | 1.3815451 | 0.4662826 | 1.6815842 | 1.2153016 | C11orf31 | Homo sapiens chromosome 11 open reading frame 31 (C11orf31), mRNA. (from RefSeq NM_17074 | NM_170746 |
| uc058usc.1 | 9.80E-04 | DOWN | 1.4553193 | 1.4553193 | 0.5413357 | -0.7864534 | -1.3277892 | C12orf65 | Homo sapiens chromosome 12 open reading frame 65 (C12orf65), transcript variant 1, mRNA. (from | NM_152269 |
| uc059tos.1 | 3.95E-04 | DOWN | 1.3457203 | 1.3457203 | 0.42837858 | -0.768739 | -1.1971176 | C16orf58 | Homo sapiens chromosome 16 open reading frame 58 (C16orf58), mRNA. (from RefSeq NM_02274 | NM_022744 |
| uc021tna.1 | 3.01E-04 | DOWN | 1.3925934 | 1.3925934 | 0.47777405 | 0.6382703 | 0.16049625 | C17orf97 | Homo sapiens chromosome 17 open reading frame 97 (C17orf97), mRNA. (from RefSeq NM_00101 | NM_001013672 |
| uc002rji.4 | 8.56E-04 | DOWN | 1.2446817 | 1.2446817 | 0.31577685 | 0.35971102 | 0.04393421 | CAD | Homo sapiens carbamoyl-phosphate synthetase 2, aspartate transcarbamylase, and dihydroorotase | NM_004341 |
| uc063fyf.1 | 6.94E-04 | DOWN | 1.3283268 | 1.3283268 | 0.40961015 | 0.848183 | 0.4385728 | CAMK4 | Homo sapiens calcium/calmodulin-dependent protein kinase IV (CAMK4), mRNA. (from RefSeq NM_ | NM_001744 |
| uc010nbg.4 | 8.59E-04 | DOWN | 1.1643348 | 1.1643348 | 0.21950592 | 0.099172 | -0.1203339 | CAMSAP1 | Homo sapiens calmodulin regulated spectrin-associated protein 1 (CAMSAP1), mRNA. (from RefSeq | NM_015447 |
| uc064rtg.1 | 4.80E-04 | DOWN | 1.9413694 | 1.9413694 | 0.95707464 | -3.2309866 | -4.188061 | CBWD1 | Homo sapiens COBW domain containing 1 (CBWD1), transcript variant 1, mRNA. (from RefSeq NM_ | NM_018491 |
| uc021wxn.3 | 6.08E-04 | DOWN | 1.3152108 | 1.3152108 | 0.39529407 | -0.3335028 | -0.7287969 | CCDC51 | Homo sapiens coiled-coil domain containing 51 (CCDC51), transcript variant 3, mRNA. (from RefSeq | NM_001256965 |
| uc010aty.4 | 4.81E-04 | DOWN | 1.3128444 | 1.3128444 | 0.39269593 | 2.970372 | 2.577676 | CCDC88C | Homo sapiens coiled-coil domain containing 88C (CCDC88C), mRNA. (from RefSeq NM_001080414) | NM_001080414 |
| uc058mhe.1 | 9.03E-04 | DOWN | 1.414445 | 1.414445 | 0.5002361 | 1.3163532 | 0.8161171 | CCDC91 | Homo sapiens coiled-coil domain containing 91 (CCDC91), mRNA. (from RefSeq NM_018318) | NM_018318 |
| uc063myg.1 | 4.14E-04 | DOWN | 2.0276597 | 2.0276597 | 1.0198154 | -5.231518 | -6.251333 | CCHCR1, | Homo sapiens coiled-coil alpha-helical rod protein 1 (CCHCR1), transcript variant 3, mRNA. (from Re | NM_019052 |
| uc060xci.1 | 5.68E-04 | DOWN | 1.5151955 | 1.5151955 | 0.59950393 | -0.6206064 | -1.2201103 | CD22 | Homo sapiens CD22 molecule (CD22), transcript variant 5, mRNA. (from RefSeq NM_001278417) | NM_001278417 |

| | | | | | | | | | | |
|---|---|---|---|---|---|---|---|---|---|---|
| uc064bww.1 | 7.87E-04 | DOWN | 1.4893333 | 1.4893333 | 0.5746666 | -0.4312408 | -1.0059074 | CDCA7L | Homo sapiens cell division cycle associated 7-like (CDCA7L), transcript variant 3, mRNA. (from RefSe | NM_001127371 |
| uc064bwy.1 | 1.07E-04 | DOWN | 1.5699714 | 1.5699714 | 0.6507383 | -0.3568905 | -1.0076288 | CDCA7L | Homo sapiens cell division cycle associated 7-like (CDCA7L), transcript variant 2, mRNA. (from RefSe | NM_001127370 |
| uc059xrj.1 | 1.26E-04 | DOWN | 5.5537534 | 5.5537534 | 2.473463 | -1.2926244 | -3.7660873 | CDH13 | Homo sapiens cadherin 13 (CDH13), transcript variant 5, mRNA. (from RefSeq NM_001220491) | NM_001220491 |
| uc060awd.1 | 8.00E-04 | DOWN | 1.6806908 | 1.6806908 | 0.7490543 | 2.506842 | 1.7577876 | CHD3 | Homo sapiens chromodomain helicase DNA binding protein 3 (CHD3), transcript variant 2, mRNA. (fr | NM_005852 |
| uc064ldx.1 | 7.20E-04 | DOWN | 1.3877572 | 1.3877572 | 0.47275516 | 0.6397034 | 0.16694826 | CHMP7 | Homo sapiens charged multivesicular body protein 7 (CHMP7), mRNA. (from RefSeq NM_152272) | NM_152272 |
| uc059ljk.1 | 5.54E-04 | DOWN | 1.3671507 | 1.3671507 | 0.45117223 | 0.8071874 | 0.35601518 | CLK3 | Homo sapiens CDC-like kinase 3 (CLK3), transcript variant 2, mRNA. (from RefSeq NM_003992) | NM_003992 |
| uc064kms.1 | 7.10E-04 | DOWN | 1.3634907 | 1.3634907 | 0.44730484 | 1.4489573 | 1.0016525 | CNOT7 | Homo sapiens CCR4-NOT transcription complex, subunit 7 (CNOT7), transcript variant 2, mRNA. (fro | NM_054026 |
| uc063jat.1 | 2.72E-04 | DOWN | 1.5294986 | 1.5294986 | 0.61305875 | 0.32510754 | -0.2879512 | CNOT8 | Homo sapiens CCR4-NOT transcription complex, subunit 8 (CNOT8), transcript variant 3, mRNA. (fro | NM_001301074 |
| uc057egb.1 | 5.95E-04 | DOWN | 5.4626527 | 5.4626527 | 2.4496017 | -1.4775615 | -3.9271631 | COL16A1 | Homo sapiens collagen, type XVI, alpha 1 (COL16A1), mRNA. (from RefSeq NM_001856) | NM_001856 |
| uc064rqz.1 | 1.73E-04 | DOWN | 1.5200763 | 1.5200763 | 0.60414374 | 0.6395183 | 0.03537459 | COMMD5 | Homo sapiens COMM domain containing 5 (COMMD5), transcript variant 3, mRNA. (from RefSeq NI | NM_001081004 |
| uc064ofx.1 | 5.48E-04 | DOWN | 1.6919246 | 1.6919246 | 0.75866526 | 0.70704895 | -0.0516163 | CPNE3 | Homo sapiens copine III (CPNE3), mRNA. (from RefSeq NM_003909) | NM_003909 |
| uc062lwg.1 | 1.45E-04 | DOWN | 1.4263507 | 1.4263507 | 0.51232874 | 1.625692 | 1.1133633 | CPOX | Homo sapiens coproporphyrinogen oxidase (CPOX), mRNA. (from RefSeq NM_000097) | NM_000097 |
| uc009voy.2 | 6.80E-04 | DOWN | 1.4515594 | 1.4515594 | 0.5376036 | 2.3564265 | 1.8188229 | CROCC | Homo sapiens ciliary rootlet coiled-coil, rootletin (CROCC), mRNA. (from RefSeq NM_014675) | NM_014675 |
| uc009ykw.4 | 3.53E-04 | DOWN | 1.2723478 | 1.2723478 | 0.34749308 | 0.12051815 | -0.226975 | CRY2 | Homo sapiens cryptochrome circadian clock 2 (CRY2), transcript variant 2, mRNA. (from RefSeq NM_ | NM_001127457 |
| uc010rgn.2 | 1.46E-04 | DOWN | 1.19535 | 1.19535 | 0.25743315 | 0.8755804 | 0.6181472 | CRY2 | Homo sapiens cryptochrome circadian clock 2 (CRY2), transcript variant 1, mRNA. (from RefSeq NM_ | NM_021117 |
| uc058ato.1 | 1.38E-04 | DOWN | 1.1960957 | 1.1960957 | 0.25833282 | 0.8766598 | 0.618327 | CRY2 | Homo sapiens cryptochrome circadian clock 2 (CRY2), transcript variant 1, mRNA. (from RefSeq NM_ | NM_021117 |
| uc062ukk.1 | 8.32E-04 | DOWN | 1.3743054 | 1.3743054 | 0.4587026 | 0.43223295 | -0.0264697 | CTBP1 | Homo sapiens C-terminal binding protein 1 (CTBP1), transcript variant 2, mRNA. (from RefSeq NM_( | NM_001012614 |
| uc059zqi.1 | 2.29E-04 | DOWN | 1.7476925 | 1.7476925 | 0.80545133 | -0.2479629 | -1.0534142 | CTNS | Homo sapiens cystinosin, lysosomal cystine transporter (CTNS), transcript variant 2, mRNA. (from R | NM_004937 |
| uc057jkc.1 | 3.39E-04 | DOWN | 1.4018576 | 1.4018576 | 0.48733982 | -0.0166596 | -0.5039994 | CTTNBP2NL | Homo sapiens CTTNBP2 N-terminal like (CTTNBP2NL), mRNA. (from RefSeq NM_018704) | NM_018704 |
| uc002leq.5 | 4.03E-04 | DOWN | 1.2371509 | 1.2371509 | 0.3070215 | 0.2981659 | -0.0088556 | CXXC1 | Homo sapiens CXXC finger protein 1 (CXXC1), transcript variant 2, mRNA. (from RefSeq NM_014593) | NM_014593 |
| uc063hsz.1 | 9.93E-04 | DOWN | 1.2923493 | 1.2923493 | 0.3699961 | -0.3193838 | -0.6893799 | CXXC5 | Homo sapiens CXXC finger protein 5 (CXXC5), mRNA. (from RefSeq NM_016463) | NM_016463 |
| uc058cbb.1 | 3.72E-04 | DOWN | 1.5803254 | 1.5803254 | 0.66022164 | -0.0946591 | -0.7548807 | CYB561A3 | Homo sapiens cytochrome b561 family, member A3 (CYB561A3), transcript variant 4, mRNA. (from | NM_001300763 |
| uc059zsi.1 | 7.55E-04 | DOWN | 1.3452644 | 1.3452644 | 0.4278898 | -0.7224397 | -1.1503295 | CYB5D2 | Homo sapiens cytochrome b5 domain containing 2 (CYB5D2), transcript variant 2, mRNA. (from RefS | NM_001254755 |
| uc061otq.1 | 1.87E-05 | DOWN | 1.4315364 | 1.4315364 | 0.51756436 | 0.38363758 | -0.1339269 | CYTIP | Homo sapiens cytohesin 1 interacting protein (CYTIP), mRNA. (from RefSeq NM_004288) | NM_004288 |
| uc060rtj.1 | 6.73E-04 | DOWN | 1.6379657 | 1.6379657 | 0.7119051 | 2.3080022 | 1.5960971 | DAPK3 | Homo sapiens death-associated protein kinase 3 (DAPK3), mRNA. (from RefSeq NM_001348) | NM_001348 |
| uc002tux.2 | 8.79E-04 | DOWN | 1.2755452 | 1.2755452 | 0.35111406 | 3.121397 | 2.770283 | DARS | Homo sapiens aspartyl-tRNA synthetase (DARS), transcript variant 1, mRNA. (from RefSeq NM_001 | NM_001349 |
| uc058mlq.1 | 9.58E-04 | DOWN | 1.923227 | 1.923227 | 0.943529 | -1.1854318 | -2.1289608 | DDX11 | Homo sapiens DEAD/H (Asp-Glu-Ala-Asp/His) box helicase 11 (DDX11), transcript variant 5, mRNA. ( | NM_001257145 |
| uc057jjs.1 | 7.91E-04 | DOWN | 1.3948647 | 1.3948647 | 0.48012516 | 0.2522162 | -0.227909 | DDX20 | Homo sapiens DEAD (Asp-Glu-Ala-Asp) box polypeptide 20 (DDX20), mRNA. (from RefSeq NM_0072 | NM_007204 |
| uc058utt.1 | 4.44E-04 | DOWN | 1.3347564 | 1.3347564 | 0.41657645 | -1.2244805 | -1.6410569 | DDX55 | Homo sapiens DEAD (Asp-Glu-Ala-Asp) box polypeptide 55 (DDX55), mRNA. (from RefSeq NM_0209 | NM_020936 |
| uc021wlp.2 | 1.54E-05 | DOWN | 1.4976513 | 1.4976513 | 0.5827018 | 0.42528155 | -0.1574202 | DGCR8 | Homo sapiens DGCR8 microprocessor complex subunit (DGCR8), transcript variant 2, mRNA. (from F | NM_001190326 |
| uc058pdr.1 | 8.60E-04 | DOWN | 1.8234607 | 1.8234607 | 0.8666791 | 0.07406825 | -0.7926108 | DGKA | Homo sapiens diacylglycerol kinase, alpha 80kDa (DGKA), transcript variant 4, mRNA. (from RefSeq | NM_201554 |
| uc058pdy.1 | 1.65E-04 | DOWN | 1.4035074 | 1.4035074 | 0.48903662 | 0.18466794 | -0.3043687 | DGKA | Homo sapiens diacylglycerol kinase, alpha 80kDa (DGKA), transcript variant 4, mRNA. (from RefSeq | NM_201554 |
| uc060uba.1 | 4.56E-04 | DOWN | 1.4090039 | 1.4090039 | 0.49467555 | 0.12977083 | -0.3649048 | DHPS | Homo sapiens deoxyhypusine synthase (DHPS), transcript variant 1, mRNA. (from RefSeq NM_00193 | NM_001930 |
| uc062jfe.1 | 1.41E-04 | DOWN | 1.5565609 | 1.5565609 | 0.638362 | 0.33597872 | -0.3023833 | DHX30 | Homo sapiens DEAH (Asp-Glu-Ala-His) box helicase 30 (DHX30), transcript variant 1, mRNA. (from R | NM_138615 |
| uc058xkw.1 | 6.03E-04 | DOWN | 1.4960788 | 1.4960788 | 0.5811862 | 0.79466057 | 0.21347432 | DIS3 | Homo sapiens DIS3 exosome endoribonuclease and 3'-5' exoribonuclease (DIS3), transcript variant 1 | NM_014953 |
| uc061cxr.1 | 4.42E-04 | DOWN | 3.4806626 | 3.4806626 | 1.799362 | -2.874132 | -4.673494 | DNAAF3 | Homo sapiens dynein, axonemal, assembly factor 3 (DNAAF3), transcript variant 1, mRNA. (from Re | NM_001256714 |
| uc062eiv.1 | 2.90E-04 | DOWN | 1.9544363 | 1.9544363 | 0.9667525 | 0.49695006 | -0.4698025 | DNAL4 | Homo sapiens dynein, axonemal, light chain 4 (DNAL4), mRNA. (from RefSeq NM_005740) | NM_005740 |
| uc059zkr.1 | 2.37E-04 | DOWN | 1.7925972 | 1.7925972 | 0.8420513 | 1.6935285 | 0.8514772 | DPH1 | Homo sapiens diphthamide biosynthesis 1 (DPH1), mRNA. (from RefSeq NM_001383) | NM_001383 |
| uc063iju.1 | 3.26E-04 | DOWN | 3.0620632 | 3.0620632 | 1.6145041 | -2.5977495 | -4.2122536 | DPYSL3 | Homo sapiens dihydropyrimidinase-like 3 (DPYSL3), transcript variant 2, mRNA. (from RefSeq NM_0 | NM_001387 |
| uc002kwk.5 | 4.97E-04 | UP | 1.5694616 | -1.5694616 | -0.6502697 | 2.0402553 | 2.690525 | DSC2 | Homo sapiens desmocollin 2 (DSC2), transcript variant Dsc2b, mRNA. (from RefSeq NM_004949) | NM_004949 |
| uc059jbx.1 | 9.32E-04 | DOWN | 2.828315 | 2.828315 | 1.4999428 | -0.5355427 | -2.0354855 | DTWD1 | Homo sapiens DTW domain containing 1 (DTWD1), transcript variant 2, mRNA. (from RefSeq NM_0 | NM_001144955 |
| uc060sdo.1 | 9.03E-04 | DOWN | 1.3031578 | 1.3031578 | 0.3820118 | 0.08413836 | -0.2978735 | DUS3L | Homo sapiens dihydrouridine synthase 3-like (S. cerevisiae) (DUS3L), transcript variant 1, mRNA. (fr | NM_020175 |
| uc060sds.1 | 2.85E-04 | DOWN | 1.4717858 | 1.4717858 | 0.5575677 | 0.514877 | -0.0426907 | DUS3L | Homo sapiens dihydrouridine synthase 3-like (S. cerevisiae) (DUS3L), transcript variant 1, mRNA. (fr | NM_020175 |
| uc010wnn.2 | 2.46E-04 | DOWN | 1.2284846 | 1.2284846 | 0.2968798 | 3.522628 | 3.2257483 | DYNLL2 | Homo sapiens dynein, light chain, LC8-type 2 (DYNLL2), mRNA. (from RefSeq NM_080677) | NM_080677 |
| uc060ttv.1 | 6.18E-04 | DOWN | 1.3899735 | 1.3899735 | 0.4750574 | 0.64705426 | 0.1719969 | ECSIT | Homo sapiens ECSIT signalling integrator (ECSIT), transcript variant 3, mRNA. (from RefSeq NM_00: | NM_001142465 |
| uc059fpj.1 | 7.23E-04 | DOWN | 1.3500446 | 1.3500446 | 0.43300706 | 0.6690862 | 0.23607916 | EIF5 | Homo sapiens eukaryotic translation initiation factor 5 (EIF5), transcript variant 1, mRNA. (from Ref | NM_001969 |
| uc064eht.1 | 6.55E-04 | DOWN | 3.4123712 | 3.4123712 | 1.7707746 | -2.9005659 | -4.6713405 | ELN | Homo sapiens elastin (ELN), transcript variant 3, mRNA. (from RefSeq NM_001081753) | NM_001081753 |
| uc057wfo.1 | 8.12E-04 | DOWN | 2.9038887 | 2.9038887 | 1.5379862 | -0.2584959 | -1.7964821 | EMX2OS | Homo sapiens EMX2 opposite strand/antisense RNA (EMX2OS), long non-coding RNA. (from RefSeq | NR_002791 |
| uc062xsy.1 | 9.11E-04 | DOWN | 1.4225762 | 1.4225762 | 0.50850594 | 1.066101 | 0.557595 | ENOPH1 | Homo sapiens enolase-phosphatase 1 (ENOPH1), transcript variant 1, mRNA. (from RefSeq NM_021 | NM_021204 |
| uc062xsz.1 | 9.13E-04 | DOWN | 1.2292796 | 1.2292796 | 0.29781315 | 0.61124355 | 0.31343034 | ENOPH1 | Homo sapiens enolase-phosphatase 1 (ENOPH1), transcript variant 2, mRNA. (from RefSeq NM_001 | NM_001292017 |
| uc060mtq.1 | 5.20E-04 | DOWN | 1.3539106 | 1.3539106 | 0.43713245 | 0.26639283 | -0.1707396 | ENOSF1 | Homo sapiens enolase superfamily member 1 (ENOSF1), transcript variant 2, mRNA. (from RefSeq I | NM_001126123 |
| uc065bcs.1 | 4.02E-04 | DOWN | 1.4150074 | 1.4150074 | 0.50080955 | -0.0309631 | -0.5317727 | ENOX2 | Homo sapiens ecto-NOX disulfide-thiol exchanger 2 (ENOX2), transcript variant 1, mRNA. (from Ref: | NM_006375 |
| uc058vib.1 | 7.03E-04 | DOWN | 1.2665782 | 1.2665782 | 0.34093615 | 0.23307593 | -0.1078602 | EP400 | Homo sapiens E1A binding protein p400 (EP400), mRNA. (from RefSeq NM_015409) | NM_015409 |

| | | | | | | | | | | |
|---|---|---|---|---|---|---|---|---|---|---|
| uc063rjd.1 | 3.70E-04 | DOWN | 1.5517591 | 1.5517591 | 0.63390464 | -1.7924179 | -2.4263225 | EPB41L2 | Homo sapiens erythrocyte membrane protein band 4.1-like 2 (EPB41L2), transcript variant 2, mRNA. | NM_001135555 |
| uc064iwe.1 | 3.28E-04 | DOWN | 1.3043476 | 1.3043476 | 0.3833284 | 0.30906773 | -0.0742608 | EPHA1 | Homo sapiens EPH receptor A1 (EPHA1), mRNA. (from RefSeq NM_005232) | NM_005232 |
| uc060uys.1 | 3.92E-04 | DOWN | 1.2193388 | 1.2193388 | 0.28609902 | 1.4997584 | 1.2136594 | EPS15L1 | Homo sapiens epidermal growth factor receptor pathway substrate 15-like 1 (EPS15L1), transcript va | NM_001258375 |
| uc060vkb.1 | 1.79E-04 | DOWN | 1.4217726 | 1.4217726 | 0.5076907 | 0.02209011 | -0.4856006 | FAM129C | Homo sapiens family with sequence similarity 129, member C (FAM129C), transcript variant 2, mRN | NM_001098524 |
| uc060vkc.1 | 4.39E-04 | DOWN | 1.3720957 | 1.3720957 | 0.4563811 | -0.1605814 | -0.6169626 | FAM129C | Homo sapiens family with sequence similarity 129, member C (FAM129C), transcript variant 2, mRN | NM_001098524 |
| uc057bod.1 | 4.52E-05 | DOWN | 4.2857165 | 4.2857165 | 2.0995364 | -4.4922795 | -6.591816 | FAM213B, | Homo sapiens family with sequence similarity 213, member B (FAM213B), transcript variant 2, mRN | NM_152371 |
| uc061fvb.1 | 4.52E-05 | DOWN | 4.2857165 | 4.2857165 | 2.0995364 | -4.4922795 | -6.591816 | FAM213B, | Homo sapiens family with sequence similarity 213, member B (FAM213B), transcript variant 2, mRN | NM_152371 |
| uc064tfh.1 | 2.82E-04 | DOWN | 2.5153918 | 2.5153918 | 1.3307831 | -2.90459 | -4.235373 | FAM95B1, | Homo sapiens family with sequence similarity 95, member B1 (FAM95B1), long non-coding RNA. (fr | NR_026759 |
| uc002rpa.2 | 8.82E-04 | DOWN | 1.2223758 | 1.2223758 | 0.2896878 | 0.0637974 | -0.2258904 | FAM98A | Homo sapiens family with sequence similarity 98, member A (FAM98A), transcript variant 1, mRNA | NM_015475 |
| uc010jnv.2 | 9.66E-04 | DOWN | 1.2734643 | 1.2734643 | 0.34875855 | 0.6780751 | 0.32931653 | FARS2 | Homo sapiens phenylalanyl-tRNA synthetase 2, mitochondrial (FARS2), mRNA. (from RefSeq NM_0( | NM_006567 |
| uc060lwd.1 | 4.84E-04 | DOWN | 1.5184833 | 1.5184833 | 0.60263103 | 0.78219837 | 0.17956735 | FASN | Homo sapiens fatty acid synthase (FASN), mRNA. (from RefSeq NM_004104) | NM_004104 |
| uc060ynr.1 | 6.15E-04 | DOWN | 1.4972541 | 1.4972541 | 0.5823191 | 1.4468273 | 0.86450815 | FBL | Homo sapiens fibrillarin (FBL), mRNA. (from RefSeq NM_001436) | NM_001436 |
| uc058ttr.1 | 4.78E-04 | DOWN | 1.3824271 | 1.3824271 | 0.4672034 | 0.706743 | 0.23953962 | FBXO21 | Homo sapiens F-box protein 21 (FBXO21), transcript variant 1, mRNA. (from RefSeq NM_033624) | NM_033624 |
| uc060sqj.1 | 6.55E-04 | DOWN | 1.3777107 | 1.3777107 | 0.46227297 | 0.07015614 | -0.3921169 | FCER2 | Homo sapiens Fc fragment of IgE, low affinity II, receptor for (CD23) (FCER2), transcript variant 1, m | NM_002002 |
| uc060sqm.1 | 5.48E-04 | DOWN | 1.4911617 | 1.4911617 | 0.5764367 | 0.11143559 | -0.4650012 | FCER2 | Homo sapiens Fc fragment of IgE, low affinity II, receptor for (CD23) (FCER2), transcript variant 1, m | NM_002002 |
| uc058fep.1 | 1.75E-04 | DOWN | 1.4564477 | 1.4564477 | 0.5424539 | 0.4302286 | -0.1122253 | FCHSD2 | Homo sapiens FCH and double SH3 domains 2 (FCHSD2), mRNA. (from RefSeq NM_014824) | NM_014824 |
| uc064glm.1 | 1.00E-04 | DOWN | 1.4015203 | 1.4015203 | 0.4869926 | 1.3968903 | 0.90989774 | FIS1 | Homo sapiens fission 1 (mitochondrial outer membrane) homolog (S. cerevisiae) (FIS1), mRNA. (fro | NM_016068 |
| uc003djj.3 | 2.21E-04 | DOWN | 1.2875259 | 1.2875259 | 0.36460143 | -0.1843731 | -0.5489746 | FLNB | Homo sapiens filamin B, beta (FLNB), transcript variant 2, mRNA. (from RefSeq NM_001457) | NM_001457 |
| uc003djk.3 | 2.56E-04 | DOWN | 1.2852204 | 1.2852204 | 0.36201575 | -0.181928 | -0.5439437 | FLNB | Homo sapiens filamin B, beta (FLNB), transcript variant 3, mRNA. (from RefSeq NM_001164318) | NM_001164318 |
| uc003djm.3 | 9.95E-04 | DOWN | 1.2445667 | 1.2445667 | 0.31564352 | -0.159998 | -0.4756416 | FLNB | Homo sapiens filamin B, beta (FLNB), transcript variant 4, mRNA. (from RefSeq NM_001164319) | NM_001164319 |
| uc010hne.3 | 5.21E-04 | DOWN | 1.3080819 | 1.3080819 | 0.38745284 | -0.1450327 | -0.5324855 | FLNB | Homo sapiens filamin B, beta (FLNB), transcript variant 1, mRNA. (from RefSeq NM_001164317) | NM_001164317 |
| uc010hnf.3 | 2.03E-04 | DOWN | 1.29133 | 1.29133 | 0.3688577 | -0.1743539 | -0.5432116 | FLNB | Homo sapiens filamin B, beta (FLNB), transcript variant 4, mRNA. (from RefSeq NM_001164319) | NM_001164319 |
| uc062kyo.1 | 5.42E-04 | DOWN | 1.1885079 | 1.1885079 | 0.24915151 | -0.0901803 | -0.3393317 | FLNB | Homo sapiens filamin B, beta (FLNB), transcript variant 4, mRNA. (from RefSeq NM_001164319) | NM_001164319 |
| uc059prq.1 | 8.53E-04 | DOWN | 1.2842814 | 1.2842814 | 0.36096132 | -0.7828972 | -1.1438586 | FLYWCH1 | Homo sapiens FLYWCH-type zinc finger 1 (FLYWCH1), transcript variant 1, mRNA. (from RefSeq NM | NM_032296 |
| uc064mnv.1 | 7.62E-04 | DOWN | 1.3168805 | 1.3168805 | 0.39712438 | 0.43809152 | 0.0409671 | FNTA | Homo sapiens farnesyltransferase, CAAX box, alpha (FNTA), transcript variant 1, mRNA. (from RefSe | NM_002027 |
| uc064vxp.1 | 8.95E-04 | DOWN | 1.3649472 | 1.3649472 | 0.44884515 | -0.9141061 | -1.3629512 | FPGS | Homo sapiens folylpolyglutamate synthase (FPGS), transcript variant 1, mRNA. (from RefSeq NM_0 | NM_004957 |
| uc064vxr.1 | 2.34E-04 | DOWN | 1.4879673 | 1.4879673 | 0.57334274 | -0.9706222 | -1.543965 | FPGS | Homo sapiens folylpolyglutamate synthase (FPGS), transcript variant 1, mRNA. (from RefSeq NM_0 | NM_004957 |
| uc058djl.1 | 6.44E-04 | DOWN | 1.4106445 | 1.4106445 | 0.4963545 | 0.21383233 | -0.2825221 | FRMD8 | Homo sapiens FERM domain containing 8 (FRMD8), transcript variant 1, mRNA. (from RefSeq NM_( | NM_031904 |
| uc058djm.1 | 3.80E-04 | DOWN | 1.4113189 | 1.4113189 | 0.497044 | 0.4372523 | -0.0597917 | FRMD8 | Homo sapiens FERM domain containing 8 (FRMD8), transcript variant 2, mRNA. (from RefSeq NM_( | NM_001300832 |
| uc060atq.1 | 1.75E-04 | DOWN | 1.3747438 | 1.3747438 | 0.4591628 | 2.0954998 | 1.6363369 | FXR2 | Homo sapiens fragile X mental retardation, autosomal homolog 2 (FXR2), mRNA. (from RefSeq NM | NM_004860 |
| uc062daw.1 | 6.98E-04 | DOWN | 1.2820716 | 1.2820716 | 0.35847682 | -1.822708 | -2.1811848 | GATSL3 | Homo sapiens GATS protein-like 3 (GATSL3), mRNA. (from RefSeq NM_001037666) | NM_001037666 |
| uc062dbe.1 | 8.67E-04 | DOWN | 2.6110659 | 2.6110659 | 1.3846388 | -0.7264488 | -2.1110876 | GATSL3 | Homo sapiens GATS protein-like 3 (GATSL3), mRNA. (from RefSeq NM_001037666) | NM_001037666 |
| uc058tzd.1 | 5.97E-04 | DOWN | 1.3484395 | 1.3484395 | 0.43129075 | 0.9592147 | 0.5279239 | GCN1L1 | Homo sapiens GCN1 general control of amino-acid synthesis 1-like 1 (yeast) (GCN1L1), mRNA. (from | NM_006836 |
| uc063ymk.1 | 9.71E-04 | DOWN | 1.2795461 | 1.2795461 | 0.35563216 | -1.1937543 | -1.5493865 | GNL1, | Homo sapiens guanine nucleotide binding protein-like 1 (GNL1), mRNA. (from RefSeq NM_005275), | NM_005275 |
| uc002whp.2 | 9.74E-04 | DOWN | 3.1071494 | 3.1071494 | 1.6355916 | -1.011351 | -2.6469426 | GNRH2 | Homo sapiens gonadotropin-releasing hormone 2 (GNRH2), transcript variant 3, mRNA. (from RefSe | NM_178331 |
| uc057dtw.1 | 1.33E-04 | DOWN | 1.5824472 | 1.5824472 | 0.66215736 | -0.1753319 | -0.8374892 | GPATCH3 | Homo sapiens G patch domain containing 3 (GPATCH3), mRNA. (from RefSeq NM_022078) | NM_022078 |
| uc063ixf.1 | 3.28E-04 | DOWN | 2.7667317 | 2.7667317 | 1.4681828 | -3.18731 | -4.655493 | GRIA1 | Homo sapiens glutamate receptor, ionotropic, AMPA 1 (GRIA1), transcript variant 7, mRNA. (from R | NM_001258023 |
| uc057idf.1 | 7.14E-04 | DOWN | 1.5648838 | 1.5648838 | 0.6460556 | 0.6933504 | 0.04729484 | GTF2B | Homo sapiens general transcription factor IIB (GTF2B), mRNA. (from RefSeq NM_001514) | NM_001514 |
| uc058waz.1 | 9.32E-04 | DOWN | 1.4175823 | 1.4175823 | 0.50343245 | 1.590342 | 1.0869095 | GTF3A | Homo sapiens general transcription factor IIIA (GTF3A), mRNA. (from RefSeq NM_002097) | NM_002097 |
| uc064wsm.1 | 5.19E-04 | DOWN | 1.3935853 | 1.3935853 | 0.47880134 | 0.7353887 | 0.25658733 | GTF3C4 | Homo sapiens general transcription factor IIIC, polypeptide 4, 90kDa (GTF3C4), mRNA. (from RefSec | NM_012204 |
| uc004cci.5 | 7.15E-04 | DOWN | 1.2272269 | 1.2272269 | 0.29540196 | 0.06520756 | -0.2301944 | GTF3C5 | Homo sapiens general transcription factor IIIC, polypeptide 5, 63kDa (GTF3C5), transcript variant 2, r | NM_012087 |
| uc033dju.2 | 4.19E-04 | DOWN | 1.2532845 | 1.2532845 | 0.3257139 | -0.0266291 | -0.352343 | GTF3C5 | Homo sapiens general transcription factor IIIC, polypeptide 5, 63kDa (GTF3C5), transcript variant 3, r | NM_001286709 |
| uc057reu.1 | 8.73E-04 | DOWN | 1.4814469 | 1.4814469 | 0.5670069 | 1.2631001 | 0.69609326 | GTPBP4 | Homo sapiens GTP binding protein 4 (GTPBP4), mRNA. (from RefSeq NM_012341) | NM_012341 |
| uc062kga.1 | 5.79E-04 | DOWN | 1.3277055 | 1.3277055 | 0.40893516 | -1.03673 | -1.4456652 | HEMK1 | Homo sapiens HemK methyltransferase family member 1 (HEMK1), mRNA. (from RefSeq NM_0161 | NM_016173 |
| uc061aff.1 | 4.05E-04 | DOWN | 5.606754 | 5.606754 | 2.4871657 | -2.5747898 | -5.0619555 | HIF3A | Homo sapiens hypoxia inducible factor 3, alpha subunit (HIF3A), transcript variant 4, mRNA. (from R | NM_152796 |
| uc001udj.2 | 3.14E-04 | DOWN | 1.3390192 | 1.3390192 | 0.4211766 | 1.4725891 | 1.0514125 | HIP1R | Homo sapiens huntingtin interacting protein 1 related (HIP1R), transcript variant 1, mRNA. (from Re | NM_003959 |
| uc058uor.1 | 1.81E-04 | DOWN | 1.3555408 | 1.3555408 | 0.4388685 | 0.32607898 | -0.1127895 | HIP1R | Homo sapiens huntingtin interacting protein 1 related (HIP1R), transcript variant 1, mRNA. (from Re | NM_003959 |
| uc058uot.1 | 8.22E-04 | DOWN | 1.376099 | 1.376099 | 0.46058425 | 0.25862607 | -0.2019582 | HIP1R | Homo sapiens huntingtin interacting protein 1 related (HIP1R), transcript variant 1, mRNA. (from Re | NM_003959 |
| uc057zqa.1 | 7.13E-04 | DOWN | 1.2905959 | 1.2905959 | 0.36803734 | -0.5494239 | -0.9174612 | HPS5 | Homo sapiens Hermansky-Pudlak syndrome 5 (HPS5), transcript variant 3, mRNA. (from RefSeq NM | NM_181508 |
| uc063gye.1 | 6.47E-04 | DOWN | 1.553354 | 1.553354 | 0.63538665 | 1.3144798 | 0.6790931 | HSPA4 | Homo sapiens heat shock 70kDa protein 4 (HSPA4), mRNA. (from RefSeq NM_002154) | NM_002154 |
| uc057uwb.1 | 7.47E-04 | DOWN | 1.2488009 | 1.2488009 | 0.32054344 | 0.2864841 | -0.0340594 | IDE | Homo sapiens insulin-degrading enzyme (IDE), transcript variant 2, mRNA. (from RefSeq NM_0011€ | NM_001165946 |
| uc064sma.1 | 8.58E-04 | DOWN | 1.7223176 | 1.7223176 | 0.78435117 | -0.1169544 | -0.9013056 | IFT74 | Homo sapiens intraflagellar transport 74 (IFT74), transcript variant 4, mRNA. (from RefSeq NM_001 | NM_001099224 |
| uc058pgk.1 | 1.65E-04 | DOWN | 3.3863482 | 3.3863482 | 1.7597303 | -2.6947336 | -4.454464 | IKZF4 | Homo sapiens IKAROS family zinc finger 4 (Eos) (IKZF4), mRNA. (from RefSeq NM_022465) | NM_022465 |

| | | | | | | | | | | |
|---|---|---|---|---|---|---|---|---|---|---|
| uc060tla.1 | 6.58E-04 | DOWN | 1.2897521 | 1.2897521 | 0.36709383 | 0.50788546 | 0.14079161 | ILF3 | Homo sapiens interleukin enhancer binding factor 3, 90kDa (ILF3), transcript variant 4, mRNA. (from | NM_017620 |
| uc060urt.1 | 2.12E-04 | DOWN | 1.5014273 | 1.5014273 | 0.5863346 | -1.6479577 | -2.2342923 | ILVBL | Homo sapiens ilvB (bacterial acetolactate synthase)-like (ILVBL), mRNA. (from RefSeq NM_006844) | NM_006844 |
| uc057wxq.1 | 8.12E-04 | DOWN | 1.4821254 | 1.4821254 | 0.5676675 | 0.66837925 | 0.10071168 | INPP5A | Homo sapiens inositol polyphosphate-5-phosphatase, 40kDa (INPP5A), mRNA. (from RefSeq NM_00 | NM_005539 |
| uc065cai.1 | 9.36E-04 | DOWN | 1.3765228 | 1.3765228 | 0.4610285 | 0.7418396 | 0.28081113 | IRAK1 | Homo sapiens interleukin-1 receptor-associated kinase 1 (IRAK1), transcript variant 2, mRNA. (from | NM_001025242 |
| uc057sqo.1 | 8.11E-04 | DOWN | 1.2761558 | 1.2761558 | 0.3518045 | 0.99496365 | 0.64315915 | ITGB1 | Homo sapiens integrin, beta 1 (fibronectin receptor, beta polypeptide, antigen CD29 includes MDF2, | NM_033668 |
| uc061nqc.1 | 2.18E-04 | DOWN | 1.3883929 | 1.3883929 | 0.4734159 | 0.34611484 | -0.1273011 | IWS1 | Homo sapiens IWS1 homolog (S. cerevisiae) (IWS1), mRNA. (from RefSeq NM_017969) | NM_017969 |
| uc063hbu.1 | 3.33E-04 | DOWN | 1.3825978 | 1.3825978 | 0.46738154 | 1.1864761 | 0.7190946 | JADE2 | Homo sapiens jade family PHD finger 2 (JADE2), transcript variant 3, mRNA. (from RefSeq NM_0152 | NM_015288 |
| uc063hbv.1 | 9.85E-05 | DOWN | 1.6018566 | 1.6018566 | 0.67974496 | 0.4113808 | -0.2683642 | JADE2 | Homo sapiens jade family PHD finger 2 (JADE2), transcript variant 3, mRNA. (from RefSeq NM_0152 | NM_015288 |
| uc061lwd.1 | 4.05E-04 | DOWN | 1.5455755 | 1.5455755 | 0.62814415 | -0.2712648 | -0.8994089 | KANSL3 | Homo sapiens KAT8 regulatory NSL complex subunit 3 (KANSL3), transcript variant 7, non-coding RN | NR_047658 |
| uc058dom.1 | 7.30E-04 | DOWN | 1.2990743 | 1.2990743 | 0.37748393 | -0.4537181 | -0.831202 | KAT5 | Homo sapiens K(lysine) acetyltransferase 5 (KAT5), transcript variant 2, mRNA. (from RefSeq NM_0 | NM_006388 |
| uc060jxt.1 | 5.19E-04 | DOWN | 1.8570356 | 1.8570356 | 0.8930015 | -0.7108379 | -1.6038394 | KIAA0195 | Homo sapiens KIAA0195 (KIAA0195), mRNA. (from RefSeq NM_014738) | NM_014738 |
| uc059auf.1 | 5.86E-04 | DOWN | 1.333917 | 1.333917 | 0.4156689 | -0.4526337 | -0.8683025 | KIAA0391 | Homo sapiens KIAA0391 (KIAA0391), transcript variant 2, mRNA. (from RefSeq NM_001256678) | NM_001256678 |
| uc059bik.1 | 5.09E-04 | DOWN | 1.456567 | 1.456567 | 0.5425721 | -1.3735608 | -1.9161329 | L2HGDH | Homo sapiens L-2-hydroxyglutarate dehydrogenase (L2HGDH), mRNA. (from RefSeq NM_024884) | NM_024884 |
| uc011cfk.3 | 3.76E-04 | DOWN | 1.3542188 | 1.3542188 | 0.4374609 | 1.3142334 | 0.87677246 | LEF1 | Homo sapiens lymphoid enhancer-binding factor 1 (LEF1), transcript variant 4, mRNA. (from RefSeq | NM_001166119 |
| uc057otb.1 | 7.08E-04 | DOWN | 2.4232428 | 2.4232428 | 1.2769389 | -1.8144094 | -3.0913484 | LEMD1 | Homo sapiens LEM domain containing 1 (LEMD1), transcript variant 4, mRNA. (from RefSeq NM_00 | NM_001199052 |
| uc057otc.1 | 9.06E-04 | DOWN | 2.3645246 | 2.3645246 | 1.2415501 | -1.8387089 | -3.080259 | LEMD1 | Homo sapiens LEM domain containing 1 (LEMD1), transcript variant 4, mRNA. (from RefSeq NM_00 | NM_001199052 |
| uc058ujc.1 | 8.13E-04 | DOWN | 1.5301507 | 1.5301507 | 0.6136737 | 0.20963831 | -0.4040354 | LINC01089 | Homo sapiens long intergenic non-protein coding RNA 1089 (LINC01089), long non-coding RNA. (from | NR_002809 |
| uc057lwf.1 | 7.53E-04 | DOWN | 1.3924829 | 1.3924829 | 0.47765958 | -0.5281399 | -1.0057995 | LMNA | Homo sapiens lamin A/C (LMNA), transcript variant 3, mRNA. (from RefSeq NM_170708) | NM_170708 |
| uc058bpn.1 | 8.80E-04 | DOWN | 1.465787 | 1.465787 | 0.5516755 | 2.4074094 | 1.8557339 | LPXN | Homo sapiens leupaxin (LPXN), transcript variant 2, mRNA. (from RefSeq NM_004811) | NM_004811 |
| uc063ady.1 | 2.43E-04 | DOWN | 1.4906352 | 1.4906352 | 0.5759272 | 0.6129604 | 0.03703318 | LRBA | Homo sapiens LPS-responsive vesicle trafficking, beach and anchor containing (LRBA), transcript vari | NM_006726 |
| uc058kne.1 | 6.80E-04 | DOWN | 2.0665438 | 2.0665438 | 1.04722 | -1.6357126 | -2.6829326 | LRRC23 | Homo sapiens leucine rich repeat containing 23 (LRRC23), transcript variant 2, mRNA. (from RefSeq | NM_006992 |
| uc001akx.2 | 5.95E-04 | DOWN | 1.1888478 | 1.1888478 | 0.249564 | 2.672828 | 2.423264 | LRRC47 | Homo sapiens leucine rich repeat containing 47 (LRRC47), mRNA. (from RefSeq NM_020710) | NM_020710 |
| uc064cqe.1 | 7.30E-04 | DOWN | 1.5036224 | 1.5036224 | 0.5884423 | -0.5690464 | -1.1574887 | LSM5 | Homo sapiens LSM5 homolog, U6 small nuclear RNA associated (S. cerevisiae) (LSM5), transcript va | NM_012322 |
| uc057vlt.1 | 5.84E-04 | DOWN | 1.4735569 | 1.4735569 | 0.55930275 | -0.6038199 | -1.1631227 | LZTS2 | Homo sapiens leucine zipper, putative tumor suppressor 2 (LZTS2), mRNA. (from RefSeq NM_03242 | NM_032429 |
| uc059ian.1 | 4.97E-04 | DOWN | 1.4599111 | 1.4599111 | 0.5458805 | -0.4585696 | -1.0044502 | MAPKBP1 | Homo sapiens mitogen-activated protein kinase binding protein 1 (MAPKBP1), transcript variant 3, n | NM_001265611 |
| uc003fle.4 | 4.63E-04 | DOWN | 1.2460678 | 1.2460678 | 0.3173825 | -1.1091388 | -1.4265214 | MCCC1 | Homo sapiens methylcrotonoyl-CoA carboxylase 1 (alpha) (MCCC1), transcript variant 1, mRNA. (fro | NM_020166 |
| uc062qnd.1 | 5.07E-04 | DOWN | 1.2288607 | 1.2288607 | 0.2973214 | -1.1339347 | -1.4312562 | MCCC1 | Homo sapiens methylcrotonoyl-CoA carboxylase 1 (alpha) (MCCC1), transcript variant 1, mRNA. (fro | NM_020166 |
| uc062qne.1 | 3.54E-04 | DOWN | 1.240623 | 1.240623 | 0.31106478 | -1.1042471 | -1.4153118 | MCCC1 | Homo sapiens methylcrotonoyl-CoA carboxylase 1 (alpha) (MCCC1), transcript variant 1, mRNA. (fro | NM_020166 |
| uc003pnn.2 | 7.47E-04 | DOWN | 1.2560745 | 1.2560745 | 0.3289221 | 1.6296577 | 1.3007356 | MDN1 | Homo sapiens MDN1, midasin homolog (yeast) (MDN1), mRNA. (from RefSeq NM_014611) | NM_014611 |
| uc063qcd.1 | 9.47E-04 | DOWN | 1.3042611 | 1.3042611 | 0.38323268 | 1.5583802 | 1.1751475 | MDN1 | Homo sapiens MDN1, midasin homolog (yeast) (MDN1), mRNA. (from RefSeq NM_014611) | NM_014611 |
| uc062pue.1 | 1.81E-04 | DOWN | 4.079837 | 4.079837 | 2.0285115 | -4.594736 | -6.6232476 | MECOM | Homo sapiens MDS1 and EVI1 complex locus (MECOM), transcript variant 3, mRNA. (from RefSeq N | NM_001105078 |
| uc064ged.1 | 2.79E-04 | DOWN | 1.4110893 | 1.4110893 | 0.49680927 | 0.7099866 | 0.21317732 | MEPCE | Homo sapiens methylphosphate capping enzyme (MEPCE), transcript variant 4, mRNA. (from RefSe | NM_001194992 |
| uc059znk.1 | 6.16E-04 | DOWN | 1.3454988 | 1.3454988 | 0.4281411 | -0.032758 | -0.4608991 | METTL16 | Homo sapiens methyltransferase like 16 (METTL16), mRNA. (from RefSeq NM_024086) | NM_024086 |
| uc060ccx.1 | 8.12E-04 | DOWN | 1.4379041 | 1.4379041 | 0.52396744 | -1.4459757 | -1.9699432 | MIEF2 | Homo sapiens mitochondrial elongation factor 2 (MIEF2), transcript variant 1, mRNA. (from RefSeq | NM_139162 |
| uc064bkt.1 | 3.14E-04 | DOWN | 1.4664329 | 1.4664329 | 0.55231106 | 0.43986157 | -0.1124495 | MIOS | Homo sapiens missing oocyte, meiosis regulator, homolog (Drosophila) (MIOS), mRNA. (from RefSe | NM_019005 |
| uc058vgw.1 | 3.97E-05 | DOWN | 1.7731789 | 1.7731789 | 0.8263381 | -1.2798477 | -2.106186 | MMP17 | Homo sapiens matrix metallopeptidase 17 (membrane-inserted) (MMP17), mRNA. (from RefSeq NI | NM_016155 |
| uc057vfm.1 | 6.57E-04 | DOWN | 1.3661103 | 1.3661103 | 0.450074 | 0.26967993 | -0.180394 | MMS19 | Homo sapiens MMS19 nucleotide excision repair homolog (S. cerevisiae) (MMS19), transcript variar | NM_001289403 |
| uc059lni.1 | 5.86E-04 | DOWN | 1.6071565 | 1.6071565 | 0.6845104 | -0.0592606 | -0.7437711 | MPI | Homo sapiens mannose phosphate isomerase (MPI), transcript variant 2, mRNA. (from RefSeq NM_ | NM_001289155 |
| uc060fve.1 | 7.30E-04 | DOWN | 4.313879 | 4.313879 | 2.1089857 | -3.324458 | -5.4334435 | MPP2 | Homo sapiens membrane protein, palmitoylated 2 (MAGUK p55 subfamily member 2) (MPP2), tran | NM_001278381 |
| uc059mwq.1 | 3.05E-04 | DOWN | 1.7236552 | 1.7236552 | 0.7854712 | -1.5743408 | -2.359812 | MRPL46 | Homo sapiens mitochondrial ribosomal protein L46 (MRPL46), mRNA. (from RefSeq NM_022163) | NM_022163 |
| uc001yqy.4 | 4.37E-04 | DOWN | 1.2514856 | 1.2514856 | 0.32364166 | -0.9097973 | -1.233439 | MTA1 | Homo sapiens metastasis associated 1 (MTA1), transcript variant 2, mRNA. (from RefSeq NM_0012 | NM_001203258 |
| uc061ipp.1 | 9.01E-04 | DOWN | 2.817037 | 2.817037 | 1.4941785 | -2.2645373 | -3.7587159 | MTA3 | Homo sapiens metastasis associated 1 family, member 3 (MTA3), transcript variant 3, mRNA. (from | NM_020744 |
| uc057dns.1 | 9.79E-04 | DOWN | 1.446597 | 1.446597 | 0.53266305 | -0.863583 | -1.3962461 | MTFR1L | Homo sapiens mitochondrial fission regulator 1-like (MTFR1L), transcript variant 1, mRNA. (from Re | NM_019557 |
| uc063sgp.1 | 7.58E-04 | DOWN | 4.284698 | 4.284698 | 2.0991936 | -1.3402783 | -3.439472 | MTHFD1L | Homo sapiens methylenetetrahydrofolate dehydrogenase (NADP+ dependent) 1-like (MTHFD1L), tra | NM_001242768 |
| uc058xpe.1 | 8.43E-04 | DOWN | 1.3325596 | 1.3325596 | 0.41420004 | 4.2407594 | 3.8265593 | MYCBP2 | Homo sapiens MYC binding protein 2, E3 ubiquitin protein ligase (MYCBP2), mRNA. (from RefSeq NI | NM_015057 |
| uc001udy.2 | 4.55E-04 | DOWN | 1.3060995 | 1.3060995 | 0.38526484 | -2.370269 | -2.755534 | n/a | n/a | NM_001304835 |
| uc002gnz.5 | 2.10E-04 | DOWN | 1.3026936 | 1.3026936 | 0.3814978 | -0.1807804 | -0.5622781 | n/a | n/a | NM_018127 |
| uc002iii.2 | 1.67E-04 | DOWN | 1.4330951 | 1.4330951 | 0.51913434 | -0.6375163 | -1.1566507 | n/a | n/a | NM_001303442 |
| uc003zjz.4 | 5.30E-04 | DOWN | 1.2690427 | 1.2690427 | 0.34374064 | 0.7658224 | 0.4220818 | n/a | n/a | NR_046386 |
| uc010dzm.2 | 8.73E-04 | DOWN | 1.3376595 | 1.3376595 | 0.4197109 | -0.3204994 | -0.7402104 | n/a | n/a | NR_046366 |
| uc010yly.3 | 9.18E-04 | DOWN | 1.3024448 | 1.3024448 | 0.38122225 | 0.2236887 | -0.1575335 | n/a | n/a | NM_001271287 |
| uc032qsv.2 | 6.25E-04 | DOWN | 1.2440816 | 1.2440816 | 0.31508112 | -2.0031366 | -2.3182178 | n/a | n/a | NR_034118 |
| uc032qyp.2 | 4.80E-04 | DOWN | 1.248422 | 1.248422 | 0.3201057 | -1.9956144 | -2.31572 | n/a | n/a | NR_034118 |

| | | | | | | | | | | |
|---|---|---|---|---|---|---|---|---|---|---|
| uc032tgw.1 | 3.46E-04 | DOWN | 1.3213444 | 1.3213444 | 0.4020065 | -0.0667328 | -0.4687394 | n/a | n/a | NM_001278363 |
| uc057hsg.1 | 2.08E-04 | DOWN | 1.3992146 | 1.3992146 | 0.48461726 | -0.562811 | -1.0474283 | n/a | n/a | NR_073562 |
| uc057oir.1 | 6.77E-04 | DOWN | 1.4009339 | 1.4009339 | 0.48638883 | -0.2627614 | -0.7491502 | n/a | n/a | NM_001193570 |
| uc058tuf.1 | 2.47E-04 | DOWN | 1.5127581 | 1.5127581 | 0.5971813 | -0.8257096 | -1.422891 | n/a | n/a | NM_001206801 |
| uc058uon.1 | 9.24E-04 | DOWN | 1.3786023 | 1.3786023 | 0.4632063 | 0.87075204 | 0.4075457 | n/a | n/a | NM_001303097 |
| uc058upr.1 | 8.84E-04 | DOWN | 1.3141855 | 1.3141855 | 0.3941689 | -1.99234 | -2.386509 | n/a | n/a | NM_001304834 |
| uc059aun.1 | 6.11E-04 | DOWN | 1.5369658 | 1.5369658 | 0.6200851 | 0.47703028 | -0.1430549 | n/a | n/a | NR_104110 |
| uc059otm.1 | 6.89E-04 | DOWN | 2.245546 | 2.245546 | 1.1670663 | -0.8134839 | -1.9805502 | n/a | n/a | NR_033645 |
| uc060fvx.1 | 5.82E-04 | DOWN | 1.5452799 | 1.5452799 | 0.6278681 | -1.2433196 | -1.8711878 | n/a | n/a | NM_001304814 |
| uc060hxx.1 | 8.55E-04 | DOWN | 1.382876 | 1.382876 | 0.46767184 | 0.21683846 | -0.2508334 | n/a | n/a | NR_038416 |
| uc060myc.1 | 2.53E-04 | DOWN | 1.4927839 | 1.4927839 | 0.5780053 | -1.0690707 | -1.647076 | n/a | n/a | NM_001278684 |
| uc060nfo.1 | 7.90E-04 | DOWN | 1.5328991 | 1.5328991 | 0.6162628 | 1.4752246 | 0.8589618 | n/a | n/a | NM_001083625 |
| uc061irb.1 | 1.25E-05 | DOWN | 1.848503 | 1.848503 | 0.88635737 | -0.1049546 | -0.991312 | n/a | n/a | NR_073394 |
| uc061sne.1 | 9.73E-04 | DOWN | 2.2861962 | 2.2861962 | 1.1929492 | -1.8364338 | -3.029383 | n/a | n/a | NM_001079866 |
| uc061xwg.1 | 5.98E-04 | DOWN | 1.3093083 | 1.3093083 | 0.38880482 | -0.2577944 | -0.6465992 | n/a | n/a | NM_198433 |
| uc061xwh.1 | 2.95E-04 | DOWN | 1.3791691 | 1.3791691 | 0.46379936 | 0.08060125 | -0.3831982 | n/a | n/a | NM_198433 |
| uc061yty.1 | 3.04E-05 | DOWN | 1.5428617 | 1.5428617 | 0.62560874 | -1.9878901 | -2.613499 | n/a | n/a | NM_001283052 |
| uc062evq.1 | 2.26E-05 | DOWN | 1.2992173 | 1.2992173 | 0.37764278 | -0.3089729 | -0.6866157 | n/a | n/a | NR_104301 |
| uc062evt.1 | 1.31E-04 | DOWN | 1.3366652 | 1.3366652 | 0.4186381 | -0.4444166 | -0.8630547 | n/a | n/a | NR_104300 |
| uc062hvi.1 | 1.28E-04 | DOWN | 1.2769156 | 1.2769156 | 0.3526631 | 0.32500753 | -0.0276556 | n/a | n/a | NR_046352 |
| uc062jao.1 | 5.59E-04 | DOWN | 1.3062011 | 1.3062011 | 0.38537702 | -1.5060563 | -1.8914334 | n/a | n/a | NR_033815 |
| uc062jaq.1 | 8.53E-04 | DOWN | 1.245694 | 1.245694 | 0.31694975 | -0.8807747 | -1.1977245 | n/a | n/a | NR_033815 |
| uc062jar.1 | 9.22E-05 | DOWN | 1.3510718 | 1.3510718 | 0.43410438 | -1.4584565 | -1.892561 | n/a | n/a | NR_033815 |
| uc062lut.1 | 8.45E-04 | DOWN | 1.5484262 | 1.5484262 | 0.6308026 | -0.1032705 | -0.7340731 | n/a | n/a | NM_001261829 |
| uc062qnh.1 | 5.26E-04 | DOWN | 1.2573651 | 1.2573651 | 0.33040363 | -1.2823358 | -1.6127394 | n/a | n/a | NR_120639 |
| uc062xlo.1 | 8.12E-04 | DOWN | 1.3678064 | 1.3678064 | 0.45186406 | 1.3719277 | 0.9200637 | n/a | n/a | NR_103781 |
| uc062xlr.1 | 2.60E-04 | DOWN | 1.3787224 | 1.3787224 | 0.46333203 | 0.09416642 | -0.3691656 | n/a | n/a | NR_103782 |
| uc063exq.1 | 1.13E-04 | DOWN | 1.4738429 | 1.4738429 | 0.5595827 | 1.7643167 | 1.204734 | n/a | n/a | NM_001174071 |
| uc063ljh.1 | 1.93E-04 | DOWN | 1.6148275 | 1.6148275 | 0.6913801 | 0.365051 | -0.326329 | n/a | n/a | NM_001195286 |
| uc063msm.1 | 1.83E-04 | UP | 1.290776 | -1.290776 | -0.3682387 | -0.5414801 | -0.1732414 | n/a | n/a | NR_072994 |
| uc063twf.1 | 1.83E-04 | UP | 1.290776 | -1.290776 | -0.3682387 | -0.5414801 | -0.1732414 | n/a | n/a | NR_072994 |
| uc063vcd.1 | 1.83E-04 | UP | 1.290776 | -1.290776 | -0.3682387 | -0.5414801 | -0.1732414 | n/a | n/a | NR_072994 |
| uc063whs.1 | 1.83E-04 | UP | 1.290776 | -1.290776 | -0.3682387 | -0.5414801 | -0.1732414 | n/a | n/a | NR_072994 |
| uc063xhb.1 | 1.83E-04 | UP | 1.290776 | -1.290776 | -0.3682387 | -0.5414801 | -0.1732414 | n/a | n/a | NR_072994 |
| uc063ymx.1 | 2.12E-04 | UP | 1.296198 | -1.296198 | -0.3742861 | -0.6125109 | -0.2382247 | n/a | n/a | NR_072994 |
| uc063zss.1 | 1.83E-04 | UP | 1.290776 | -1.290776 | -0.3682387 | -0.5414801 | -0.1732414 | n/a | n/a | NR_072994 |
| uc064dvi.1 | 9.93E-04 | DOWN | 2.9565728 | 2.9565728 | 1.5639257 | -2.0152664 | -3.5791922 | n/a | n/a | NR_047689 |
| uc064efz.1 | 4.38E-04 | DOWN | 1.5643185 | 1.5643185 | 0.6455343 | -0.9788129 | -1.6243472 | n/a | n/a | NR_045512 |
| uc064pgy.1 | 4.83E-04 | DOWN | 2.1829476 | 2.1829476 | 1.1262776 | -0.5619285 | -1.6882058 | n/a | n/a | NR_126339 |
| uc064rdj.1 | 3.14E-04 | DOWN | 2.5641959 | 2.5641959 | 1.3585064 | -3.039708 | -4.3982143 | n/a | n/a | NR_110193 |
| uc064voi.1 | 9.36E-04 | DOWN | 1.5573264 | 1.5573264 | 0.6390714 | -0.0684742 | -0.7075455 | n/a | n/a | NR_033234 |
| uc059bwu.1 | 8.27E-04 | DOWN | 1.4354949 | 1.4354949 | 0.5215482 | 1.0612817 | 0.5397335 | NAA30 | Homo sapiens N(alpha)-acetyltransferase 30, NatC catalytic subunit (NAA30), mRNA. (from RefSeq | NM_001011713 |
| uc059qle.1 | 8.63E-04 | DOWN | 1.5101768 | 1.5101768 | 0.59471744 | -0.675169 | -1.2698864 | NAGPA | Homo sapiens N-acetylglucosamine-1-phosphodiester alpha-N-acetylglucosaminidase (NAGPA), mR | NM_016256 |
| uc064kqr.1 | 5.70E-04 | DOWN | 3.8234951 | 3.8234951 | 1.934892 | -1.4622906 | -3.3971827 | NAT1 | Homo sapiens N-acetyltransferase 1 (arylamine N-acetyltransferase) (NAT1), transcript variant 4, m | NM_001160173 |
| uc057jyz.1 | 1.78E-04 | DOWN | 1.4817319 | 1.4817319 | 0.5672844 | 0.01036535 | -0.556919 | NBPF15 | Homo sapiens neuroblastoma breakpoint family, member 15 (NBPF15), transcript variant 2, mRNA. | NM_173638 |
| uc057jza.1 | 7.35E-05 | DOWN | 1.5859307 | 1.5859307 | 0.66532975 | 0.4592986 | -0.2060311 | NBPF15 | Homo sapiens neuroblastoma breakpoint family, member 15 (NBPF15), transcript variant 2, mRNA. | NM_173638 |
| uc062jkq.1 | 5.92E-04 | DOWN | 1.3335292 | 1.3335292 | 0.41524944 | -0.0118366 | -0.427086 | NCKIPSD | Homo sapiens NCK interacting protein with SH3 domain (NCKIPSD), transcript variant 2, mRNA. (fror | NM_184231 |
| uc064ipg.1 | 8.92E-04 | DOWN | 1.445949 | 1.445949 | 0.53201663 | 0.47285008 | -0.0591665 | NDUFB2 | Homo sapiens NADH dehydrogenase (ubiquinone) 1 beta subcomplex, 2, 8kDa (NDUFB2), mRNA. (fr | NM_004546 |
| uc057mpx.1 | 3.84E-04 | DOWN | 1.3569298 | 1.3569298 | 0.44034606 | -0.0668979 | -0.5072439 | NDUFS2 | Homo sapiens NADH dehydrogenase (ubiquinone) Fe-S protein 2, 49kDa (NADH-coenzyme Q reducta | NM_004550 |
| uc062yok.1 | 4.26E-04 | DOWN | 1.2873061 | 1.2873061 | 0.36435512 | 1.2260548 | 0.86169964 | NFKB1 | Homo sapiens nuclear factor of kappa light polypeptide gene enhancer in B-cells 1 (NFKB1), transcri | NM_003998 |
| uc061end.1 | 5.97E-05 | DOWN | 2.8327382 | 2.8327382 | 1.5021973 | -4.5717025 | -6.0738997 | NLRP2, | Homo sapiens NLR family, pyrin domain containing 2 (NLRP2), transcript variant 2, mRNA. (from Re | NM_001174081 |
| uc061var.1 | 5.75E-05 | DOWN | 1.446402 | 1.446402 | 0.5324685 | 0.48531654 | -0.047152 | NOP56 | Homo sapiens NOP56 ribonucleoprotein (NOP56), transcript variant 1, mRNA. (from RefSeq NM_00 | NM_006392 |
| uc032awd.2 | 7.10E-04 | DOWN | 1.2948493 | 1.2948493 | 0.37278417 | 1.1093647 | 0.7365806 | NOP9 | Homo sapiens NOP9 nucleolar protein (NOP9), transcript variant 2, mRNA. (from RefSeq NM_00128 | NM_001286367 |

| | | | | | | | | | | |
|---|---|---|---|---|---|---|---|---|---|---|
| uc021txo.2 | 1.75E-06 | DOWN | 1.6627548 | 1.6627548 | 0.7335754 | -1.3857993 | -2.1193748 | NT5C3B | Homo sapiens 5'-nucleotidase, cytosolic IIIB (NT5C3B), transcript variant 1, mRNA. (from RefSeq NM | NM_052935 |
| uc060fdr.1 | 1.71E-04 | DOWN | 1.4781127 | 1.4781127 | 0.5637563 | -1.2733215 | -1.8370777 | NT5C3B | Homo sapiens 5'-nucleotidase, cytosolic IIIB (NT5C3B), transcript variant 1, mRNA. (from RefSeq NM | NM_052935 |
| uc061aza.1 | 2.60E-04 | DOWN | 1.7418319 | 1.7418319 | 0.8006054 | 0.66143537 | -0.13917 | NTF4 | Homo sapiens neurotrophin 4 (NTF4), mRNA. (from RefSeq NM_006179) | NM_006179 |
| uc058qqv.1 | 5.41E-04 | DOWN | 1.4575303 | 1.4575303 | 0.5435258 | -0.3868452 | -0.930371 | NUP107 | Homo sapiens nucleoporin 107kDa (NUP107), mRNA. (from RefSeq NM_020401) | NM_020401 |
| uc061bmx.1 | 1.57E-04 | DOWN | 1.4055872 | 1.4055872 | 0.49117294 | -0.087938 | -0.579111 | NUP62 | Homo sapiens nucleoporin 62kDa (NUP62), transcript variant 1, mRNA. (from RefSeq NM_153719) | NM_153719 |
| uc060jui.1 | 5.97E-04 | DOWN | 1.3628567 | 1.3628567 | 0.4466339 | -0.3735092 | -0.820143 | NUP85 | Homo sapiens nucleoporin 85kDa (NUP85), transcript variant 1, mRNA. (from RefSeq NM_024844) | NM_024844 |
| uc002gbo.3 | 3.72E-04 | DOWN | 1.237186 | 1.237186 | 0.30706236 | 0.6628111 | 0.3557488 | NUP88 | Homo sapiens nucleoporin 88kDa (NUP88), mRNA. (from RefSeq NM_002532) | NM_002532 |
| uc057qeh.1 | 2.25E-04 | DOWN | 1.4150653 | 1.4150653 | 0.5008686 | -0.540885 | -1.0417535 | OBSCN | Homo sapiens obscurin, cytoskeletal calmodulin and titin-interacting RhoGEF (OBSCN), transcript va | NM_001271223 |
| uc011mbf.3 | 7.49E-04 | DOWN | 1.2250093 | 1.2250093 | 0.29279274 | -0.8481388 | -1.1409315 | ODF2 | Homo sapiens outer dense fiber of sperm tails 2 (ODF2), transcript variant 7, mRNA. (from RefSeq N | NM_153440 |
| uc057fbq.1 | 8.30E-04 | DOWN | 1.3984331 | 1.3984331 | 0.48381123 | 0.2718339 | -0.2119774 | PABPC4 | Homo sapiens poly(A) binding protein, cytoplasmic 4 (inducible form) (PABPC4), transcript variant 3, | NM_001135654 |
| uc003aks.4 | 7.61E-04 | DOWN | 1.2578754 | 1.2578754 | 0.33098906 | 0.7356434 | 0.40465438 | PATZ1 | Homo sapiens POZ (BTB) and AT hook containing zinc finger 1 (PATZ1), transcript variant 4, mRNA. ( | NM_032051 |
| uc010mls.3 | 5.64E-04 | DOWN | 1.4451263 | 1.4451263 | 0.5311956 | -1.0197216 | -1.5509171 | PAX5 | Homo sapiens paired box 5 (PAX5), transcript variant 5, mRNA. (from RefSeq NM_001280550) | NM_001280550 |
| uc064kpk.1 | 8.79E-04 | DOWN | 1.4384214 | 1.4384214 | 0.52448636 | 1.3690842 | 0.8445979 | PCM1 | Homo sapiens pericentriolar material 1 (PCM1), mRNA. (from RefSeq NM_006197) | NM_006197 |
| uc062mbe.1 | 8.46E-04 | DOWN | 1.3801694 | 1.3801694 | 0.46484533 | 1.777595 | 1.3127497 | PCNP | Homo sapiens PEST proteolytic signal containing nuclear protein (PCNP), mRNA. (from RefSeq NM_( | NM_020357 |
| uc060wvu.1 | 9.69E-04 | DOWN | 1.4061683 | 1.4061683 | 0.4917693 | -0.0943106 | -0.5860799 | PDCD2L | Homo sapiens programmed cell death 2-like (PDCD2L), mRNA. (from RefSeq NM_032346) | NM_032346 |
| uc001emb.3 | 6.42E-04 | DOWN | 1.2513936 | 1.2513936 | 0.32353556 | 0.80455637 | 0.48102078 | PDE4DIP | Homo sapiens phosphodiesterase 4D interacting protein (PDE4DIP), transcript variant 5, mRNA. (fron | NM_001002811 |
| uc062alz.1 | 9.24E-04 | DOWN | 2.8799474 | 2.8799474 | 1.5260425 | -4.1637044 | -5.689747 | PDE9A | Homo sapiens phosphodiesterase 9A (PDE9A), transcript variant 2, mRNA. (from RefSeq NM_00100 | NM_001001567 |
| uc060baa.1 | 5.24E-04 | DOWN | 1.3054589 | 1.3054589 | 0.38455704 | -0.1416681 | -0.5262252 | PFAS | Homo sapiens phosphoribosylformylglycinamidine synthase (PFAS), mRNA. (from RefSeq NM_0123! | NM_012393 |
| uc057jvt.1 | 1.61E-04 | DOWN | 1.6271101 | 1.6271101 | 0.7023119 | -0.5270156 | -1.2293276 | PHGDH | Homo sapiens phosphoglycerate dehydrogenase (PHGDH), mRNA. (from RefSeq NM_006623) | NM_006623 |
| uc003try.3 | 4.05E-04 | DOWN | 1.296422 | 1.296422 | 0.3745354 | -0.0550644 | -0.4295999 | PHKG1 | Homo sapiens phosphorylase kinase, gamma 1 (muscle) (PHKG1), transcript variant 2, mRNA. (from | NM_006213 |
| uc021yzd.2 | 3.78E-04 | DOWN | 1.2906355 | 1.2906355 | 0.36808157 | 0.19874375 | -0.1693379 | PI16 | Homo sapiens peptidase inhibitor 16 (PI16), transcript variant 2, mRNA. (from RefSeq NM_0011991 | NM_001199159 |
| uc057xni.1 | 5.85E-04 | DOWN | 3.3347383 | 3.3347383 | 1.7375735 | -1.7080282 | -3.4456017 | PIDD1 | Homo sapiens p53-induced death domain protein 1 (PIDD1), transcript variant 3, mRNA. (from RefSe | NM_145887 |
| uc057xnk.1 | 8.87E-04 | DOWN | 1.6087366 | 1.6087366 | 0.68592817 | -0.6246703 | -1.3105985 | PIDD1 | Homo sapiens p53-induced death domain protein 1 (PIDD1), transcript variant 3, mRNA. (from RefSe | NM_145887 |
| uc059php.1 | 3.39E-04 | DOWN | 1.2870557 | 1.2870557 | 0.36407453 | -0.428887 | -0.7929616 | PKD1 | Homo sapiens polycystic kidney disease 1 (autosomal dominant) (PKD1), transcript variant 2, mRNA. | NM_000296 |
| uc059phr.1 | 3.64E-05 | DOWN | 1.7017137 | 1.7017137 | 0.76698834 | -0.359971 | -1.1269593 | PKD1 | Homo sapiens polycystic kidney disease 1 (autosomal dominant) (PKD1), transcript variant 2, mRNA. | NM_000296 |
| uc059rbn.1 | 4.55E-04 | DOWN | 1.6877521 | 1.6877521 | 0.75510305 | 2.3829587 | 1.6278557 | PLA2G10, | Homo sapiens phospholipase A2, group X (PLA2G10), mRNA. (from RefSeq NM_003561), | NM_003561 |
| uc009ypi.4 | 3.34E-04 | DOWN | 1.4851621 | 1.4851621 | 0.5706204 | 0.7913184 | 0.22069792 | PLCB3 | Homo sapiens phospholipase C, beta 3 (phosphatidylinositol-specific) (PLCB3), transcript variant 1, n | NM_000932 |
| uc062iet.1 | 2.54E-04 | DOWN | 1.521547 | 1.521547 | 0.60553885 | -0.1730668 | -0.7786057 | PLCD1 | Homo sapiens phospholipase C, delta 1 (PLCD1), transcript variant 2, mRNA. (from RefSeq NM_0062 | NM_006225 |
| uc061xai.1 | 1.07E-04 | DOWN | 1.7400681 | 1.7400681 | 0.79914373 | 0.25267655 | -0.5464672 | PLCG1 | Homo sapiens phospholipase C, gamma 1 (PLCG1), transcript variant 2, mRNA. (from RefSeq NM_1 | NM_182811 |
| uc059vsr.1 | 2.18E-04 | DOWN | 1.3588929 | 1.3588929 | 0.44243178 | -1.5788502 | -2.021282 | PLEKHG4 | Homo sapiens pleckstrin homology domain containing, family G (with RhoGef domain) member 4 (F | NM_001129729 |
| uc062erg.1 | 5.67E-04 | DOWN | 1.6051593 | 1.6051593 | 0.6827164 | -0.4386688 | -1.1213852 | PMM1 | Homo sapiens phosphomannomutase 1 (PMM1), mRNA. (from RefSeq NM_002676) | NM_002676 |
| uc064bfu.1 | 7.43E-04 | DOWN | 1.297185 | 1.297185 | 0.37538418 | 0.449991 | 0.07460678 | PMS2 | Homo sapiens PMS2 postmeiotic segregation increased 2 (S. cerevisiae) (PMS2), transcript variant 1 | NM_000535 |
| uc062avx.1 | 5.29E-04 | DOWN | 1.4505265 | 1.4505265 | 0.5365766 | -1.348824 | -1.8854007 | POFUT2 | Homo sapiens protein O-fucosyltransferase 2 (POFUT2), transcript variant 3, mRNA. (from RefSeq N | NM_133635 |
| uc062uom.1 | 3.79E-04 | DOWN | 2.7521698 | 2.7521698 | 1.4605695 | -1.5645882 | -3.0251577 | POLN | Homo sapiens polymerase (DNA directed) nu (POLN), mRNA. (from RefSeq NM_181808) | NM_181808 |
| uc057kca.1 | 7.84E-04 | DOWN | 1.3388258 | 1.3388258 | 0.42096826 | 1.2649206 | 0.84395236 | POLR3GL | Homo sapiens polymerase (RNA) III (DNA directed) polypeptide G (32kD)-like (POLR3GL), mRNA. (fr | NM_032305 |
| uc003baf.5 | 7.24E-04 | DOWN | 1.1986835 | 1.1986835 | 0.26145077 | 0.3619145 | 0.10046375 | POLR3H | Homo sapiens polymerase (RNA) III (DNA directed) polypeptide H (22.9kD) (POLR3H), transcript varia | NM_138338 |
| uc062eqw.1 | 3.06E-04 | DOWN | 1.3954544 | 1.3954544 | 0.48073497 | -0.283875 | -0.76461 | POLR3H | Homo sapiens polymerase (RNA) III (DNA directed) polypeptide H (22.9kD) (POLR3H), transcript varia | NM_001018050 |
| uc064emi.1 | 2.27E-04 | DOWN | 1.4989387 | 1.4989387 | 0.58394134 | 1.5621787 | 0.97823733 | POM121C | Homo sapiens POM121 transmembrane nucleoporin C (POM121C), mRNA. (from RefSeq NM_00109 | NM_001099415 |
| uc061isf.1 | 5.45E-04 | DOWN | 1.2608339 | 1.2608339 | 0.33437818 | 0.6451784 | 0.31080022 | PPM1B | Homo sapiens protein phosphatase, Mg2+/Mn2+ dependent, 1B (PPM1B), transcript variant 1, mRN | NM_002706 |
| uc058tfv.1 | 9.85E-04 | DOWN | 1.451177 | 1.451177 | 0.5372235 | 1.7413245 | 1.2041011 | PPP1CC | Homo sapiens protein phosphatase 1, catalytic subunit, gamma isozyme (PPP1CC), transcript variant | NM_002710 |
| uc058enj.1 | 9.40E-04 | DOWN | 1.4143714 | 1.4143714 | 0.500161 | 0.5976357 | 0.09747469 | PPP6R3 | Homo sapiens protein phosphatase 6, regulatory subunit 3 (PPP6R3), transcript variant 3, mRNA. (fr | NM_001164164 |
| uc058enm.1 | 9.88E-04 | DOWN | 1.4273974 | 1.4273974 | 0.513387 | 0.3181437 | -0.1952433 | PPP6R3 | Homo sapiens protein phosphatase 6, regulatory subunit 3 (PPP6R3), transcript variant 3, mRNA. (fr | NM_001164164 |
| uc064yij.1 | 9.54E-04 | DOWN | 1.3761176 | 1.3761176 | 0.46060374 | 0.3719382 | -0.0886656 | PRDX4 | Homo sapiens peroxiredoxin 4 (PRDX4), mRNA. (from RefSeq NM_006406) | NM_006406 |
| uc010smd.3 | 5.58E-04 | DOWN | 1.3640354 | 1.3640354 | 0.44788104 | 0.21469362 | -0.2331875 | PRKAG1 | Homo sapiens protein kinase, AMP-activated, gamma 1 non-catalytic subunit (PRKAG1), transcript v | NM_001206710 |
| uc010vlg.3 | 1.05E-04 | DOWN | 1.3053896 | 1.3053896 | 0.3844805 | -1.2878727 | -1.6723531 | PRMT7 | Homo sapiens protein arginine methyltransferase 7 (PRMT7), transcript variant 2, mRNA. (from Ref | NM_001184824 |
| uc059wfw.1 | 8.99E-04 | DOWN | 1.275288 | 1.275288 | 0.35082307 | -1.3989285 | -1.7497516 | PRMT7 | Homo sapiens protein arginine methyltransferase 7 (PRMT7), transcript variant 1, mRNA. (from Ref | NM_019023 |
| uc057knz.1 | 5.59E-04 | DOWN | 1.3038893 | 1.3038893 | 0.38282135 | 2.0640752 | 1.6812539 | PRPF3 | Homo sapiens pre-mRNA processing factor 3 (PRPF3), mRNA. (from RefSeq NM_004698) | NM_004698 |
| uc059zhu.1 | 9.42E-04 | DOWN | 1.4483182 | 1.4483182 | 0.53437865 | 1.2172068 | 0.68282825 | PRPF8, | Homo sapiens pre-mRNA processing factor 8 (PRPF8), mRNA. (from RefSeq NM_006445), | NM_006445 |
| uc064wqo.1 | 2.85E-04 | DOWN | 1.5161586 | 1.5161586 | 0.60042065 | 1.8911299 | 1.2907091 | PRRC2B | Homo sapiens proline-rich coiled-coil 2B (PRRC2B), mRNA. (from RefSeq NM_013318) | NM_013318 |
| uc060irr.1 | 4.01E-04 | DOWN | 1.2625949 | 1.2625949 | 0.33639187 | 0.5422088 | 0.20581692 | PSMC5 | Homo sapiens proteasome (prosome, macropain) 26S subunit, ATPase, 5 (PSMC5), transcript variant | NM_002805 |
| uc063mxz.1 | 7.17E-05 | DOWN | 4.6986156 | 4.6986156 | 2.2322357 | -1.4356651 | -3.6679008 | PSORS1C1, | Homo sapiens psoriasis susceptibility 1 candidate 1 (PSORS1C1), mRNA. (from RefSeq NM_014068) | NM_014068 |
| uc060qrc.1 | 2.06E-04 | DOWN | 1.292369 | 1.292369 | 0.37001806 | 0.46050623 | 0.09048816 | PTBP1 | Homo sapiens polypyrimidine tract binding protein 1 (PTBP1), transcript variant 2, mRNA. (from Ref | NM_031990 |
| uc060qrh.1 | 2.20E-04 | DOWN | 1.3203248 | 1.3203248 | 0.40089285 | 0.04292854 | -0.3579643 | PTBP1 | Homo sapiens polypyrimidine tract binding protein 1 (PTBP1), transcript variant 1, mRNA. (from Ref | NM_002819 |

| | | | | | | | | | | |
|---|---|---|---|---|---|---|---|---|---|---|
| uc064umt.1 | 8.19E-04 | DOWN | 2.0232575 | 2.0232575 | 1.0166799 | -2.7186441 | -3.7353241 | PTCH1 | Homo sapiens patched 1 (PTCH1), transcript variant 1d, mRNA. (from RefSeq NM_001083606) | NM_001083606 |
| uc064unb.1 | 3.59E-04 | DOWN | 3.1931295 | 3.1931295 | 1.6749711 | -3.068345 | -4.743316 | PTCH1 | Homo sapiens patched 1 (PTCH1), transcript variant 1c', mRNA. (from RefSeq NM_001083605) | NM_001083605 |
| uc057olm.1 | 4.21E-04 | DOWN | 1.3871766 | 1.3871766 | 0.4721515 | -0.8639333 | -1.3360848 | PTPN7 | Homo sapiens protein tyrosine phosphatase, non-receptor type 7 (PTPN7), transcript variant 1, mRN | NM_002832 |
| uc058mvt.1 | 7.78E-05 | DOWN | 1.4958481 | 1.4958481 | 0.5809636 | -0.5292943 | -1.110258 | PUS7L | Homo sapiens pseudouridylate synthase 7 homolog (S. cerevisiae)-like (PUS7L), transcript variant 2, | NM_001098614 |
| uc062aqk.1 | 9.50E-04 | DOWN | 1.5196857 | 1.5196857 | 0.603773 | 1.3084248 | 0.70465183 | PWP2 | Homo sapiens PWP2 periodic tryptophan protein homolog (yeast) (PWP2), mRNA. (from RefSeq NM | NM_005049 |
| uc058fzr.1 | 7.95E-04 | DOWN | 1.594856 | 1.594856 | 0.67342615 | -1.209675 | -1.8831011 | RAB30 | Homo sapiens RAB30, member RAS oncogene family (RAB30), transcript variant 2, mRNA. (from Re | NM_014488 |
| uc060uuc.1 | 3.13E-04 | DOWN | 1.4480727 | 1.4480727 | 0.534134 | 0.85487753 | 0.32074356 | RASAL3 | Homo sapiens RAS protein activator like 3 (RASAL3), mRNA. (from RefSeq NM_022904) | NM_022904 |
| uc004dhf.4 | 5.01E-04 | DOWN | 1.2375112 | 1.2375112 | 0.30744153 | 1.3686744 | 1.0612328 | RBM10 | Homo sapiens RNA binding motif protein 10 (RBM10), transcript variant 1, mRNA. (from RefSeq NM | NM_005676 |
| uc004dhi.4 | 5.18E-04 | DOWN | 1.2458704 | 1.2458704 | 0.31715393 | 1.4672886 | 1.1501347 | RBM10 | Homo sapiens RNA binding motif protein 10 (RBM10), transcript variant 5, mRNA. (from RefSeq NM | NM_001204468 |
| uc001tvn.5 | 3.05E-04 | DOWN | 1.2246418 | 1.2246418 | 0.29235983 | 0.4423038 | 0.149944 | RBM19 | Homo sapiens RNA binding motif protein 19 (RBM19), transcript variant 3, mRNA. (from RefSeq NM | NM_001146698 |
| uc057kbj.1 | 2.70E-04 | DOWN | 1.3268416 | 1.3268416 | 0.40799615 | 1.4242088 | 1.0162126 | RBM8A | Homo sapiens RNA binding motif protein 8A (RBM8A), mRNA. (from RefSeq NM_005105) | NM_005105 |
| uc058acp.1 | 6.77E-04 | DOWN | 1.4084407 | 1.4084407 | 0.4940988 | -0.7304082 | -1.224507 | RCN1 | Homo sapiens reticulocalbin 1, EF-hand calcium binding domain (RCN1), mRNA. (from RefSeq NM_( | NM_002901 |
| uc062vzw.1 | 7.82E-04 | DOWN | 1.3807817 | 1.3807817 | 0.4654852 | 0.57894766 | 0.11346246 | RFC1 | Homo sapiens replication factor C (activator 1) 1, 145kDa (RFC1), transcript variant 1, mRNA. (from | NM_002913 |
| uc059xcn.1 | 2.30E-04 | DOWN | 1.5203614 | 1.5203614 | 0.60441434 | -0.0619525 | -0.6663668 | RFWD3 | Homo sapiens ring finger and WD repeat domain 3 (RFWD3), mRNA. (from RefSeq NM_018124) | NM_018124 |
| uc032tgs.1 | 2.09E-04 | DOWN | 1.3263673 | 1.3263673 | 0.4074803 | -0.1065364 | -0.5140167 | RHOH | Homo sapiens ras homolog family member H (RHOH), transcript variant 4, mRNA. (from RefSeq NM | NM_001278362 |
| uc032tgt.1 | 2.67E-04 | DOWN | 1.315562 | 1.315562 | 0.39567924 | -0.0874111 | -0.4830904 | RHOH | Homo sapiens ras homolog family member H (RHOH), transcript variant 1, mRNA. (from RefSeq NM | NM_001278359 |
| uc032tgu.1 | 6.28E-04 | DOWN | 1.2880341 | 1.2880341 | 0.36517078 | -0.1437313 | -0.508902 | RHOH | Homo sapiens ras homolog family member H (RHOH), transcript variant 3, mRNA. (from RefSeq NM | NM_001278361 |
| uc032tgv.1 | 3.47E-04 | DOWN | 1.3125197 | 1.3125197 | 0.39233905 | -0.047764 | -0.440103 | RHOH | Homo sapiens ras homolog family member H (RHOH), transcript variant 2, mRNA. (from RefSeq NM | NM_001278360 |
| uc002jys.5 | 3.68E-04 | DOWN | 1.3271444 | 1.3271444 | 0.4083253 | 0.15025906 | -0.2580662 | RPTOR | Homo sapiens regulatory associated protein of MTOR, complex 1 (RPTOR), transcript variant 2, mRN | NM_001163034 |
| uc010wuf.2 | 9.99E-04 | DOWN | 1.3079842 | 1.3079842 | 0.38734514 | 0.27418116 | -0.113164 | RPTOR | Homo sapiens regulatory associated protein of MTOR, complex 1 (RPTOR), transcript variant 2, mRN | NM_001163034 |
| uc003znj.4 | 4.50E-04 | DOWN | 1.2600447 | 1.2600447 | 0.3334749 | 2.897482 | 2.564007 | RRAGA | Homo sapiens Ras-related GTP binding A (RRAGA), mRNA. (from RefSeq NM_006570) | NM_006570 |
| uc001mlf.5 | 4.41E-04 | DOWN | 1.411432 | 1.411432 | 0.49715966 | 0.8156739 | 0.3185142 | RRAS2 | Homo sapiens related RAS viral (r-ras) oncogene homolog 2 (RRAS2), transcript variant 1, mRNA. (f | NM_012250 |
| uc060hiq.1 | 7.17E-04 | DOWN | 1.3859949 | 1.3859949 | 0.47092196 | 0.01118546 | -0.4597365 | RSAD1 | Homo sapiens radical S-adenosyl methionine domain containing 1 (RSAD1), transcript variant 1, mR | NM_018346 |
| uc062oaq.1 | 1.48E-04 | DOWN | 1.4572004 | 1.4572004 | 0.5431993 | 0.11373038 | -0.4294689 | RYK | Homo sapiens receptor-like tyrosine kinase (RYK), transcript variant 2, mRNA. (from RefSeq NM_00 | NM_002958 |
| uc061akp.1 | 9.04E-04 | DOWN | 1.2700317 | 1.2700317 | 0.3448645 | 0.53511864 | 0.19025409 | SAE1 | Homo sapiens SUMO1 activating enzyme subunit 1 (SAE1), transcript variant 1, mRNA. (from RefSe | NM_005500 |
| uc010swx.2 | 3.49E-04 | DOWN | 1.2962941 | 1.2962941 | 0.37439305 | 0.9367518 | 0.5623587 | SART3 | Homo sapiens squamous cell carcinoma antigen recognized by T cells 3 (SART3), mRNA. (from RefS | NM_014706 |
| uc058suo.1 | 4.11E-04 | DOWN | 1.2769626 | 1.2769626 | 0.3527163 | 0.61727655 | 0.2645602 | SART3 | Homo sapiens squamous cell carcinoma antigen recognized by T cells 3 (SART3), mRNA. (from RefS | NM_014706 |
| uc062xkn.1 | 9.64E-04 | DOWN | 1.4737791 | 1.4737791 | 0.5595203 | 1.5154352 | 0.9559149 | SDAD1 | Homo sapiens SDA1 domain containing 1 (SDAD1), transcript variant 1, mRNA. (from RefSeq NM_0 | NM_018115 |
| uc059ahe.1 | 8.49E-04 | DOWN | 1.2069174 | 1.2069174 | 0.27132696 | -0.8409004 | -1.1122273 | SDR39U1 | Homo sapiens short chain dehydrogenase/reductase family 39U, member 1 (SDR39U1), transcript v | NM_020195 |
| uc062bvk.1 | 1.94E-04 | DOWN | 1.4599779 | 1.4599779 | 0.5459465 | -0.1899279 | -0.7358744 | SERPIND1 | Homo sapiens serpin peptidase inhibitor, clade D (heparin cofactor), member 1 (SERPIND1), mRNA. | NM_000185 |
| uc058fon.1 | 9.08E-04 | DOWN | 1.6135396 | 1.6135396 | 0.69022894 | -0.5334949 | -1.2237239 | SERPINH1 | Homo sapiens serpin peptidase inhibitor, clade H (heat shock protein 47), member 1, (collagen bindi | NM_001207014 |
| uc058czs.1 | 7.69E-04 | DOWN | 1.4519949 | 1.4519949 | 0.53803635 | 0.74744505 | 0.20940872 | SF1 | Homo sapiens splicing factor 1 (SF1), transcript variant 5, mRNA. (from RefSeq NM_001178031) | NM_001178031 |
| uc003alf.5 | 2.22E-04 | DOWN | 1.2294629 | 1.2294629 | 0.29802814 | -0.3474678 | -0.645496 | SFI1 | Homo sapiens Sfi1 homolog, spindle assembly associated (yeast) (SFI1), transcript variant 2, mRNA. | NM_014775 |
| uc062djn.1 | 2.90E-04 | DOWN | 1.238267 | 1.238267 | 0.30832237 | -0.373285 | -0.6816074 | SFI1 | Homo sapiens Sfi1 homolog, spindle assembly associated (yeast) (SFI1), transcript variant 1, mRNA. | NM_001007467 |
| uc062djs.1 | 8.84E-05 | DOWN | 1.338568 | 1.338568 | 0.4206904 | -0.2944833 | -0.7151737 | SFI1 | Homo sapiens Sfi1 homolog, spindle assembly associated (yeast) (SFI1), transcript variant 2, mRNA. | NM_014775 |
| uc061kon.1 | 2.51E-04 | DOWN | 1.350485 | 1.350485 | 0.43347758 | -1.6922766 | -2.125754 | SFXN5 | Homo sapiens sideroflexin 5 (SFXN5), mRNA. (from RefSeq NM_144579) | NM_144579 |
| uc058hsd.1 | 9.84E-04 | DOWN | 1.4435725 | 1.4435725 | 0.5296436 | -0.0018167 | -0.5314603 | SIDT2 | Homo sapiens SID1 transmembrane family, member 2 (SIDT2), mRNA. (from RefSeq NM_00104045 | NM_001040455 |
| uc059wbp.1 | 9.20E-04 | DOWN | 1.3850757 | 1.3850757 | 0.4699648 | -0.9814606 | -1.4514254 | SLC12A4 | Homo sapiens solute carrier family 12 (potassium/chloride transporter), member 4 (SLC12A4), trans | NM_001145964 |
| uc064wao.1 | 8.04E-05 | DOWN | 1.7162374 | 1.7162374 | 0.77924913 | -0.233956 | -1.0132052 | SLC25A25 | Homo sapiens solute carrier family 25 (mitochondrial carrier; phosphate carrier), member 25 (SLC25 | NM_052901 |
| uc002gku.1 | 6.72E-04 | DOWN | 1.2737942 | 1.2737942 | 0.34913218 | -0.1626447 | -0.5117768 | SLC25A35 | Homo sapiens solute carrier family 25, member 35 (SLC25A35), mRNA. (from RefSeq NM_201520) | NM_201520 |
| uc003etr.3 | 9.13E-04 | DOWN | 1.2488834 | 1.2488834 | 0.32063875 | -0.4139556 | -0.7345943 | SLC25A36 | Homo sapiens solute carrier family 25 (pyrimidine nucleotide carrier), member 36 (SLC25A36), trans | NM_001104647 |
| uc062okh.1 | 5.48E-04 | DOWN | 1.5024016 | 1.5024016 | 0.5872705 | -0.638229 | -1.2254995 | SLC25A36 | Homo sapiens solute carrier family 25 (pyrimidine nucleotide carrier), member 36 (SLC25A36), trans | NM_018155 |
| uc062kbt.1 | 4.06E-04 | DOWN | 3.7354627 | 3.7354627 | 1.901287 | -1.2004913 | -3.1017783 | SLC38A3 | Homo sapiens solute carrier family 38, member 3 (SLC38A3), mRNA. (from RefSeq NM_006841) | NM_006841 |
| uc002gur.3 | 2.37E-04 | DOWN | 1.2505794 | 1.2505794 | 0.3225966 | 0.63603765 | 0.313441 | SLC5A10 | Homo sapiens solute carrier family 5 (sodium/sugar cotransporter), member 10 (SLC5A10), transcri | NM_001282417 |
| uc033eer.2 | 6.09E-04 | DOWN | 1.4189857 | 1.4189857 | 0.50486004 | 0.7264187 | 0.22155859 | SMC1A | Homo sapiens structural maintenance of chromosomes 1A (SMC1A), transcript variant 1, mRNA. (fr | NM_006306 |
| uc061ver.1 | 1.67E-04 | DOWN | 1.4434175 | 1.4434175 | 0.5294887 | 1.5320424 | 1.0025537 | SMOX | Homo sapiens spermine oxidase (SMOX), transcript variant 2, mRNA. (from RefSeq NM_175840) | NM_175840 |
| uc001sff.3 | 2.94E-04 | DOWN | 1.298578 | 1.298578 | 0.37693268 | -1.2101015 | -1.5870342 | SMUG1 | Homo sapiens single-strand-selective monofunctional uracil-DNA glycosylase 1 (SMUG1), transcript | NM_014311 |
| uc058ouu.1 | 8.45E-04 | DOWN | 1.2666962 | 1.2666962 | 0.34107056 | -1.1676779 | -1.5087484 | SMUG1 | Homo sapiens single-strand-selective monofunctional uracil-DNA glycosylase 1 (SMUG1), transcript | NM_014311 |
| uc064hsz.1 | 5.23E-04 | DOWN | 1.3600539 | 1.3600539 | 0.4436638 | 1.671265 | 1.2276012 | SND1 | Homo sapiens staphylococcal nuclease and tudor domain containing 1 (SND1), mRNA. (from RefSeq | NM_014390 |
| uc002dby.6 | 3.75E-04 | DOWN | 1.2043747 | 1.2043747 | 0.26828426 | 2.2939608 | 2.0256765 | SNX29 | Homo sapiens sorting nexin 29 (SNX29), mRNA. (from RefSeq NM_032167) | NM_032167 |
| uc064pbh.1 | 8.48E-04 | DOWN | 1.5363804 | 1.5363804 | 0.61953545 | -0.249683 | -0.8692184 | SNX31 | Homo sapiens sorting nexin 31 (SNX31), mRNA. (from RefSeq NM_152628) | NM_152628 |
| uc064kzy.1 | 9.63E-04 | DOWN | 1.5874264 | 1.5874264 | 0.6666897 | -0.9390299 | -1.6057196 | SORBS3 | Homo sapiens sorbin and SH3 domain containing 3 (SORBS3), transcript variant 1, mRNA. (from Ref | NM_005775 |
| uc064szm.1 | 7.92E-04 | DOWN | 1.5565406 | 1.5565406 | 0.6383432 | -1.0379893 | -1.6763325 | SPAG8 | Homo sapiens sperm associated antigen 8 (SPAG8), transcript variant 1, mRNA. (from RefSeq NM_( | NM_001039592 |

| | | | | | | | | | | |
|---|---|---|---|---|---|---|---|---|---|---|
| uc064tit.1 | 1.96E-04 | DOWN | 4.9173074 | 4.9173074 | 2.2978685 | -2.7281444 | -5.026013 | SPATA31A7 | Homo sapiens SPATA31 subfamily A, member 7 (SPATA31A7), mRNA. (from RefSeq NM_015667) | NM_015667 |
| uc001qrl.4 | 5.75E-04 | DOWN | 1.3101797 | 1.3101797 | 0.3897647 | 0.44171762 | 0.05195296 | SPSB2 | Homo sapiens splA/ryanodine receptor domain and SOCS box containing 2 (SPSB2), transcript variar | NM_001146316 |
| uc001qrm.4 | 3.64E-04 | DOWN | 1.3169955 | 1.3169955 | 0.3972504 | 0.4247893 | 0.0275389 | SPSB2 | Homo sapiens splA/ryanodine receptor domain and SOCS box containing 2 (SPSB2), transcript variar | NM_032641 |
| uc063eby.1 | 3.09E-04 | DOWN | 1.4520903 | 1.4520903 | 0.5381311 | 0.7738885 | 0.23575735 | SREK1 | Homo sapiens splicing regulatory glutamine/lysine-rich protein 1 (SREK1), transcript variant 4, mRN | NM_001270493 |
| uc059cvw.1 | 4.53E-04 | DOWN | 1.6296676 | 1.6296676 | 0.70457774 | 1.7157656 | 1.0111878 | SRSF5 | Homo sapiens serine/arginine-rich splicing factor 5 (SRSF5), transcript variant 2, mRNA. (from RefS | NM_006925 |
| uc002kvn.4 | 8.22E-04 | DOWN | 1.3419981 | 1.3419981 | 0.42438263 | 0.2535786 | -0.1708041 | SS18 | Homo sapiens synovial sarcoma translocation, chromosome 18 (SS18), transcript variant 2, mRNA. ( | NM_005637 |
| uc058mcg.1 | 5.83E-04 | DOWN | 2.3022642 | 2.3022642 | 1.2030534 | -0.5542331 | -1.7572865 | SSPN | Homo sapiens sarcospan (SSPN), transcript variant 2, mRNA. (from RefSeq NM_001135823) | NM_001135823 |
| uc064qlp.1 | 7.84E-04 | DOWN | 1.3147906 | 1.3147906 | 0.39483306 | 1.9079814 | 1.5131483 | ST3GAL1 | Homo sapiens ST3 beta-galactoside alpha-2,3-sialyltransferase 1 (ST3GAL1), transcript variant 2, m | NM_173344 |
| uc010rnb.2 | 2.05E-04 | DOWN | 1.3715278 | 1.3715278 | 0.45578384 | 1.8385347 | 1.3827509 | STIP1 | Homo sapiens stress-induced phosphoprotein 1 (STIP1), transcript variant 3, mRNA. (from RefSeq N | NM_001282653 |
| uc003nyt.4 | 5.77E-04 | DOWN | 2.892846 | 2.892846 | 1.5324895 | -3.682356 | -5.2148457 | STK19 | Homo sapiens serine/threonine kinase 19 (STK19), transcript variant 3, non-coding RNA. (from RefS | NR_026717 |
| uc061uro.1 | 4.79E-04 | DOWN | 1.4358821 | 1.4358821 | 0.52193725 | -0.4958884 | -1.0178257 | STK25 | Homo sapiens serine/threonine kinase 25 (STK25), transcript variant 4, mRNA. (from RefSeq NM_0( | NM_006374 |
| uc057jcy.1 | 1.87E-04 | DOWN | 1.4310664 | 1.4310664 | 0.5170906 | 0.70703584 | 0.18994522 | STRIP1 | Homo sapiens striatin interacting protein 1 (STRIP1), transcript variant 2, mRNA. (from RefSeq NM_ | NM_001270768 |
| uc060vzi.1 | 4.07E-04 | DOWN | 1.3941176 | 1.3941176 | 0.47935227 | -0.6710272 | -1.1503794 | SUGP1 | Homo sapiens SURP and G patch domain containing 1 (SUGP1), mRNA. (from RefSeq NM_172231) | NM_172231 |
| uc060vzm.1 | 3.18E-04 | DOWN | 1.4322947 | 1.4322947 | 0.51832837 | -0.7515937 | -1.269922 | SUGP1 | Homo sapiens SURP and G patch domain containing 1 (SUGP1), mRNA. (from RefSeq NM_172231) | NM_172231 |
| uc057yyi.1 | 3.18E-04 | DOWN | 1.4614923 | 1.4614923 | 0.5474422 | 0.6499431 | 0.10250086 | SWAP70 | Homo sapiens SWAP switching B-cell complex 70kDa subunit (SWAP70), transcript variant 1, mRNA | NM_015055 |
| uc059cwr.1 | 2.10E-04 | DOWN | 1.4609756 | 1.4609756 | 0.5469321 | 0.7396915 | 0.19275932 | SYNJ2BP | Homo sapiens synaptojanin 2 binding protein (SYNJ2BP), mRNA. (from RefSeq NM_018373) | NM_018373 |
| uc002kvt.5 | 3.64E-04 | DOWN | 1.2995728 | 1.2995728 | 0.37803748 | -0.21532 | -0.5933575 | TAF4B | Homo sapiens TAF4b RNA polymerase II, TATA box binding protein (TBP)-associated factor, 105kDa | NM_001293725 |
| uc062vjo.1 | 5.50E-04 | DOWN | 1.3021791 | 1.3021791 | 0.3809279 | 0.78185314 | 0.40092528 | TAPT1 | Homo sapiens transmembrane anterior posterior transformation 1 (TAPT1), mRNA. (from RefSeq N | NM_153365 |
| uc062dbn.1 | 5.92E-04 | DOWN | 1.47967 | 1.47967 | 0.5652755 | 0.32357368 | -0.2417019 | TBC1D10A | Homo sapiens TBC1 domain family, member 10A (TBC1D10A), transcript variant 2, mRNA. (from Re | NM_031937 |
| uc059pdz.1 | 6.61E-04 | DOWN | 1.3326174 | 1.3326174 | 0.41426262 | -0.5980837 | -1.0123463 | TBL3 | Homo sapiens transducin (beta)-like 3 (TBL3), mRNA. (from RefSeq NM_006453) | NM_006453 |
| uc060rek.1 | 1.03E-04 | DOWN | 1.3305947 | 1.3305947 | 0.41207114 | 0.13917707 | -0.2728941 | TCF3 | Homo sapiens transcription factor 3 (TCF3), transcript variant 1, mRNA. (from RefSeq NM_003200) | NM_003200 |
| uc061znt.1 | 7.03E-04 | DOWN | 1.4835258 | 1.4835258 | 0.56903 | -1.0095584 | -1.5785884 | TCP10L | Homo sapiens t-complex 10-like (TCP10L), mRNA. (from RefSeq NM_144659) | NM_144659 |
| uc009wnb.2 | 4.04E-04 | DOWN | 1.2925551 | 1.2925551 | 0.37022576 | -1.1198666 | -1.4900924 | TDRKH | Homo sapiens tudor and KH domain containing (TDRKH), transcript variant 3, mRNA. (from RefSeq N | NM_006862 |
| uc057kyy.1 | 1.87E-04 | DOWN | 1.2944707 | 1.2944707 | 0.37236226 | -1.8051876 | -2.1775498 | TDRKH | Homo sapiens tudor and KH domain containing (TDRKH), transcript variant 2, mRNA. (from RefSeq N | NM_001083963 |
| uc060upn.1 | 1.44E-04 | DOWN | 1.7235485 | 1.7235485 | 0.7853819 | -0.249048 | -1.0344299 | TECR | Homo sapiens trans-2,3-enoyl-CoA reductase (TECR), transcript variant 1, mRNA. (from RefSeq NM_ | NM_138501 |
| uc057lnm.1 | 7.68E-04 | DOWN | 2.6156912 | 2.6156912 | 1.3871922 | -0.9821219 | -2.3693142 | THBS3 | Homo sapiens thrombospondin 3 (THBS3), transcript variant 3, mRNA. (from RefSeq NM_00125260 | NM_001252608 |
| uc061kij.1 | 1.82E-04 | DOWN | 1.5086564 | 1.5086564 | 0.5932642 | 0.92305785 | 0.3297936 | TIA1 | Homo sapiens TIA1 cytotoxic granule-associated RNA binding protein (TIA1), transcript variant 2, mR | NM_022173 |
| uc060vxm.1 | 9.17E-06 | DOWN | 6.3920336 | 6.3920336 | 2.676275 | -0.8566036 | -3.5328786 | TMEM161A | Homo sapiens transmembrane protein 161A (TMEM161A), transcript variant 2, mRNA. (from RefSe | NM_001256766 |
| uc002cze.3 | 7.29E-04 | DOWN | 1.3421794 | 1.3421794 | 0.42457753 | 1.0684067 | 0.64382917 | TMEM186 | Homo sapiens transmembrane protein 186 (TMEM186), mRNA. (from RefSeq NM_015421) | NM_015421 |
| uc058qzc.1 | 4.84E-04 | DOWN | 1.4507658 | 1.4507658 | 0.5368147 | 0.22629212 | -0.3105226 | TMEM19 | Homo sapiens transmembrane protein 19 (TMEM19), mRNA. (from RefSeq NM_018279) | NM_018279 |
| uc062bjc.1 | 9.17E-04 | DOWN | 3.3256276 | 3.3256276 | 1.7336266 | -2.2503574 | -3.983984 | TMEM191B | Homo sapiens transmembrane protein 191B (TMEM191B), mRNA. (from RefSeq NM_001242313) | NM_001242313 |
| uc058ssh.1 | 3.91E-06 | DOWN | 1.5409752 | 1.5409752 | 0.62384367 | 0.46464792 | -0.1591958 | TMEM263 | Homo sapiens transmembrane protein 263 (TMEM263), mRNA. (from RefSeq NM_152261) | NM_152261 |
| uc057pch.1 | 8.78E-04 | DOWN | 1.4623923 | 1.4623923 | 0.5483304 | 1.9409966 | 1.3926662 | TRAF3IP3 | Homo sapiens TRAF3 interacting protein 3 (TRAF3IP3), transcript variant 2, mRNA. (from RefSeq NM | NM_001287754 |
| uc060dem.1 | 9.16E-04 | DOWN | 1.3961633 | 1.3961633 | 0.48146772 | -0.4331847 | -0.9146524 | TRAF4 | Homo sapiens TNF receptor-associated factor 4 (TRAF4), mRNA. (from RefSeq NM_004295) | NM_004295 |
| uc011ekt.3 | 6.76E-04 | DOWN | 1.8590623 | 1.8590623 | 0.8945751 | -5.1601963 | -6.0547714 | TRIM26, | Homo sapiens tripartite motif containing 26 (TRIM26), transcript variant 1, mRNA. (from RefSeq NM | NM_003449 |
| uc011exk.3 | 7.58E-04 | DOWN | 1.5816913 | 1.5816913 | 0.661468 | -4.2868767 | -4.9483447 | TRIM26, | Homo sapiens tripartite motif containing 26 (TRIM26), transcript variant 1, mRNA. (from RefSeq NM | NM_003449 |
| uc011fvx.3 | 6.76E-04 | DOWN | 1.8590623 | 1.8590623 | 0.8945751 | -5.1601963 | -6.0547714 | TRIM26, | Homo sapiens tripartite motif containing 26 (TRIM26), transcript variant 1, mRNA. (from RefSeq NM | NM_003449 |
| uc011ihz.3 | 6.76E-04 | DOWN | 1.8590623 | 1.8590623 | 0.8945751 | -5.1601963 | -6.0547714 | TRIM26, | Homo sapiens tripartite motif containing 26 (TRIM26), transcript variant 1, mRNA. (from RefSeq NM | NM_003449 |
| uc061dud.1 | 5.87E-04 | DOWN | 1.3993151 | 1.3993151 | 0.4847209 | 1.599368 | 1.114647 | TRIM28 | Homo sapiens tripartite motif containing 28 (TRIM28), mRNA. (from RefSeq NM_005762) | NM_005762 |
| uc061duf.1 | 1.33E-04 | DOWN | 1.4319136 | 1.4319136 | 0.51794446 | 1.4607326 | 0.9427881 | TRIM28 | Homo sapiens tripartite motif containing 28 (TRIM28), mRNA. (from RefSeq NM_005762) | NM_005762 |
| uc059pfx.1 | 4.33E-04 | DOWN | 1.3908731 | 1.3908731 | 0.47599077 | -0.7714217 | -1.2474126 | TSC2 | Homo sapiens tuberous sclerosis 2 (TSC2), transcript variant 4, mRNA. (from RefSeq NM_00107718 | NM_001077183 |
| uc058qdh.1 | 3.68E-04 | DOWN | 1.457643 | 1.457643 | 0.54363745 | -0.9739726 | -1.5176101 | TSFM | Homo sapiens Ts translation elongation factor, mitochondrial (TSFM), transcript variant 2, mRNA. (f | NM_005726 |
| uc064ebm.1 | 2.61E-04 | DOWN | 1.3691713 | 1.3691713 | 0.45330292 | -0.3239483 | -0.7772512 | TYW1 | Homo sapiens tRNA-yW synthesizing protein 1 homolog (S. cerevisiae) (TYW1), mRNA. (from RefSe | NM_018264 |
| uc010gpe.4 | 6.41E-04 | DOWN | 1.2603626 | 1.2603626 | 0.33383888 | 0.6372262 | 0.3033874 | UBASH3A | Homo sapiens ubiquitin associated and SH3 domain containing A (UBASH3A), transcript variant 3, m | NM_001243467 |
| uc062atf.1 | 1.78E-04 | DOWN | 1.3657991 | 1.3657991 | 0.44974524 | 0.73383886 | 0.28409365 | UBE2G2 | Homo sapiens ubiquitin-conjugating enzyme E2G 2 (UBE2G2), transcript variant 1, mRNA. (from Re | NM_003343 |
| uc060kwg.1 | 2.83E-04 | DOWN | 1.2708144 | 1.2708144 | 0.34575337 | -0.2679234 | -0.6136767 | USP36 | Homo sapiens ubiquitin specific peptidase 36 (USP36), mRNA. (from RefSeq NM_025090) | NM_025090 |
| uc061lir.1 | 8.90E-04 | DOWN | 1.2361414 | 1.2361414 | 0.30584383 | 0.01851674 | -0.287327 | USP39 | Homo sapiens ubiquitin specific peptidase 39 (USP39), transcript variant 2, mRNA. (from RefSeq NM | NM_001256725 |
| uc001xss.4 | 4.40E-04 | DOWN | 1.3755202 | 1.3755202 | 0.45997736 | 0.10258385 | -0.3573935 | VASH1 | Homo sapiens vasohibin 1 (VASH1), mRNA. (from RefSeq NM_014909) | NM_014909 |
| uc001ucd.4 | 8.90E-04 | DOWN | 1.2559216 | 1.2559216 | 0.3287464 | 2.1484215 | 1.8196751 | VPS33A | Homo sapiens vacuolar protein sorting 33 homolog A (S. cerevisiae) (VPS33A), mRNA. (from RefSeq | NM_022916 |
| uc058deo.1 | 8.74E-04 | DOWN | 1.3332107 | 1.3332107 | 0.4149048 | 1.3796762 | 0.96477145 | VPS51 | Homo sapiens vacuolar protein sorting 51 homolog (S. cerevisiae) (VPS51), transcript variant 1, mRN | NM_013265 |
| uc058dep.1 | 1.26E-04 | DOWN | 1.4589694 | 1.4589694 | 0.5449496 | 1.801518 | 1.2565684 | VPS51 | Homo sapiens vacuolar protein sorting 51 homolog (S. cerevisiae) (VPS51), transcript variant 1, mRN | NM_013265 |
| uc058des.1 | 3.86E-04 | DOWN | 1.4673897 | 1.4673897 | 0.55325204 | 2.1826818 | 1.6294297 | VPS51 | Homo sapiens vacuolar protein sorting 51 homolog (S. cerevisiae) (VPS51), transcript variant 1, mRN | NM_013265 |
| uc057ixb.1 | 3.09E-04 | DOWN | 1.4779993 | 1.4779993 | 0.5636456 | 0.5589262 | -0.0047194 | WDR47 | Homo sapiens WD repeat domain 47 (WDR47), transcript variant 3, mRNA. (from RefSeq NM_0011 | NM_001142551 |

| | | | | | | | | | | |
|---|---|---|---|---|---|---|---|---|---|---|
| uc060plz.1 | 8.10E-04 | DOWN | 1.4390806 | 1.4390806 | 0.5251474 | 0.4197742 | -0.1053732 | WDR7 | Homo sapiens WD repeat domain 7 (WDR7), transcript variant 2, mRNA. (from RefSeq NM_052834) | NM_052834 |
| uc062unn.1 | 5.56E-04 | DOWN | 2.5743792 | 2.5743792 | 1.3642246 | -1.6938831 | -3.0581076 | WHSC1 | Homo sapiens Wolf-Hirschhorn syndrome candidate 1 (WHSC1), transcript variant 3, mRNA. (from R | NM_133335 |
| uc059qwq.1 | 7.51E-04 | DOWN | 1.4578638 | 1.4578638 | 0.54385597 | 0.18383855 | -0.3600174 | ZC3H7A | Homo sapiens zinc finger CCCH-type containing 7A (ZC3H7A), mRNA. (from RefSeq NM_014153) | NM_014153 |
| uc060pua.1 | 2.15E-04 | DOWN | 1.431236 | 1.431236 | 0.5172616 | 1.455643 | 0.93838143 | ZCCHC2 | Homo sapiens zinc finger, CCHC domain containing 2 (ZCCHC2), transcript variant 1, mRNA. (from Re | NM_017742 |
| uc001knl.5 | 1.98E-04 | DOWN | 1.2404679 | 1.2404679 | 0.31088442 | -1.4266387 | -1.7375231 | ZDHHC16 | Homo sapiens zinc finger, DHHC-type containing 16 (ZDHHC16), transcript variant 2, mRNA. (from Re | NM_198043 |
| uc001knm.5 | 2.20E-04 | DOWN | 1.2567183 | 1.2567183 | 0.32966128 | -1.4043833 | -1.7340446 | ZDHHC16 | Homo sapiens zinc finger, DHHC-type containing 16 (ZDHHC16), transcript variant 4, mRNA. (from Re | NM_198045 |
| uc001knn.5 | 2.96E-04 | DOWN | 1.2478478 | 1.2478478 | 0.31944197 | -1.4134797 | -1.7329217 | ZDHHC16 | Homo sapiens zinc finger, DHHC-type containing 16 (ZDHHC16), transcript variant 3, mRNA. (from Re | NM_198044 |
| uc057vfd.1 | 6.44E-05 | DOWN | 1.6282582 | 1.6282582 | 0.7033295 | -0.3471101 | -1.0504397 | ZDHHC16 | Homo sapiens zinc finger, DHHC-type containing 16 (ZDHHC16), transcript variant 7, mRNA. (from Re | NM_001287804 |
| uc059ygx.1 | 3.41E-04 | DOWN | 1.5172886 | 1.5172886 | 0.6014955 | -0.2331409 | -0.8346365 | ZFPM1 | Homo sapiens zinc finger protein, FOG family member 1 (ZFPM1), mRNA. (from RefSeq NM_15381 | NM_153813 |
| uc010qpb.3 | 7.77E-04 | DOWN | 1.2197018 | 1.2197018 | 0.28652844 | -0.3717222 | -0.6582506 | ZFYVE27 | Homo sapiens zinc finger, FYVE domain containing 27 (ZFYVE27), transcript variant 6, mRNA. (from | NM_001174121 |
| uc061you.1 | 3.34E-05 | DOWN | 1.538955 | 1.538955 | 0.621951 | -0.3797532 | -1.0017042 | ZGPAT | Homo sapiens zinc finger, CCCH-type with G patch domain (ZGPAT), transcript variant 3, mRNA. (fro | NM_181485 |
| uc004aum.2 | 2.64E-04 | DOWN | 1.3094375 | 1.3094375 | 0.38894722 | -0.0145963 | -0.4035435 | ZNF169 | Homo sapiens zinc finger protein 169 (ZNF169), transcript variant 2, mRNA. (from RefSeq NM_1943 | NM_194320 |
| uc010tbw.3 | 2.49E-04 | DOWN | 1.4636956 | 1.4636956 | 0.54961556 | -2.0871649 | -2.6367805 | ZNF268 | Homo sapiens zinc finger protein 268 (ZNF268), transcript variant 5, mRNA. (from RefSeq NM_0011 | NM_001165883 |
| uc064fyo.1 | 6.80E-04 | DOWN | 1.4893813 | 1.4893813 | 0.5747132 | -1.0091217 | -1.5838348 | ZNF3 | Homo sapiens zinc finger protein 3 (ZNF3), transcript variant 1, mRNA. (from RefSeq NM_017715) | NM_017715 |
| uc002nvr.3 | 9.95E-04 | DOWN | 1.2547241 | 1.2547241 | 0.32737023 | -0.7882028 | -1.1155729 | ZNF302 | Homo sapiens zinc finger protein 302 (ZNF302), transcript variant 3, mRNA. (from RefSeq NM_0012 | NM_001289181 |
| uc060xsx.1 | 3.28E-04 | DOWN | 1.3566679 | 1.3566679 | 0.44006756 | -0.0882812 | -0.5283487 | ZNF383 | Homo sapiens zinc finger protein 383 (ZNF383), mRNA. (from RefSeq NM_152604) | NM_152604 |
| uc001ico.4 | 1.86E-04 | DOWN | 1.2566826 | 1.2566826 | 0.32962036 | -0.2125391 | -0.5421595 | ZNF496 | Homo sapiens zinc finger protein 496 (ZNF496), mRNA. (from RefSeq NM_032752) | NM_032752 |
| uc002mtq.3 | 1.41E-04 | DOWN | 1.4632297 | 1.4632297 | 0.5491562 | -0.7157932 | -1.2649494 | ZNF563 | Homo sapiens zinc finger protein 563 (ZNF563), mRNA. (from RefSeq NM_145276) | NM_145276 |
| uc060zmo.1 | 9.09E-05 | DOWN | 1.7175585 | 1.7175585 | 0.7803592 | 0.4081181 | -0.3722412 | ZNF576 | Homo sapiens zinc finger protein 576 (ZNF576), transcript variant 2, mRNA. (from RefSeq NM_0011 | NM_001145347 |
| uc061dnb.1 | 5.18E-04 | DOWN | 1.5809321 | 1.5809321 | 0.6607754 | 0.41471407 | -0.2460613 | ZNF586 | Homo sapiens zinc finger protein 586 (ZNF586), transcript variant 2, mRNA. (from RefSeq NM_0010 | NM_001077426 |
| uc061dnf.1 | 9.71E-04 | DOWN | 1.51933 | 1.51933 | 0.6034353 | -0.3240847 | -0.92752 | ZNF587 | Homo sapiens zinc finger protein 587 (ZNF587), transcript variant 1, mRNA. (from RefSeq NM_0328 | NM_032828 |
| uc061dng.1 | 9.64E-04 | DOWN | 1.7451968 | 1.7451968 | 0.8033897 | -0.2446956 | -1.0480853 | ZNF587 | Homo sapiens zinc finger protein 587 (ZNF587), transcript variant 2, mRNA. (from RefSeq NM_0012 | NM_001204817 |
| uc061dnh.1 | 5.39E-04 | DOWN | 1.4637096 | 1.4637096 | 0.54962933 | -0.3309494 | -0.8805787 | ZNF587 | Homo sapiens zinc finger protein 587 (ZNF587), transcript variant 2, mRNA. (from RefSeq NM_0012 | NM_001204817 |
| uc061dni.1 | 4.60E-04 | DOWN | 1.4697145 | 1.4697145 | 0.555536 | -0.5591287 | -1.1146647 | ZNF587 | Homo sapiens zinc finger protein 587 (ZNF587), transcript variant 2, mRNA. (from RefSeq NM_0012 | NM_001204817 |
| uc060weu.1 | 2.74E-04 | DOWN | 1.75261 | 1.75261 | 0.809505 | 0.68229747 | -0.1272075 | ZNF626 | Homo sapiens zinc finger protein 626 (ZNF626), transcript variant 2, mRNA. (from RefSeq NM_1452 | NM_145297 |
| uc059tkg.1 | 7.93E-04 | DOWN | 2.61518 | 2.61518 | 1.3869102 | -1.3806344 | -2.7675447 | ZNF668 | Homo sapiens zinc finger protein 668 (ZNF668), transcript variant 2, mRNA. (from RefSeq NM_0247 | NM_024706 |
| uc057fla.1 | 5.33E-04 | DOWN | 1.3146166 | 1.3146166 | 0.39464206 | -1.5912694 | -1.9859115 | ZNF691 | Homo sapiens zinc finger protein 691 (ZNF691), transcript variant 1, mRNA. (from RefSeq NM_0012 | NM_001242739 |
| uc011kuo.3 | 5.63E-04 | DOWN | 1.1895795 | 1.1895795 | 0.25045168 | 1.8345261 | 1.5840744 | ZNF783 | Homo sapiens zinc finger family member 783 (ZNF783), mRNA. (from RefSeq NM_001195220) | NM_001195220 |
| uc060tys.1 | 8.33E-04 | DOWN | 1.734782 | 1.734782 | 0.7947543 | 0.78073627 | -0.0140181 | ZNF799 | Homo sapiens zinc finger protein 799 (ZNF799), mRNA. (from RefSeq NM_001080821) | NM_001080821 |
| uc010ygn.3 | 5.09E-04 | DOWN | 1.2656769 | 1.2656769 | 0.3399091 | 1.6741399 | 1.3342308 | ZNF835 | Homo sapiens zinc finger protein 835 (ZNF835), mRNA. (from RefSeq NM_001005850) | NM_001005850 |
| uc061cbj.1 | 3.95E-04 | DOWN | 1.4309256 | 1.4309256 | 0.51694864 | 0.8439073 | 0.32695857 | ZNF841 | Homo sapiens zinc finger protein 841 (ZNF841), mRNA. (from RefSeq NM_001136499) | NM_001136499 |
| uc062iuy.1 | 4.36E-04 | DOWN | 1.3225425 | 1.3225425 | 0.40331414 | -0.3897847 | -0.7930989 | ZNF852 | Homo sapiens zinc finger protein 852 (ZNF852), mRNA. (from RefSeq NM_001287349) | NM_001287349 |
| uc062iuz.1 | 2.11E-05 | DOWN | 1.3880543 | 1.3880543 | 0.47306395 | -0.2150409 | -0.6881048 | ZNF852 | Homo sapiens zinc finger protein 852 (ZNF852), mRNA. (from RefSeq NM_001287349) | NM_001287349 |
| uc002bkr.4 | 3.45E-05 | DOWN | 1.346895 | 1.346895 | 0.42963737 | -1.5751795 | -2.0048168 | ZSCAN2 | Homo sapiens zinc finger and SCAN domain containing 2 (ZSCAN2), transcript variant 1, mRNA. (fro | NM_181877 |
| uc059mrx.1 | 3.03E-05 | DOWN | 1.3308383 | 1.3308383 | 0.4123353 | -1.501401 | -1.9137362 | ZSCAN2 | Homo sapiens zinc finger and SCAN domain containing 2 (ZSCAN2), transcript variant 1, mRNA. (fro | NM_181877 |
| uc059mry.1 | 2.32E-04 | DOWN | 1.4622391 | 1.4622391 | 0.54817927 | -0.5980268 | -1.146206 | ZSCAN2 | Homo sapiens zinc finger and SCAN domain containing 2 (ZSCAN2), transcript variant 1, mRNA. (fro | NM_181877 |
| uc059msa.1 | 1.76E-04 | DOWN | 1.2959998 | 1.2959998 | 0.37406546 | -1.1061901 | -1.4802556 | ZSCAN2 | Homo sapiens zinc finger and SCAN domain containing 2 (ZSCAN2), transcript variant 1, mRNA. (fro | NM_181877 |
| uc032wpp.2 | 6.69E-04 | DOWN | 1.255474 | 1.255474 | 0.3282321 | -0.3200883 | -0.6483204 | ZSCAN26 | Homo sapiens zinc finger and SCAN domain containing 26 (ZSCAN26), transcript variant d, mRNA. (f | NM_001287421 |
| uc059pye.1 | 7.08E-04 | DOWN | 1.2240224 | 1.2240224 | 0.29162994 | -1.0684617 | -1.3600916 | ZSCAN32 | Homo sapiens zinc finger and SCAN domain containing 32 (ZSCAN32), transcript variant 3, mRNA. (f | NM_001284528 |

**Supplementary Table 4. Summary of published manuscripts linking progenitor cell changes to atherosclerosis.**

| First | Last | Journal | Year | Source | Method | | | Endnote | DOI/PMID |
|---|---|---|---|---|---|---|---|---|---|
| Vasa et al | Dimmeler | Circ Res | 2001 | blood | EPC isolated by adhesion | KDR+/CD34+ by flow | EPC reduced in CAD, - correlation w risks | | 10.1161/hh1301.093953 |
| D'Ippolito | Schiller | J. Cell Science | 2004 | marrow | MIAMI, marrow isolated adult multilineage indu | adhesion | CD81 positive, CD34 neg! | | 10.1242/jcs.01103 |
| Damani | Topol | Sci. Trans Med | 2012 | blood | 3 channel microscopy | Circ Endo Cells | multinuclear predict acute MI | | 10.1126/scitranslmed.3003451 |
| Bielak | Peyser | Clinical Medicine | 2009 | blood | flow | CD34+ cell count | correlates with Ca score | | |
| Hill | Finkel | NEJM | 2003 | blood | colony forming units from 45 men | EPC, vascular function, cv risk (-0.47) and vasodilation (0. | | 25966 | 10.1056/NEJMoa022287 |
| Schmidt-Lucke | Dimmeler | Circulation | 2005 | blood | flow for CD34+/KDR+ | reduced EPC associated with higher events, 43 con vs 44 with stable cad | | | |
| Schmidt-Lucke | Dimmeler | Atherosclerosis | 2010 | blood | flow for CD34+/KDR+ and apop EPC | statins, not ezetimibe reduced EPC apop | | | |
| Hristov | Weber | Atherosclerosis | 2007 | blood | flow for CD34+/KDR+ | statins reduced EPC numbers | | | |
| Schmidt-Lucke | Dimmeler | PLOS One | 2010 | blood | ISHAGE gating on flow for CD45(dim)/CD34+/KI | confirm reduced EPC in CAD and statin effect | | 25967 | 10.1371/journal.pone.0013790 |
| Werner | Nickenig | NEJM | 2005 | blood | flow for CD34+/KDR+ | high EPC had lower event rate, but not predictive of MI | | | |
| Chen | Chang | Int Heart J | 2006 | blood | flow CD34+/KDR+ and CD62E or CD31 | no age related change in healthy subjects 20-61 | | | 16479045 |
| Laufs | | Circulation | 2004 | blood | | physical training increases EPC numbers | | | |
| Heiss | | JACC | 2005 | blood | | EPC in age related dysfunction | | | |
| Kunz | | Am Ht Journal | 2006 | blood | | EPC predict CAD severity | | | |
| Fadini | | Stroke | 2006 | blood | flow CD34+/KDR+ | EPC predicts subclinical atherosclerosis | | | |
| Rauscher | | Circulation | 2003 | blood | BM EPC treatment prevents athero in Apo E mice | | | | 10.1161/01.CIR.0000082924.75945.48 |
| Fadini | Werner | PLOS One | 2010 | blood | meta-analysis of 1,057 patients | CPC helped predict MACE in high risk patients | | | 10.1371/journal.pone.0011488 |
| Pirro | Mannarino | Atherosclerosis | 2008 | blood | flow CD34+/KDR+ | EPC, and EPC microparticles reduced with high risk | | | |
| Bonello | Sabatier | J Thromb Haem | 2012 | blood | flow CD34+/KDR+ | EPC changes, not level, predict restenosis after BMS | | | 10.1111/j.1538-7836.2012.04854.x |
| Chen | Zhu | Clinical Science | 2004 | blood | adhesion to FN | EPC levels lower in hypercholesterolemia | | | |
| Muller-Ehmsen | Flesch | Eur Heart Jour | 2008 | blood | CD34 and KDR, and AC133 | CPC lower in obesity | | | 10.1093/eurheartj/ehn213 |
| Boos | Lip | J Thromb Haem | 2008 | blood | immunobeads, vWF, sEel, sFas, sFasL Elisa | CEC associated with MACE and CV death | | | 10.1111/j.1538-7836.2008.03148.x |
| **Arrays** | | | | | | | | | |
| Liu et al | | Physiol Gen | 2011 | | CD34+ immunomagnetic, Isolex300i | CD34+ differentiated with cytokines | | | 10.1152/physiolgenomics.00099.2011 |
| Ahrens | Agrotis | PLOS One | 2011 | | EPC isolation improved by culture method | Cord blood | elevated Il-37, PDK4, A2M | | 10.1371/journal.pone.0023210 |
| Cheng | Lee | BMC Medical Ger | 2012 | leukocyt | divided by high risk, ie FH, vs low risk | found 56 biomarkers of risk | | | http://www.biomedcentral.com/1755-8794/5/2 |
| Huang CC | MESA | Physiol Genomics | 2011 | PBL | females divided by Ca score, about 300 | 409 transcripts | found race associate genes also | | 10.1152/physiolgenomics.00243.2010 |
| Colombo | Marina Came | Thr. Res | 2011 | platelets | stable angina vs non ST coronary | 45 transcripts | found GP1BB | | 10.1016/j.thromres.2011.02.012 |
| Brinch | Kolvraa | Prenatal Diagnosi | 2012 | materna | magnetic sorting, microarray | 28 transcripts | mmp14, mcam, kcnq4, cldn6, F3 | | |
| Kraan | Horrevoets | BMC Genomics | 2010 | blood | magnetic beads for CD34, CD14, CD4, 60 ml | Illumina BeadChip | retinoic acid signature | | http://www.biomedcentral.com/1471-2164/11/388 |

**Supplementary Table 5. Selected manuscripts reporting an association of Treg abundance with CAD.**

| Authors | Year | Citation | DOI | Treg % * Normals | Stable Angina | CAD or Unstable | AMI | %Change | | FoxP3 | | Ethnicity |
|---|---|---|---|---|---|---|---|---|---|---|---|---|
| Cheng, Yu et al | 2008 | Clin. Immun. 127:89-87 | 10.1016/j.clim.2008.01.009 | none | 0.5 | 3 | 3.4 | down | no normals | down >2x in PBMC | | Chinese |
| Li et al | 2014 | Cell Biochem Biophys 70:1489-98 | 10.1007/s12013-014-9993-5 | 5.2 | 4.8 | 3.8 | 3 | 27.00% | N v UA | | | Chinese |
| Emoto et al | 2014 | Circulation J. 78:2935-2941 | 10.1253/circj.CJ-14-0644 | 4.2 | | 3.1 | | 29.00% | | | | Japanese |
| Mengya et al | 2013 | Clin Rheum 32:1045 | 10.1007/s10067-013-2237-z | 1 | | 0.6 | | 40.00% | | | | Chinese |
| Liu et al | 2012 | Inflamm Res 61:1155-1165 | 10.1007/s00011-012-0510-2 | 6.8 | 4.7 | 2.2 | | 31.00% | neg v stable | down >2x | | Chinese |
| Amirati et al | 2010 | Arterio Throm Vasc Biol 30:1832-1841 | 10.1161/ATVBAHA.110.206813 | 5.1 | | 4.3 | | 16.00% | con vs ACS | | | Italy |
| Zhang et al | 2012 | JBC 287(41): 34157-34166 | 10.1074/jbc.M112.382978 | 5.05 | | 3.98 | | 22.00% | con vs ACS | | | Chinese |
| Potekina et al | 2015 | Atherosclerosis 238:17-21 | 10.1016/j.atherosclerosis.2014.10.088 | 5.1 | | 4.2 | | 18.00% | con v UA | down | | Russian |
| Han et al | 2007 | Clin Immunal 124: 90-97 | 10.1016/j.clim.2007.03.546 | 5.76 | | 3.4 | | 59.00% | con v UA | down | | Chinese |
| | | | | | | | | | **Fold** | | | |
| | | | | 4.78 | 4.75 | 3.20 | | 30.25% | 1.43369176 | **Average** | | |
| **Additional and Related studies** | | | | | | | | | | | | |
| Yazdani et al | 2016 | Iran J Immunol | IJIv13i4A2 | | | | | | | | | Iran |
| Yang et al | 2016 | Am J Trans Res | | 5.21 | 4.02 | | | | | | | China |
| Yang et al | 2016 | Am J Trans Res | | 4.95 | 2.89 | | | | | down ~2X | FoxP3 and CD4 posit | |
| Huang et al | 2017 | Clin Exp Immunol | 10.1111/cei.12897 | | | | | | | | | China |
| Afanasieva et al | 2016 | Ter Arkh | 10.17116/terarkh201688931-38 | | | | | | | | | Russian |
| Del Porta et al | 2017 | Ann Med | 10.1080/07853890.2016.1241427 | | | | | | | | | Italy |

**Supplementary Table 6. Correlations of TRACs with known Treg markers.**

| Gene Symbo | FOXP3 | PSORS | AHRR | DLG1 | IL12A | ICOSLG | PDCD1 | CD88 | TRIM28 | TCF3 | CD68 | IKZF4 | DGKA | CD27 | B3GAT1 | SMYD3 | B3GAT1 | ICOS | PTPRC | RUNX1 | ICOS | CD8A | CD4 | CD27 | IL2RA | |
|---|---|---|---|---|---|---|---|---|---|---|---|---|---|---|---|---|---|---|---|---|---|---|---|---|---|---|
| IL2RA | 0.220 | 0.135 | 0.035 | 0.166 | -0.240 | 0.179 | -0.011 | 0.160 | 0.085 | 0.130 | 0.211 | 0.080 | 0.294 | 0.368 | -0.111 | 0.111 | 0.061 | 0.596 | 0.100 | 0.413 | 0.619 | 0.177 | 0.184 | 0.285 | 1 | IL2RA |
| CD27 | 0.083 | -0.106 | 0.006 | -0.099 | -0.081 | 0.255 | 0.272 | 0.115 | 0.163 | 0.133 | 0.349 | -0.269 | 0.072 | 0.594 | 0.300 | -0.129 | 0.240 | 0.233 | -0.345 | 0.214 | 0.189 | 0.214 | 0.504 | 1.000 | | CD27 |
| CD4 | -0.073 | -0.237 | -0.202 | -0.128 | -0.229 | 0.066 | 0.109 | 0.092 | 0.198 | -0.150 | 0.606 | -0.176 | 0.078 | 0.363 | 0.065 | -0.087 | -0.103 | 0.311 | -0.158 | 0.167 | 0.273 | 0.093 | 1.000 | | | CD4 |
| CD8A | 0.534 | 0.056 | 0.380 | 0.245 | 0.009 | 0.299 | 0.390 | 0.517 | 0.430 | 0.031 | 0.266 | 0.042 | 0.114 | 0.462 | 0.065 | 0.062 | 0.224 | 0.150 | -0.212 | 0.358 | 0.075 | 1.000 | | | | CD8A |
| ICOS | 0.115 | 0.167 | 0.024 | 0.272 | -0.263 | 0.024 | -0.090 | 0.473 | 0.156 | 0.006 | 0.187 | 0.039 | 0.468 | 0.261 | -0.128 | 0.091 | -0.097 | 0.874 | 0.223 | 0.171 | 1.000 | | | | | ICOS |
| RUNX1 | 0.363 | -0.029 | 0.084 | 0.044 | -0.057 | 0.189 | 0.113 | 0.012 | -0.037 | -0.311 | 0.499 | -0.078 | -0.142 | 0.099 | 0.105 | 0.129 | 0.484 | 0.247 | -0.185 | 1.000 | | | | | | RUNX1 |
| PTPRC | -0.309 | 0.035 | -0.188 | 0.235 | -0.191 | -0.214 | -0.459 | 0.095 | -0.042 | -0.015 | -0.086 | 0.228 | 0.227 | -0.028 | -0.476 | 0.113 | -0.449 | 0.050 | 1.000 | | | | | | | PTPRC |
| ICOS | 0.231 | 0.179 | 0.059 | 0.266 | -0.214 | 0.041 | 0.066 | 0.412 | 0.134 | -0.057 | 0.203 | 0.011 | 0.351 | 0.280 | 0.031 | 0.054 | 0.053 | 1.000 | | | | | | | | ICOS |
| B3GAT1 | 0.353 | 0.107 | 0.299 | -0.136 | 0.042 | 0.069 | 0.349 | -0.181 | -0.107 | -0.118 | 0.176 | -0.129 | -0.241 | -0.149 | 0.638 | 0.017 | 1.000 | | | | | | | | | B3GAT1 |
| SMYD3 | 0.082 | 0.263 | 0.030 | 0.196 | -0.086 | -0.065 | -0.091 | 0.123 | -0.015 | 0.004 | -0.079 | 0.002 | 0.042 | -0.044 | -0.034 | 1.000 | | | | | | | | | | SMYD3 |
| B3GAT1 | 0.165 | 0.098 | 0.244 | -0.209 | 0.083 | 0.105 | 0.589 | -0.091 | -0.119 | -0.106 | 0.003 | -0.147 | -0.253 | -0.166 | 1.000 | | | | | | | | | | | B3GAT1 |
| CD27 | 0.187 | -0.031 | 0.090 | 0.212 | -0.186 | 0.268 | 0.052 | 0.416 | 0.518 | 0.216 | 0.305 | -0.011 | 0.387 | 1.000 | | | | | | | | | | | | CD27 |
| DGKA | 0.178 | 0.042 | 0.341 | 0.288 | -0.311 | 0.330 | -0.034 | 0.382 | 0.346 | 0.197 | -0.073 | 0.214 | 1.000 | | | | | | | | | | | | | DGKA |
| IKZF4 | 0.130 | 0.254 | 0.197 | 0.182 | -0.003 | 0.039 | -0.022 | 0.028 | 0.291 | 0.218 | -0.189 | 1.000 | | | | | | | | | | | | | | IKZF4 |
| CD68 | 0.075 | -0.093 | -0.140 | -0.118 | -0.163 | 0.020 | 0.081 | 0.141 | 0.169 | -0.163 | 1.000 | | | | | | | | | | | | | | | CD68 |
| TCF3 | 0.061 | 0.189 | 0.163 | 0.176 | -0.127 | 0.121 | 0.096 | 0.108 | 0.308 | 1.000 | | | | | | | | | | | | | | | | TCF3 |
| TRIM28 | 0.213 | 0.010 | 0.208 | 0.085 | -0.171 | 0.207 | 0.180 | 0.394 | 1.000 | | | | | | | | | | | | | | | | | TRIM28 |
| CD8B | 0.203 | 0.186 | 0.224 | 0.412 | -0.176 | 0.076 | 0.075 | 1.000 | | | | | | | | | | | | | | | | | | CD8B |
| PDCD1 | 0.480 | 0.186 | 0.444 | 0.086 | 0.135 | 0.288 | 1.000 | | | | | | | | | | | | | | | | | | | PDCD1 |
| ICOSLG | 0.407 | 0.010 | 0.387 | 0.088 | 0.000 | 1.000 | | | | | | | | | | | | | | | | | | | | ICOSLG |
| IL12A | 0.151 | 0.080 | 0.164 | -0.058 | 1.000 | | | | | | | | | | | | | | | | | | | | | IL12A |
| DLG1 | 0.315 | 0.283 | 0.279 | 1.000 | | | | | | | | | | | | | | | | | | | | | | DLG1 |
| AHRR | 0.722 | 0.480 | 1.000 | | | | | | | | | | | | | | | | | | | | | | | AHRR |
| PSORS1C1 | 0.352 | 1.000 | | | | | | | | | | | | | | | | | | | | | | | | PSORS1C1 |
| FOXP3 | 1.000 | | | | | | | | | | | | | | | | | | | | | | | | | FOXP3 |
| correlation w | FOXP3 | PSORS | AHRR | DLG1 | IL12A | ICOSLG | PDCD1 | CD88 | TRIM28 | TCF3 | CD68 | IKZF4 | DGKA | CD27 | B3GAT1 | SMYD3 | B3GAT1 | ICOS | PTPRC | RUNX1 | ICOS | CD8A | CD4 | CD27 | IL2RA | |

**Supplementary Table 7. DEG common to Discovery and Validation Cohorts.**

| UCSC ID | p ([CAD] Vs | Regulation ([ | FC (abs) ([CA | FC ([CAD] Vs | Log FC ([CAD | [CAD](raw) | [LOW](raw) | start of trans | Description | chr | SeqLL TRACIT3 INOVA HG38 SeqLL Gene Name Gen80 664 list | mat | STRESS | SeqLL TRACIT1 GWU HG38 SeqLL CathDx 599 list | SeqLL TRACIT1 GWU HG19 Seqll CathDx |
|---|---|---|---|---|---|---|---|---|---|---|---|---|---|---|---|
| uc061hhh.1 | 0.00466694 | up | 3.1491892 | 3.1491892 | 1.6549804 | 0.08496023 | 0.02697844 | 26308175 | Adhesion G Protein-Coupled Receptor F3. (from Un | chr2 | ADGRF3 | c | | ADGRG3 | |
| uc063gxp.1 | 0.00544914 | down | 1.2519302 | -1.2519302 | -0.3241542 | 4.696951 | 5.8802547 | 132897203 | AF4/FMR2 family. member 4 (from HGNC AFF4) | chr5 | AFF4 | c | | AFF3 | |
| uc061wik.1 | 0.00476174 | down | 1.4414719 | -1.4414719 | -0.5275428 | 0.92420864 | 1.3322208 | 34291464 | adenosylhomocysteinase (from HGNC AHCY) | chr20 | AHCY | a | | | AHCYL2 |
| uc062bvu.1 | 8.58E-04 | down | 9.211627 | -9.211627 | -3.203456 | 0.0066789 | 0.06152349 | 20965176 | apoptosis-inducing factor. mitochondrion-associate | chr22 | AIFM3 | a | | | AIFM3 |
| uc062eav.1 | 1.45E-04 | down | 9.639785 | -9.639785 | -3.269001 | 0.03060729 | 0.29504773 | 37833008 | Ankyrin Repeat Domain 54. (from UniProt C9JX82) | chr22 | ANKRD54 | a | ANKHD1 | | ANKRD54 |
| uc057shp.1 | 0.00793906 | down | 1.3702843 | -1.3702843 | -0.4544753 | 0.50020045 | 0.6854169 | 24629013 | Rho GTPase activating protein 21 (from HGNC ARH | chr10 | ARHGAP21 | a | | | ARHGAP22 |
| uc060lgs.1 | 0.00771698 | down | 4.540955 | -4.540955 | -2.1829958 | 0.037111 | 0.16851938 | 81097578 | BAI1 Associated Protein 2. (from UniProt I3L0Y9) | chr17 | BAIAP2 | e | | BAIAP2 | BAIAP3 |
| uc059uld.1 | 0.00893112 | down | 1.2283325 | -1.2283325 | -0.2967012 | 2.237617 | 2.7485378 | 56484344 | Bardet-Biedl syndrome 2 (from HGNC BBS2) | chr16 | BBS2 | a | | | BBS2 |
| uc061hmb.1 | 0.00518361 | down | 4.071977 | -4.071977 | -2.0257294 | 0.0617337 | 0.2513782 | 27241920 | Carbamoyl-Phosphate Synthetase 2, Aspartate, Bel | chr2 | CAD | a | | | CAD |
| uc058qob.1 | 0.00997198 | up | 3.9418516 | 3.9418516 | 1.9788735 | 0.45921156 | 0.11649641 | 67269717 | cullin-associated and neddylation-dissociated 1 (frc | chr12 | CAND1 | a | | | CAND2 |
| uc058svy.1 | 0.00836163 | down | 1.2155546 | -1.2155546 | -0.2816147 | 3.5544074 | 4.320576 | 108658880 | Belongs to the WD repeat coronin family. (from Ur | chr12 | CORO1C | a | CORO1B | | CORO6 |
| uc058zuz.1 | 0.00311815 | down | 3.6590462 | -3.6590462 | -1.8714676 | 0.00556233 | 0.02035282 | 24071588 | copine VI (neuronal) (from HGNC CPNE6) | chr14 | CPNE6 | a | | | CPNE3 |
| uc061oas.1 | 0.00893666 | down | 1.251643 | -1.251643 | -0.3238231 | 2.9431279 | 3.6837454 | 135906823 | aspartyl-tRNA synthetase (from HGNC DARS) | chr2 | DARS | a | hypoxia | | DARS |
| uc062lxl.1 | 0.00727313 | down | 3.096001 | -3.096001 | -1.6304059 | 0.01418335 | 0.04391168 | 98807910 | discoidin. CUB and LCCL domain containing 2 (from | chr3 | DCBLD2 | c | | DCBLD1 | |
| uc063naw.1 | 0.00208251 | down | 1.2229795 | -1.2229795 | -0.2904003 | 5.738689 | 7.018299 | 31530242 | DEAD (Asp-Glu-Ala-Asp) box polypeptide 39B (fror | chr6 | DDX39B | a | | | DDX39A |
| uc063hco.1 | 0.00609706 | down | 5.3292775 | -5.3292775 | -2.41394 | 0.0663546 | 0.35362202 | 134773784 | DEAD (Asp-Glu-Ala-Asp) box polypeptide 46 (from | chr5 | DDX46 | c | DDX47 | | DDX42 |
| uc058viq.1 | 0.00960035 | down | 2.0436409 | -2.0436409 | -1.0311416 | 0.44386658 | 0.90710384 | 132139112 | DEAD (Asp-Glu-Ala-Asp) box polypeptide 51 (from | chr12 | DDX51 | a | DDX50 | | DDX51 |
| uc058pcp.1 | 0.00954773 | up | 1.4104319 | 1.4104319 | 0.49613696 | 4.2960267 | 3.0458946 | | diacylglycerol kinase, alpha 80kDa (from HGNC DGI | chr12 | DGKA | | | DGKA | DGKA |
| uc002app.4 | 0.00721339 | down | 1.2387679 | -1.2387679 | -0.3089059 | 1.1705571 | 1.4500486 | 66293216 | DIS3 like exosome 3'-5' exoribonuclease (DIS3L). tr | chr15 | DIS3L | e | DIS3L2 | | DIS3 |
| uc064ljz.1 | 0.00664018 | down | 1.44623 | -1.44623 | -0.5322971 | 1.3436074 | 1.9431655 | 26627214 | dihydropyrimidinase-like 2 (from HGNC DPYSL2) | chr8 | DPYSL2 | a | DPYSL2 | | DPYSL3 |
| uc063pfd.1 | 0.00451893 | down | 5.760368 | -5.760368 | -2.526161 | 0.03775852 | 0.21750295 | 56617199 | Dystonin. (from UniProt H0YAT7) | chr6 | DST | o | DST | | |
| uc058pzo.1 | 7.35E-04 | down | 1.4873667 | -1.4873667 | -0.5727603 | 0.60503316 | 0.8999062 | 57604638 | Deltex E3 Ubiquitin Ligase 3. (from UniProt F8VUZ4 | chr12 | DTX3 | c | DTX3L | DTX1 | |
| uc059lkd.1 | 0.00922576 | down | 1.421449 | -1.421449 | -0.5073623 | 0.8373783 | 1.1902905 | 74655793 | Enhancer Of MRNA Decapping 3. (from UniProt H3I | chr15 | EDC3 | e | EDC3 | EDC3 | |
| uc064lnm.1 | 0.00821887 | down | 1.253947 | -1.253947 | -0.3264764 | 2.252565 | 2.824597 | 28118897 | elongator acetyltransferase complex subunit 3 (fro | chr8 | ELP3 | c | | ELP2 | |
| uc058bhy.1 | 0.00430335 | down | 1.3011535 | -1.3011535 | -0.3797912 | 4.9715977 | 6.468812 | 47732481 | formin binding protein 4 (from HGNC FNBP4) | chr11 | FNBP4 | e | | FNBP4 | |
| uc062llr.1 | 0.00234395 | down | 1.2440604 | -1.2440604 | -0.3150565 | 7.420352 | 9.231366 | 70968847 | forkhead box P1 (from HGNC FOXP1) | chr3 | FOXP1 | e | | FOXP1 | FOXP1, 1132 |
| uc002utu.4 | 0.00690295 | down | 1.2726772 | -1.2726772 | -0.3478665 | 1.6410824 | 2.0885682 | 196780088 | general transcription factor IIIC subunit 3 (GTF3C3) | chr2 | GTF3C3 | a | | GTF2H4 | GTF3C3 |
| uc058igy.1 | 0.00498888 | down | 1.2429291 | -1.2429291 | -0.313744 | 0.8698461 | 1.0811571 | 119131438 | histone H4 transcription factor (from HGNC HINFP) | chr11 | HINFP | e | | HINFP | |
| uc059vpb.1 | 0.00656629 | down | 5.5908747 | -5.5908747 | -2.483074 | 0.01402885 | 0.07843354 | 67165524 | Heat Shock Transcription Factor 4. (from UniProt H | chr16 | HSF4 | a | Heat Shock | | HSF4 |
| uc061qzm.1 | 0.00504353 | down | 1.420102 | -1.420102 | -0.5059946 | 2.0935285 | 2.9730241 | 197496890 | Heat Shock Protein Family D (Hsp60) Member 1 (ch | chr2 | HSPD1 | c | HSPD1 | HSPE1 | HSPB3 |
| uc059mbz.1 | 0.00979137 | down | 4.922826 | -4.922826 | -2.2994866 | 0.04578593 | 0.22539617 | 78163715 | Isocitrate Dehydrogenase 3 (NAD(+)) Alpha. (from | chr15 | IDH3A | a | | | IDH1 |
| uc001vnf.2 | 0.00700541 | down | 1.237847 | -1.237847 | -0.307833 | 1.0729606 | 1.328161 | 97976631 | Importin 5 | chr13 | IPO5 | c | | IPO11 | |
| uc057sqt.1 | 0.0056646 | down | 1.2380247 | -1.2380247 | -0.3080401 | 8.723372 | 10.799751 | 32929984 | Integrin Subunit Beta 1. (from UniProt C9JNE0) | chr10 | ITGB1 | a | ITGB1 | | ITGB1 |
| uc058nzx.1 | 1.30E-04 | down | 1.5037236 | -1.5037236 | -0.5885394 | 1.5619553 | 2.3487492 | 51048312 | LETM1 domain containing 1 (from HGNC LETMD1) | chr12 | LETMD1 | a | | | LETMD1 |
| uc001fnf.3 | 0.00678139 | down | 1.2709522 | -1.2709522 | -0.3459098 | 1.1653022 | 1.4810433 | 156082577 | lamin A/C (LMNA). transcript variant 2. mRNA. (frc | chr1 | LMNA | a | LMNA | | LMNA |
| uc064sik.1 | 0.00679545 | down | 1.3376278 | -1.3376278 | -0.4196767 | 2.030512 | 2.7160695 | 20346405 | MLLT3 Super Elongation Complex Subunit. (from U | chr9 | MLLT3 | a | | | MLLT3 |
| uc002aze.3 | 0.0059149 | down | 1.316997 | -1.316997 | -0.3972521 | 1.3843883 | 1.8232353 | 74890041 | mannose phosphate isomerase (MPI). transcript va | chr15 | MPI | a | | | MPI |
| uc057skx.1 | 0.00291165 | down | 1.2832036 | -1.2832036 | -0.3597501 | 3.3767724 | 4.3330865 | 28054142 | Membrane Palmitoylated Protein 7. (from UniProt | chr10 | MPP7 | a | | | MPP2 |
| uc060bvl.1 | 0.00599846 | down | 1.3389245 | -1.3389245 | -0.4210746 | 1.2934629 | 1.7318492 | 17179557 | Myosin Phosphatase Rho Interacting Protein. (from | chr17 | MPRIP | a | stress fibers | | MPRIP |
| uc057uah.1 | 0.00393548 | down | 1.2238083 | -1.2238083 | -0.2913776 | 4.7361636 | 5.796156 | 73250740 | mitochondrial ribosomal protein S16 (from HGNC N | chr10 | MRPS16 | c | | MRPS31 | |
| uc061ipi.1 | 0.00567156 | down | 5.6448393 | -5.6448393 | -2.4969325 | 0.03140524 | 0.17727755 | 42568167 | metastasis associated 1 family. member 3 (from H | chr2 | MTA3 | a | | | MTA3 |
| uc059cjt.1 | 0.0030653 | down | 6.661544 | -6.661544 | -2.7358565 | 0.02530567 | 0.16857481 | 64457996 | methylenetetrahydrofolate dehydrogenase (NADP+ | chr14 | MTHFD1 | e | MTHFD1 | MTHFD1 | MTHFD1L |
| uc061jge.1 | 0.00261511 | down | 1.259208 | -1.259208 | -0.3325166 | 1.658834 | 2.088817 | 55236620 | Mitochondrial Translational Initiation Factor 2 | chr2 | MTIF2 | a | hypoxia down | 292270 | MTIF3 |
| uc003wxt.4 | 0.00769938 | down | 2.4843318 | -2.4843318 | -1.3128579 | 0.05885801 | 0.14622281 | 17643803 | microtubule associated tumor suppressor 1 (MTUS | chr8 | MTUS1 | a | | | MTUS2 |
| uc059zfb.1 | 0.00387835 | down | 5.238711 | -5.238711 | -2.389212 | 0.0739109 | 0.38719785 | 1468222 | myosin IC (from HGNC MYO1C) | chr17 | MYO1C | e | MYO6 | MYO1E | MYO1D |
| uc061cul.1 | 0.0098469 | down | 4.928974 | -4.928974 | -2.3012874 | 0.03456391 | 0.1703646 | 54973993 | NLR Family Pyrin Domain Containing 2. (from UniP | chr19 | NLRP2 | a | aging | | NLRP2, |

| | | | | | | | | | | | | | | |
|---|---|---|---|---|---|---|---|---|---|---|---|---|---|---|
| uc002rcy.4 | 0.00504953 | down | 1.4440056 | -1.4440056 | -0.5300763 | 0.27387014 | 0.39547002 | 18555544 | NT5C1B-RDH14 readthrough (NT5C1B-RDH14). trar | chr2 | NT5C1B-RDH | e | | NT5C3A | NT5C3B |
| uc004bve.4 | 0.0090192 | down | 1.2714772 | -1.2714772 | -0.3465056 | 0.6369028 | 0.8098074 | 128456918 | outer dense fiber of sperm tails 2 (ODF2). transcrip | chr9 | ODF2 | e | | ODF2L | ODF2 |
| uc058upy.1 | 0.00163712 | down | 1.3608307 | -1.3608307 | -0.4444875 | 0.9761837 | 1.3284208 | 122975241 | 2-oxoglutarate and iron-dependent oxygenase dom | chr12 | OGFOD2 | e | OGFOD1 | OGFOD2 | OGFOD2 |
| uc062htp.1 | 2.85E-04 | down | 11.307217 | -11.307217 | -3.499172 | 0.02170722 | 0.24544828 | 31683968 | oxysterol binding protein-like 10 (from HGNC OSBP | chr3 | OSBPL10 | e | OSBPL9 | OSBPL10 | |
| uc062axf.1 | 0.00935585 | down | 3.932247 | -3.932247 | -1.9753538 | 0.01675902 | 0.06590062 | 45849989 | Poly(RC) Binding Protein 3 Contains 3 KH domains | chr21 | PCBP3 | e | PCBP2, PCBP | PCBP1-AS1 | |
| uc061apt.1 | 0.00386926 | down | 5.2680926 | -5.2680926 | -2.3972807 | 0.02025118 | 0.1066851 | 48097764 | phospholipase A2. group IVC (cytosolic. calcium-inc | chr19 | PLA2G4C | o | | | PLA2G10, |
| uc002eso.5 | 0.00228795 | down | 1.5580505 | -1.5580505 | -0.639742 | 0.17257616 | 0.26888236 | 67277509 | pleckstrin homology and RhoGEF domain containin | chr16 | PLEKHG4 | a | | | PLEKHG4 |
| uc060epe.1 | 0.00396322 | down | 6.754754 | -6.754754 | -2.7559032 | 0.01532278 | 0.1035016 | 39072013 | plexin domain containing 1 (from HGNC PLXDC1) | chr17 | PLXDC1 | c | | PLXNC1 | PLXND1 |
| uc060qvd.1 | 0.00637629 | down | 1.200787 | -1.200787 | -0.2639802 | 5.5362716 | 6.6478825 | 1088147 | polymerase (RNA) II (DNA directed) polypeptide E. | chr19 | POLR2E | c | POLR2B | POLR2D | POLR3GL |
| uc060ihp.1 | 0.00965993 | down | 1.2412302 | -1.2412302 | -0.3117708 | 1.7525004 | 2.1752565 | 60600182 | Protein Phosphatase, Mg2+/Mn2+ Dependent 1D | chr17 | PPM1D | a | | | PPM1B |
| uc062rrj.1 | 0.00865548 | down | 1.2024927 | -1.2024927 | -0.2660281 | 4.875126 | 5.8623033 | 195519160 | Protein Phosphatase 1 Regulatory Inhibitor Subunit | chr3 | PPP1R2 | c | PPP1R8 | PPP1R13B | PPP1R10 |
| uc064zfq.1 | 0.00463741 | down | 1.4196897 | -1.4196897 | -0.5055756 | 0.43235695 | 0.6138127 | 49269842 | Glycogen-targeting subunit for protein phosphatas | chrX | PPP1R3F | a | | | PPP1CC |
| uc057ubg.1 | 0.0052754 | down | 3.086338 | -3.086338 | -1.6258961 | 0.15896747 | 0.49062735 | 73496286 | PPP3CB antisense RNA 1 (head to head) (from HGN | chr10 | PPP3CB-AS1 | e | | PPP3CB-AS | PPP6R3 |
| uc058nma.1 | 0.00124233 | down | 1.2204394 | -1.2204394 | -0.2874007 | 2.856492 | 3.4861758 | 49003809 | Protein Kinase AMP-Activated Non-Catalytic Subuni | chr12 | PRKAG1 | a | | PRKCE | PRKAG1 |
| uc002dzb.2 | 0.00304907 | down | 1.2940506 | -1.2940506 | -0.371894 | 1.6394578 | 2.1215413 | 30654108 | proline rich 14 (from HGNC PRR14) | chr16 | PRR14 | c | | PRR14L | |
| uc064wqo.1 | 0.001422 | down | 1.3874075 | -1.3874075 | -0.4723916 | 4.342514 | 6.0248365 | 131473593 | Proline Rich Coiled-Coil 2B. (from UniProt Q5JSZ9) | chr9 | PRRC2B | a | PRRC2B | | PRRC2B |
| uc057zit.1 | 0.00307177 | down | 1.4568127 | -1.4568127 | -0.5428155 | 1.6053652 | 2.3387163 | 14610853 | proteasome (prosome. macropain) subunit. alpha t | chr11 | PSMA1 | c | | PSMA3 | PSMA6 |
| uc058zoc.1 | 0.00158443 | down | 1.4917963 | -1.4917963 | -0.5770505 | 1.4193069 | 2.1173165 | 23016542 | Belongs to the peptidase T1B family. (from UniPro | chr14 | PSMB5 | a | PSMD2 | | PSMC5 |
| uc060eva.1 | 0.00102343 | down | 1.2573454 | -1.2573454 | -0.3303811 | 3.3179946 | 4.171865 | 39986608 | proteasome (prosome. macropain) 26S subunit. no | chr17 | PSMD3 | c | PSMD2 | PSMD6 | |
| uc062hhe.1 | 0.00257963 | down | 1.275671 | -1.275671 | -0.3512563 | 7.6515155 | 9.760817 | 18414948 | SATB homeobox 1 (from HGNC SATB1) | chr3 | SATB1 | c | AGE! | SATB2 | |
| uc002ubx.5 | 0.00848147 | down | 2.115307 | -2.115307 | -1.080867 | 0.1222189 | 0.25853053 | 161624425 | Solute carrier family 4 member 10 (SLC4A10). tran | chr2 | SLC4A10 | a | | | SLC5A10 |
| uc064ikc.1 | 0.00143057 | down | 1.2051876 | -1.2051876 | -0.2692577 | 1.2460814 | 1.5017617 | 138460548 | Tripartite Motif Containing 24. (from UniProt A0A0 | chr7 | TRIM24 | c | TRIM21 | TRIM47 | TRIM28 |
| uc061qdv.1 | 0.00461844 | up | 1.4030657 | 1.4030657 | 0.48858255 | 2.0478244 | 1.4595356 | 178528739 | TTN antisense RNA 1 (from HGNC TTN-AS1) | chr2 | TTN-AS1 | e | | TTN | TTN-AS1 Nv |
| uc064yux.1 | 0.00753835 | down | 1.2729685 | -1.2729685 | -0.3481967 | 1.1967776 | 1.5234601 | 47232865 | ubiquitin specific peptidase 11 (USP11). mRNA. (fr | chrX | USP11 | e | USP11 | USP11 | |
| uc062unl.1 | 0.00353547 | down | 1.3544748 | -1.3544748 | -0.4377335 | 0.5120621 | 0.69357514 | 1939280 | Wolf-Hirschhorn syndrome candidate 1 (from HGN | chr4 | WHSC1 | a | | | WHSC1 |
| uc059ftl.1 | 0.00441255 | down | 6.35512 | -6.35512 | -2.6679194 | 0.01124214 | 0.07144516 | 103702015 | X-ray repair complementing defective repair in Chi | chr14 | XRCC3 | a | | XRRA1 | XRCC3 |

**Supplementary Table 8. 26 Transcript exemplars common to both studies and to known stress granule transcripts.**

| STRESS GRANULE | | Discovery HG19 | Discovery HG38 | Validation HG38 | MATCH STRESS |
|---|---|---|---|---|---|
| ANKHD1 | | ANKRD54 | | ANKRD54 | c |
| CORO1B | | CORO6 | | CORO1C | c |
| DDX31 | | DDX39A | | DDX39B | c |
| DDX47 | | DDX42 | | DDX46 | c |
| DDX50 | | DDX51 | | DDX51 | c |
| DDX58 | | DDX51 | | DDX54 | c |
| DIS3L2 | | | DIS3 | DIS3L | e |
| DPYSL2 | | | DPYSL2 | DPYSL2 | e |
| DST | | DST | | DST | e |
| DTX3L | | | DTX1 | DTX3 | e |
| EDC3 | | | EDC3 | EDC3 | e |
| HSF4 | | HSF4 | | HSF4 | e |
| HSPD1 | | HSPB3 | HSPE1 | HSPD1 | e |
| ITGB1 | | ITGB1 | | ITGB1 | e |
| LMNA | | LMNA | | LMNA | e |
| MTHFD1 | | | MTHFD1 | MTHFD1 | e |
| OGFOD1 | | OGFOD2 | OGFOD2 | OGFOD2 | e |
| OSBPL9 | | | OSBPL10 | OSBPL10 | c |
| PCBP2, PCBP1 | | | PCBP1-AS1 | PCBP3 | c |
| POLR2B | | POLR3GL | POLR2D | POLR2E | c |
| PPP1R8 | | PPP1R10 | PPP1R13B | PPP1R2 | c |
| PRRC2B | | PRRC2B | | PRRC2B | e |
| PSMD2 | | | PSMD6 | PSMD3 | c |
| SATB1 | | | SATB2 | SATB1 | e |
| TRIM21 | | TRIM28 | TRIM47 | TRIM24 | c |
| USP11 | | | USP11 | USP11 | e |

**Supplementary Table 9. Cilia and/or synapse/immune synapse related transcripts**

| | | | | | | | | | | | | | | | |
|---|---|---|---|---|---|---|---|---|---|---|---|---|---|---|---|
| | | | | | | | | | | | SeqLL | | | SeqLL | SeqLL |
| | | | | | | | | | | | **TRACIT3** | | | TRACIT1 | **TRACIT1** |
| | | | | | | | | | | | **INOVA** | | | GWU | **GWU** |
| | | | | | | | | | | | **HG38** | | | **HG38** | **HG19** |
| | | | | | | | | | | | **SeqLL** | | | SeqLL | **Seqll** |
| **UCSC ID** | **p ([CAD] Vs** | **Regulation (** | **FC (abs) ([CA** | **FC ([CAD] Vs** | **Log FC ([CAD** | **[CAD](raw)** | **[LOW](raw)** | **start of trans** | **Description** | **chr** | **Gene Name** | **match** | **STRESS** | CathDx | **CathDx** |
| uc061hhh.1 | 0.00466694 | up | 3.1491892 | 3.1491892 | 1.6549804 | 0.08496023 | 0.02697844 | 26308175 | Adhesion G Protein-Coupled Receptor F3. (fro | chr2 | ADGRF3 | c | | ADGRG3 | |
| uc057shp.1 | 0.00793906 | down | 1.3702843 | -1.3702843 | -0.4544753 | 0.50020045 | 0.6854169 | 24629013 | Rho GTPase activating protein 21 (from HGNC | chr10 | ARHGAP21 | a | | | ARHGAP22 |
| uc059uld.1 | 0.00893112 | down | 1.2283325 | -1.2283325 | -0.2967012 | 2.237617 | 2.7485378 | 56484344 | Bardet-Biedl syndrome 2 (from HGNC BBS2) | chr16 | BBS2 | a | | | BBS2 |
| uc058zuz.1 | 0.00311815 | down | 3.6590462 | -3.6590462 | -1.8714676 | 0.00556233 | 0.02035282 | 24071588 | copine VI (neuronal) (from HGNC CPNE6) | chr14 | CPNE6 | a | | | CPNE3 |
| uc062lxl.1 | 0.00727313 | down | 3.096001 | -3.096001 | -1.6304059 | 0.01418335 | 0.04391168 | 98807910 | discoidin. CUB and LCCL domain containing 2 ( | chr3 | DCBLD2 | c | | DCBLD1 | |
| uc064ljz.1 | 0.00664018 | down | 1.44623 | -1.44623 | -0.5322971 | 1.3436074 | 1.9431655 | 26627214 | dihydropyrimidinase-like 2 (from HGNC DPYSL | chr8 | DPYSL2 | a | DPYSL2 | | DPYSL3 |
| uc063pfd.1 | 0.00451893 | down | 5.760368 | -5.760368 | -2.526161 | 0.03775852 | 0.21750295 | 56617199 | Dystonin. (from UniProt H0YAT7) | chr6 | DST | o | DST | * | |
| uc003wxt.4 | 0.00769938 | down | 2.4843318 | -2.4843318 | -1.3128579 | 0.05885801 | 0.14622281 | 17643803 | microtubule associated tumor suppressor 1 (M | chr8 | MTUS1 | a | | | MTUS2 |
| uc059zfb.1 | 0.00387835 | down | 5.238711 | -5.238711 | -2.389212 | 0.0739109 | 0.38719785 | 1468222 | myosin IC (from HGNC MYO1C) | chr17 | MYO1C | e | MYO6 | MYO1E | MYO1D |
| uc004bve.4 | 0.0090192 | down | 1.2714772 | -1.2714772 | -0.3465056 | 0.6369028 | 0.8098074 | 128456918 | outer dense fiber of sperm tails 2 (ODF2). trar | chr9 | ODF2 | e | | ODF2L | ODF2 |
| uc064wqo.1 | 0.001422 | down | 1.3874075 | -1.3874075 | -0.4723916 | 4.342514 | 6.0248365 | 131473593 | Proline Rich Coiled-Coil 2B. (from UniProt Q5J | chr9 | PRRC2B | a | PRRC2B | | PRRC2B |
| uc001bse.3 | 0.00488237 | down | 3.3408244 | -3.3408244 | -1.7402041 | 0.07455237 | 0.24906638 | 30873134 | Syndecan 3 (SDC3). mRNA. (from RefSeq NM_ | chr1 | SDC3 | o | | | |

**Supplementary Table 10.**
**The expression of CardioDx transcripts in the Discovery Cohort quantified by RNAseq.**

| | **Gene expression in log2 RPKM** | | | | |
|---|---|---|---|---|---|
| **GeneName** | **[LOW]** | **[MID+]** | **Symbol** | **Description** | **RefSeq** |
| uc010ugx.2 | 4.17 | 4.14 | AQP9 | aquaporin 9 | NM_020980 |
| uc010rva.2 | 0.01 | 0.24 | CASP5 | caspase 5, apoptosis-related cysteine peptidase | NM_004347 |
| uc001pss.2 | 4.23 | 3.93 | CD3D | CD3d molecule, delta | NM_000732 |
| uc001quo.2 | 3.19 | 3.15 | CLEC4E | C-type lectin domain family 4, member E | NM_014358 |
| uc061sid.1 | 2.39 | 2.70 | CXCR2 | chemokine (C-X-C motif) receptor 2 | NM_001168298 |
| uc002tbx.4 | 2.13 | 2.11 | IL18RAP | interleukin 18 receptor accessory protein | NM_003853 |
| uc001ovc.4 | 3.89 | 4.00 | KCNE3 | potassium channel, voltage gated subfamily E regulatory beta subunit 3 | NM_005472 |
| uc001qye.4 | 2.12 | 2.06 | KLRC4 | killer cell lectin-like receptor subfamily C, member 4 | NM_013431 |
| uc003apy.5 | 2.91 | 3.03 | NCF4, | neutrophil cytosolic factor 4, 40kDa | NM_000631 |
| uc002qkv.4 | 1.08 | 0.96 | RPL28 | ribosomal protein L28 | NM_000991 |
| uc001fbr.2 | 5.56 | 5.86 | S100A12 | S100 calcium binding protein A12 | NM_005621 |
| uc001fbs.3 | 8.68 | 8.78 | S100A8 | S100 calcium binding protein A8 | NM_002964 |
| uc001fwq.5 | 0.06 | 0.22 | SLAMF7 | SLAM family member 7 | NM_021181 |
| uc004bjz.5 | 4.04 | 3.98 | TLR4 | toll-like receptor 4 | NM_138554 |
| uc002jup.3 | 2.86 | 2.74 | TMC8 | transmembrane channel-like 8 | NM_152468 |
| uc002txk.3 | 2.69 | 2.88 | TNFAIP6 | tumor necrosis factor, alpha-induced protein 6 | NM_007115 |
| uc003xcy.4 | 5.34 | 5.45 | TNFRSF10C | tumor necrosis factor receptor superfamily, member 10c, decoy | NM_003841 |
| | | | | | |
| **Mean** | **3.26** | **3.31** | | | |
| **t test p value** | **0.94** | | | | |
